# Synthesis, Antidepressant-like and Anxiolytic-like Effects of Novel Thiadiazole Derivatives: Behavioral Assessment and Mechanistic Investigation

**DOI:** 10.3390/ph19050797

**Published:** 2026-05-19

**Authors:** Ümmühan Kandemir, Gizem Türkoğlu Sağlık, Derya Osmaniye, Zafer Asım Kaplancıklı, Özgür Devrim Can, Ümide Demir Özkay

**Affiliations:** 1 Department of Medical Pharmacology, Faculty of Medicine, Bilecik Şeyh Edebali University, 11100 Bilecik, Türkiye; ummuhan.kandemir@bilecik.edu.tr; 2 Department of Pharmacology, Faculty of Pharmacy, Anadolu University, 26470 Eskişehir, Türkiye; gizemts@anadolu.edu.tr (G.T.S.); ozgurdt@anadolu.edu.tr (Ö.D.C.); 3 Department of Pharmaceutical Chemistry, Faculty of Pharmacy, Anadolu University, 26470 Eskişehir, Türkiye; dosmaniye@anadolu.edu.tr (D.O.); zakaplan@anadolu.edu.tr (Z.A.K.); 4 Central Research Laboratory, Faculty of Pharmacy, Anadolu University, 26470 Eskişehir, Türkiye

**Keywords:** thiadiazole, antidepressant-like, anxiolytic-like, motor activity, in silico studies

## Abstract

**Background/Objectives**: Based on the central nervous system-related activity potential of 1,3,4-thiadiazole derivatives, novel 1,3,4-thiadiazole compounds were synthesized, and their possible antidepressant-like and anxiolytic-like effects were investigated. **Methods**: The chemical structures of the compounds were elucidated using several spectroscopic techniques. Antidepressant-like effects of compounds were evaluated using the tail suspension and the modified forced swimming tests, while anxiolytic-like effects were assessed using the hole board, elevated plus maze, and open field tests in male Balb/c mice. Motor activities of the animals were examined using the activity-meter device. Mechanistic and computational in silico studies were also performed. **Results**: The results demonstrated that compounds **4e**–**4i** exhibited antidepressant-like effects, whereas only compound **4e** showed an anxiolytic-like effect. None of the compounds produced significant alterations in motor activities of animals, indicating that the observed behavioral effects were specific. The antidepressant-like effects of compounds **4e**–**4i** were abolished by *p*-chlorophenylalanine methyl ester (PCPA) and α-methyl-para-tyrosine methyl ester (AMPT) pre-administration indicating that the antidepressant-like effects of these test compounds are related to both serotonergic and catecholaminergic mechanisms. Furthermore, the anxiolytic-like effect of compound **4e** was reversed by flumazenil and NAN-190 pre-administrations, indicating the participation of the GABA-A benzodiazepine receptor complex and 5-HT_1A_ receptors in its pharmacological activity. Computational in silico studies revealed that compounds have good ADME profiles; compounds **4e**–**4i** interact with the serotonin transporter; compound **4e** shows affinity for GABA-A and 5-HT_1A_ receptors; and all interactions remain stable under dynamic conditions. **Conclusions**: These findings supported the previous papers reporting the antidepressant-like and anxiolytic-like effects of 1,3,4-thiadiazole derivatives.

## 1. Introduction

Anxiety and depressive disorders are the most prevalent mental health conditions. According to the World Health Organization, in 2021, approximately 359 million people were living with an anxiety disorder, while 280 million people were suffering from depression. In 2020, the prevalence of anxiety and depressive disorders significantly increased due to the COVID-19 pandemic. As a result of the pandemic, anxiety and major depressive disorders are estimated to have risen by 26% and 28%, respectively, within just one year [1,2]. Treatment options for anxiety and depressive disorders include both psychotherapy and pharmacotherapy. Although numerous antidepressants and anxiolytic medications are available on the market, these pharmacotherapies have certain limitations. Factors such as the high rate of patients who do not respond to medication and the side effects of drugs often lead to the discontinuation of treatment [3,4]. Therefore, researchers aim to discover and develop new pharmacological therapies for anxiety and depressive disorders.

The thiadiazole ring is a five-membered heterocyclic structure that contains both sulfur and nitrogen atoms. There are four isomeric forms of thiadiazoles: 1,2,3-thiadiazole, 1,2,4-thiadiazole, 1,2,5-thiadiazole, and 1,3,4-thiadiazole. Of these, 1,3,4-thiadiazole is the most frequently utilized in drug development research [5]. 1,3,4-thiadiazoles are known to possess a wide range of pharmacological activities. Research has shown that compounds containing this ring system demonstrate several pharmacological effects, including anticancer [6], antibacterial [5,7], antifungal [7], antiviral [8], antileishmanial [9], and anti-inflammatory [10,11] activities.

Numerous studies have highlighted the pharmacological effects of thiadiazole derivatives on the central nervous system (CNS). Some compounds featuring this ring system have been shown to exhibit antidepressant-like [12,13,14,15,16,17,18], anxiolytic-like [13,14,15,18,19], analgesic [10,11,16,20], and anticonvulsant [13,14,15] effects. Some findings from research on the potential biological targets of thiadiazole derivatives are noteworthy. For example, some thiadiazole derivative compounds have been reported to affect the serotonin [17] and noradrenalin neurotransmissions [21]. Moreover, some 1,2,5-thiadiazole derivatives have been reported to exhibit high affinity and selectivity for serotonin 5-HT_1A_ receptors, a significant drug target in the CNS. These derivatives have been shown to exhibit both antagonist and agonist properties, with some demonstrating strong activity even at the nanomolar level (IC_50_ = 2.3 nM) [22]. Recent studies, in particular, have shown that these compounds may also have effects on the GABAergic system. Indeed, derivatives containing 1,3,4-thiadiazole have been reported to exhibit significant anticonvulsant activity by acting via the GABAergic system. Furthermore, these derivatives have been reported to provide a high percentage of protection within certain dose ranges. Additionally, the docking scores of these compounds ((*S*)-(−)-2-[1-(4-fluorobenzoylamino)-2-phenylethyl]-5-(4-substitutedphenylamino)-1,3,4-thiadiazole) on the GABA-A receptor ranged from −5.52 to −6.65 kcal/mol. This is quite close to the reference compound diazepam (−5.66 kcal/mol) [23].

Based on the reported CNS-related pharmacological activity potential of compounds containing thiadiazole groups, in this study we planned to synthesize novel original thiadiazole derivative compounds and evaluate their potential effects on emotional behavior. Since behavioral experiments with rodents are fundamental steps for further neurological/molecular studies, in this study, antidepressant-like and anxiolytic-like effects were investigated using a comprehensive experimental design incorporating tests with well-known predictive validity and high reliability. In addition, the probable mechanisms underlying the pharmacological effects of the active compounds, as well as the relationships between the effective compounds and their potential sites of action, were investigated through mechanistic and in silico studies, respectively.

## 2. Results and Discussion

### 2.1. Chemistry

In this study, the thiadiazole core was chosen as the basic structure because it has been associated with numerous biological activities affecting the CNS in the literature. Structural modifications to this core are known to have significant effects on the biological activity and pharmacokinetic properties of the compounds.

The primary reason for selecting the methylsulfonyl group attached to the phenyl ring is that this group, thanks to its strong electron-withdrawing properties, modulates the electrostatic properties of the molecule and enhances interactions with target proteins (especially hydrogen bonding and dipole interactions). Furthermore, it was predicted that sulfonyl groups could positively contribute to solubility and bioavailability by increasing polarity.

The selection of different substituents attached to the amine group (alkyl, cycloalkyl, and aryl derivatives) was planned to systematically examine the structure-activity relationship. The aim was to investigate how the interactions of these substituents with the active sites of target proteins change due to the diversity in steric volume, lipophilicity, and electronic properties of these substituents. It has been hypothesized that aromatic substituents can enhance π–π interactions, while aliphatic chains can strengthen hydrophobic interactions. The synthesis method chosen for this purpose was preferred due to its high yield, operational ease, and compatibility with different substituted amines. This approach allowed for the easy creation of structural diversity and enabled the efficient production of the designed derivatives.

Accordingly, the fundamental hypothesis of the design strategy is that the biological activity of the thiadiazole core can be modified with suitable substituents to obtain novel derivatives capable of establishing stronger and more selective interactions with target proteins.

The structures of the synthesized thiadiazole derivatives (**4a**–**4l**) were confirmed by FT-IR, ^1^H NMR, ^13^C NMR, and HRMS analyses. In the FT-IR spectra, the characteristic N–H stretching vibrations observed in the range of 3165–3336 cm^−1^ confirm the presence of the secondary amine group. The observation of strong absorption bands belonging to the carbonyl (C=O) group in the range of 1653–1749 cm^−1^ indicates the successful formation of the target ketone structure.

In the ^1^H NMR spectra, the singlet signal observed at approximately δ ~ 4.00 ppm for all compounds corresponds to the methylene (-CH_2_-) group linking the thiadiazole and phenyl rings. The N–H proton was observed as a singlet or broad singlet (br.s.) in the range of δ ~ 7.2–10.5 ppm, depending on the substituent structure. Aromatic protons belonging to the para-disubstituted phenyl ring appeared as multiplet or doublet signals in the δ ~ 7.0–8.0 ppm range, consistent with the proposed structures.

Differences in substituents within the series were also reflected in the spectra. For example, proton signals belonging to alkyl groups were observed in the δ ~ 0.88–3.40 ppm range, while in the allyl derivative compound **4d**, characteristic signals belonging to olefinic protons were determined in the δ ~ 5.10–5.94 ppm range. In derivatives containing a methoxy group, the signal belonging to the –OCH_3_ group was observed as a singlet around δ ~ 3.86 ppm.

In the ^13^C NMR spectra, signals belonging to carbonyl carbons were observed in the δ ~ 165–171 ppm range, with aromatic and heteroaromatic carbons located in the expected regions. Signals belonging to aliphatic carbons supported the presence of the relevant substituents.

**Figure 1:**
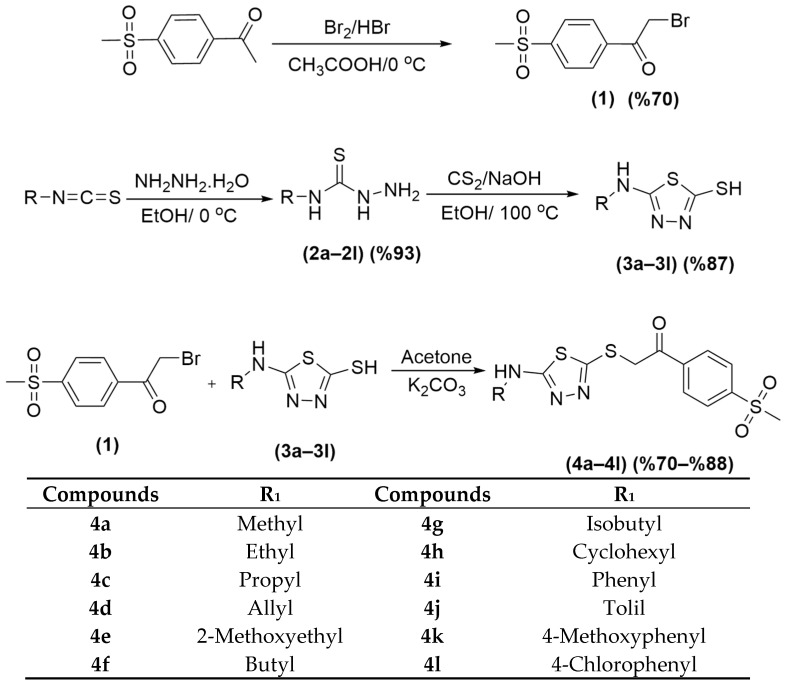
General procedure of the synthesis of targeted compounds.

The compounds designed to be investigated for their biological activity were obtained by four reaction steps. First, bromination reaction was carried out to synthesize 2-bromo-1-(4-(methylsulfonyl) phenyl)etan-1-one (**1**). In the second step, *N*-substitutedhydrazinecarbothioamides (**2a**–**2l**) were obtained by dropwise addition of hydrazine hydrate to the solution of substituted isothiocyanates in ethanol. In the third step, to obtain 5-(substitutedamino)-1,3,4-thiadiazole-2-thiol derivatives (**3a**–**3l**), compounds **2a**–**2l** were subjected to ring closure reaction using carbon sulfide under basic conditions. In the last step, thiadiazoles (**3a**–**3l**) were reacted with 2-bromo-1-(4-(methylsulfonyl)phenyl)ethan-1-one (**1**) to obtain the resulting compounds (**4a**–**4l**). The synthesis scheme for obtaining the target compounds is given in Figure 1.

### 2.2. Pharmacology

The objective of this study was to evaluate the potential antidepressant-like and anxiolytic-like effects of newly synthesized 1,3,4-thiadiazole derivative compounds and to obtain insight into their possible mechanisms of action. In this context, a comprehensive experimental plan was designed. To assess antidepressant-like and anxiolytic-like activities of the compounds, well-established and highly predictive behavioral models commonly used in this field were employed [16,24,25,26,27,28]. Initially, the antidepressant-like effects of the test compounds were assessed using the tail suspension and the modified forced swimming tests. Subsequently, anxiety-related behaviors in rodents were evaluated via the hole board, elevated plus maze, and open field tests. To rule out non-specific effects, such as sedation or stimulation that could confound behavioral outcomes, motor performance of the animals was also evaluated with activity-meter device. In the final stage of the study, mechanistic experiments and in silico analyses were performed to elucidate the underlying mechanisms of the observed in vivo effects.

**Figure 2:**
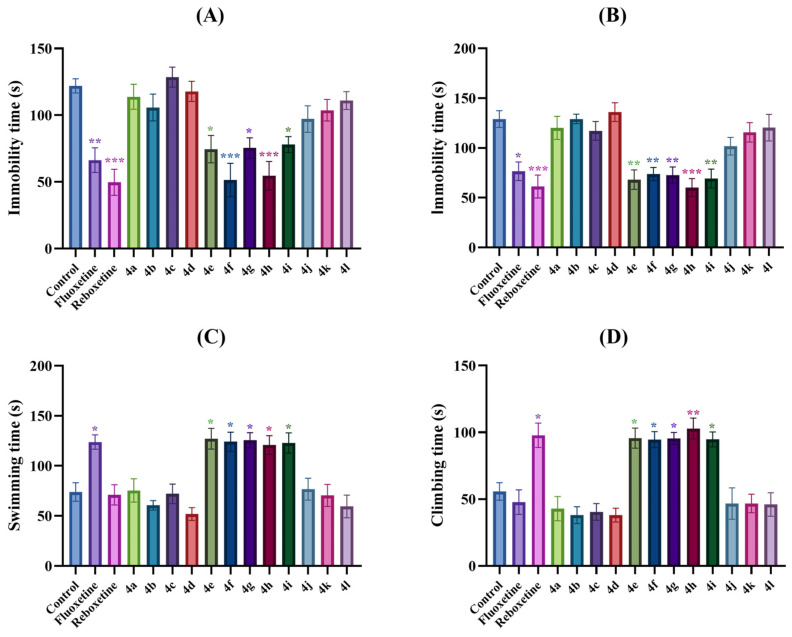
The effects of the control solution, fluoxetine (10 mg/kg), reboxetine (20 mg/kg) and test compounds (**4a**–**4l**) (30 mg/kg) administrations on the depression levels of animals. (**A**) immobility time of the animals in the tail suspension test, (**B**) immobility, (**C**) swimming and (**D**) climbing times of the animals in the modified forced swimming test. Significant difference compared to the control group * *p* < 0.05, ** *p* < 0.01, *** *p* < 0.001. One-way analysis of variance followed by Tukey HSD multiple comparison test, *n* = 7.

Tail suspension and modified forced swimming tests allow rapid detection of potential antidepressant-like activities of agents/drugs [16,24]. In both tests, the animals exhibit a motionless posture following initial escape-oriented movements. This state of immobility is referred to as ‘behavioral helplessness,’ which is thought to mimic aspects of human depression. Therefore, it is accepted that the decrease in the immobility times/frequency of the animals in the tail suspension and modified forced swimming tests indicates an antidepressant-like effect [25,29,30,31]. In this study, the effects of the test compound administration on immobility times of mice in the tail suspension [F (14,90) = 9.26, *p* < 0.001] and modified forced swimming [F (14,90) = 8.89, *p* < 0.001] tests are represented in Figure 2A and Figure 2B, respectively. One-way ANOVA analysis followed by Tukey’s honestly significant difference (HSD) multiple comparison tests revealed that administrations of test compounds **4e**, **4f**, **4g**, **4h** and **4i** and the reference drugs fluoxetine and reboxetine caused a statistically significant decrease in the immobility time of mice compared to the control group in both tests, indicating their antidepressant-like activities.

The modified forced swimming test is a variant of Porsolt’s forced swimming test. In this modified version, the water depth is increased to prevent the animals’ feet or tails from touching the bottom or sides of the cylinder and getting support from these surfaces. This test enables the evaluation of active behaviors of the animals such as swimming and climbing, which are known to be associated with monoaminergic neurotransmission in the CNS. In this study, the effects of the test compounds on swimming [F (14,90) = 9.49, *p* < 0.001] and climbing [F (14,90) = 12.48, *p* < 0.001] times of the mice are presented in Figure 2C and Figure 2D, respectively. Statistical analysis revealed that, fluoxetine, a serotonergic antidepressant, significantly increased swimming duration, whereas reboxetine, a noradrenergic antidepressant, significantly increased climbing behavior, thereby confirming the predictive validity of the test. Moreover, test compounds **4e**, **4f**, **4g**, **4h** and **4i** significantly increased the durations of both active behaviors, suggesting that both serotonergic and catecholaminergic systems may mediate their antidepressant-like effects [31,32,33,34].

In this study, the anxiolytic-like effects of the synthesized compounds were also assessed. To this end, hole board, elevated plus maze, and open field tests were performed.

**Figure 3:**
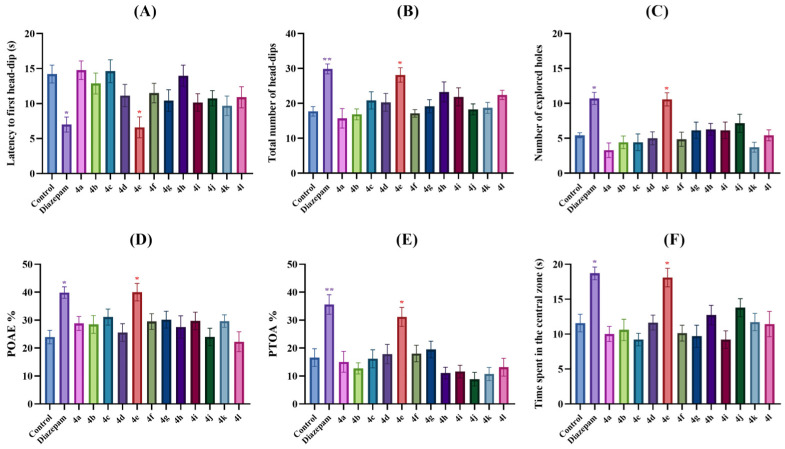
The effects of the control solution, diazepam (1 mg/kg) and test compounds (**4a**–**4l**) (30 mg/kg) administrations on the anxiety levels of animals. (**A**) latency to the first head-dip, (**B**) total number of head-dips and (**C**) number of explored holes in the hole board test, (**D**) POAE% and (**E**) PTOA% values of the mice in the elevated plus maze test, (**F**) time spent in the central zone of the animals in the open field test. Significant difference compared to the control group * *p* < 0.05, ** *p* < 0.01. One-way analysis of variance followed by Tukey HSD multiple comparison test, *n* = 7.

Figure 3A, Figure 3B and Figure 3C, respectively, display the effects of the test compound administration on latencies to the first head-dip [F (13,84) = 3.37, *p* < 0.001], total number of head-dips [F (13,84) = 4.21, *p* < 0.001], and number of explored holes [F (13,84) = 5.29, *p* < 0.001] in the hole board test. Multiple comparison analyses revealed that test compound **4e** and reference drug diazepam significantly reduced the first head-dip latencies of mice compared to the control group. On the other hand, test compound **4e** and diazepam significantly enhanced the head-dip and number of explored holes in animals. These results indicated that compound **4e** enhanced exploratory behavior in the animals, in other words, it showed anxiolytic-like effect [25,26,35,36].

The elevated plus maze test, another test used to assess anxiety behavior in animals, is based on the principle that the animals avoid narrow, open and high arms (fear of an unfamiliar open space and fear of balancing on a relatively narrow, elevated platform) and prefer dark and closed arms due to their natural tendency. It is well established that anxiolytic drugs/agents increase the percentage of open arm entries (POAE%) and percentage of time spent in the open arm (PTOA%) values, whereas anxiogenic agents produce the opposite effect [25,26,37]. In this study, the effects of the test compound administration on POAE% [F (13,84) = 3.07, *p* < 0.001] and PTOA% [F (13,84) = 6.68, *p* < 0.001] values calculated in the elevated plus maze tests are exhibited in Figure 3D and Figure 3E, respectively. The findings revealed that diazepam used as the reference drug and test compound **4e** significantly increased the POAE% and PTOA% values compared to the control group indicated that compound **4e** exhibits an anxiolytic-like effect.

In the open field test, the natural tendency of rodents, called “thigmotaxis,” is to prefer the peripheral area of the setup and walk close to the walls. Increased activity of rodents in the central area of the apparatus is generally associated with increased exploratory behavior and decreased anxiety levels of the animals, while reduced activity of rodents in the central area relates to enhanced anxiety levels of the animals [27,28]. In this study, only test compound **4e** significantly increased the time spent in the central area of the apparatus, reduced thigmotaxis [F (13,84) = 5.36, *p* < 0.001] (Figure 3F) and showed an anxiolytic-like effect.

Reference drug diazepam showed the expected anxiolytic activity in all anxiety tests conducted in this study (Figure 3).

**Figure 4:**
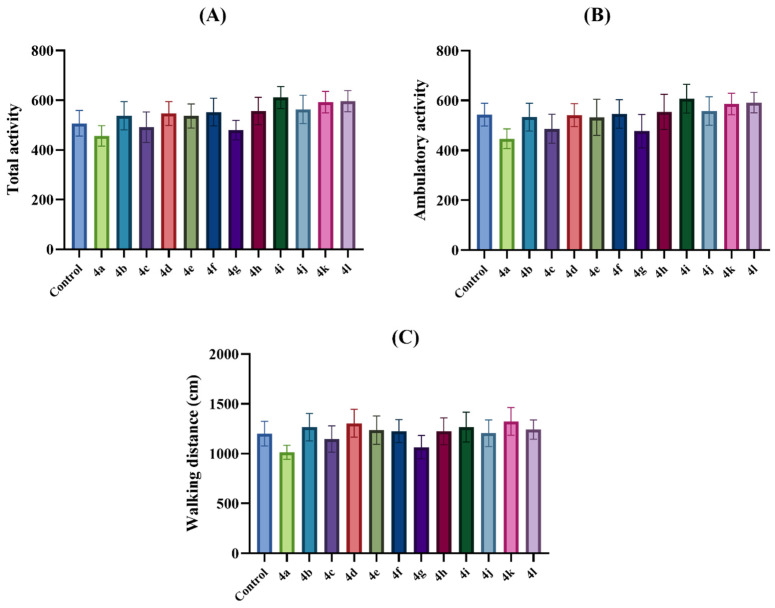
The effects of the control solution and test compound (**4a**–**4l**) (30 mg/kg) administration on the motor activities of animals. (**A**) total activity, (**B**) ambulatory activity, and (**C**) walking distance of the animals in the activity-meter test. One-way analysis of variance followed by Tukey HSD multiple comparison test, *n* = 7.

In this study, activity-meter tests were performed to evaluate the effects of the test compounds on the motor performance of mice [38]. The effects of the test compound administration on total activity number [F (12,78) = 0.86, *p* > 0.05], ambulatory activity [F (12,78) = 0.68, *p* > 0.05] and walking distance [F (12,78) = 0.48, *p* > 0.05] values in the activity-meter tests are shown in Figure 4A, Figure 4B and Figure 4C, respectively. ANOVA tests results indicated that test compound administration did not cause significant change in the total activity number, ambulatory activity or walking distance of mice. These findings showed that the behavioral outcomes observed in this study were not associated with any changes in motor activity of the animals and that the antidepressant-like and anxiolytic-like effects of the test compounds were specific.

In the subsequent phase of the study, experiments were carried out to clarify the mechanisms underlying the antidepressant-like effects of compounds **4e**, **4f**, **4g**, **4h** and **4i**, and anxiolytic-like effect of compound **4e**.

The monoaminergic system plays a crucial role in mood regulation. Dysfunctions in serotonergic and/or catecholaminergic systems are closely linked to the pathogenesis of depression. Moreover, majority of currently used antidepressant drugs in clinical practice also increase the levels of monoamines in the synaptic cleft [39]. Based on this knowledge and the findings from modified forced swimming tests that highlight the involvement of serotonergic and catecholaminergic systems in the antidepressant-like effects of test compounds **4e**, **4f**, **4g**, **4h** and **4i**, mechanistic studies were performed using *p*-chlorophenylalanine methyl ester (PCPA) and α-methyl-para-tyrosine methyl ester (AMPT), agents that deplete monoamines in the CNS. The tail suspension test was chosen for mechanistic studies, as it offers several advantages over the modified forced swimming test, including absence of hypothermia risk, higher sensitivity to a broader range of antidepressant drugs, and a quicker recovery of animals to their normal spontaneous activity following the experiment [24,30].

In order to investigate the participation of the serotonergic system in the antidepressant-like effect of test compounds **4e**, **4f**, **4g**, **4h** and **4i**, experiments were conducted with PCPA, a chemical agent that inhibits serotonin synthesis in the CNS by blocking the tryptophan hydroxylase enzyme, thereby depleting serotonin stores in nerve endings. Previous studies have shown that PCPA administered to mice at a dose of 100 mg/kg for four days resulted in a reduction in central serotonin stores by 60–90%, without affecting levels of noradrenaline and dopamine [40,41,42].

The contribution of the catecholaminergic system to the antidepressant-like effect of test compounds **4e**, **4f**, **4g**, **4h** and **4i** was examined using AMPT, an agent that inhibits the synthesis of noradrenaline and dopamine by blocking the tyrosine hydroxylase enzyme in the CNS. It has been stated that AMPT administered at a dose of 100 mg/kg reduces noradrenaline and dopamine levels by approximately 53% and 57%, respectively, without affecting central serotonin levels [43,44].

**Figure 5:**
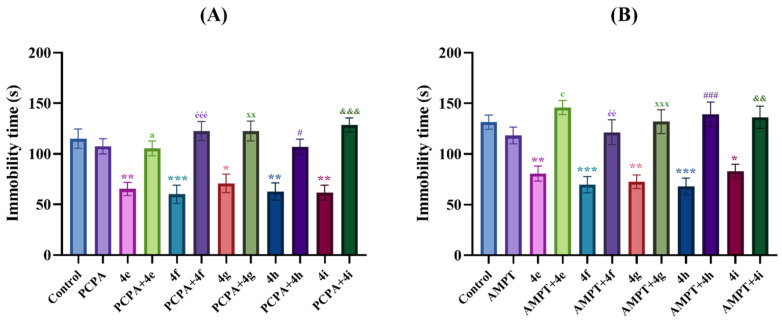
The effects of the PCPA (**A**) and AMPT (**B**) pre-administration on compounds **4e**, **4f**, **4g**, **4h** and **4i** induced antidepressant-like effects in the tail suspension test. Significant difference compared to the control group * *p* < 0.05, ** *p* < 0.01, *** *p* < 0.001; Significant difference compared to the compound **4e** administrated group ^a^ *p* < 0.05, ^c^ *p* < 0.001; Significant difference compared to the compound **4f** administrated group ^éé^ *p* < 0.01, ^ééé^ *p* < 0.001; Significant difference compared to the compound **4g** administrated group ^xx^ *p* < 0.01, ^xxx^ *p* < 0.001; Significant difference compared to the compound **4h** administrated group ^#^ *p* < 0.05, ^###^ *p* < 0.001; Significant difference compared to the compound **4i** administrated group ^&&^ *p* < 0.01, ^&&&^ *p* < 0.001. One-way analysis of variance followed by Tukey HSD multiple comparison test, *n* = 7.

The effects of PCPA [F (11,72) = 10.85, *p* < 0.001] and AMPT [F (11,72) = 11.27, *p* < 0.001] pre-administration on immobility times in the tail suspension test in mice receiving the control solution and the test compounds **4e**, **4f**, **4g**, **4h** and **4i** are presented in Figure 5A and Figure 5B, respectively. One-way ANOVA analysis followed by Tukey HSD multiple comparison tests revealed that PCPA and AMPT pre-treatments significantly reversed the decreased immobility time of mice induced by test compounds **4e**, **4f**, **4g**, **4h** and **4i**. These data indicated that both serotonergic and catecholaminergic systems play a role in the antidepressant-like effects of these compounds.

In the series, test compound **4e** exhibited not only antidepressant-like activity but also anxiolytic-like effect. Therefore, mechanistic studies were also performed using benzodiazepine receptor antagonist flumazenil and the selective 5-HT_1A_ receptor antagonist NAN-190 to investigate the role of GABAergic and serotonergic systems [45,46], which are known to play important roles in anxiety, in the anxiolytic-like effect of test compound **4e**.

**Figure 6:**
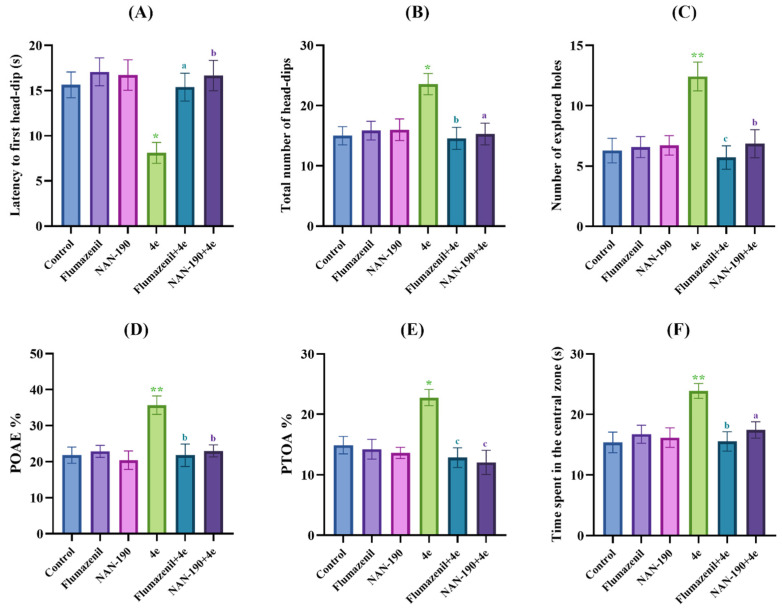
The effects of the flumazenil and NAN-190 pre-administration on compound **4e** induced anxiolytic-like effect. (**A**) latency to the first head-dip, (**B**) total number of head-dips and (**C**) number of explored holes in the hole board test, (**D**) POAE% and (**E**) PTOA% values of the mice in the elevated plus maze test, (**F**) time spent in the central zone of the animals in the open field test. Significant difference compared to the control group * *p* < 0.05, ** *p* < 0.01; Significant difference compared to the compound **4e** administrated group ^a^ *p* < 0.05, ^b^ *p* < 0.01, ^c^ *p* < 0.001. One-way analysis of variance followed by Tukey HSD multiple comparison test, *n* = 7.

Figure 6A, Figure 6B and Figure 6C, respectively, show the effects of pre-treatments of the mice with flumazenil and NAN-190 on the latencies to the first head-dip [F (5,36) = 5.05, *p* < 0.01], total number of head-dips [F (5,36) = 3.96, *p* < 0.01] and total number of explored holes [F (5,36) = 6.01, *p* < 0.001] of animals in hole board test. Post hoc analyses indicated that the pre-treatments of mice with flumazenil and NAN-190 were effective in reversing the decreased latencies to the first head-dip of animals treated with compound **4e**. Moreover, flumazenil and NAN-190 pre-treatments were blocked the compound **4e**-induced increase in the total number of head-dips and total number of explored holes.

The effects of the flumazenil and NAN-190 pre-treatments on POAE% [F (5,36) = 5.78, *p* < 0.001] and PTOA% [F (5,36) = 6.51, *p* < 0.001] values in the elevated plus maze tests are shown in Figure 6D and Figure 6E, respectively. Post hoc analyses showed that pre-treatments of the mice with flumazenil and NAN-190 effectively abolished the increased POAE% and PTOA% values of animals.

Figure 6F displays the effects of the flumazenil and NAN-190 pre-treatments on the percentage of time spent by the animals in the central zone during the open field tests [F (5,36) = 4.53, *p* < 0.01]. Results of the Tukey HSD multiple comparison test indicated that pre-treatments of the mice with flumazenil and NAN-190 decreased the enhanced duration spent in the central zone.

GABA, which is among the neuromediators that play a role in anxiety, is the main inhibitory neurotransmitter of the CNS [47]. Anxiolytic drugs, mostly from the benzodiazepine class, are commonly prescribed for the treatment of anxiety [48]. Benzodiazepines increase GABA function by directly interacting with the allosteric benzodiazepine site of the GABA-A benzodiazepine receptor Cl^−^ channel complex [49]. In this study, mechanistic studies revealed that flumazenil pre-treatment reversed the anxiolytic-like effect of compound **4e**. These findings indicated that the GABA(A)/benzodiazepine receptor complex may play a role in the anxiolytic-like effect of the test compound **4e**.

Another system identified to be of great importance in regulating emotional states and behaviors is the serotonergic system. It is known that serotonin, especially 5-HT_1A_ receptors widely distributed in the hippocampus, dorsal raphe nucleus and amygdala, plays a role in the regulation of emotional state and behavior [50,51,52]. Indeed, buspirone is an anxiolytic drug that exerts its pharmacological effect via partial agonism of the 5-HT_1A_ receptor [53]. In this study, results revealed that the anxiolytic-like effect of test compound **4e** was reversed by pre-treatment of NAN-190. This finding indicated that 5-HT_1A_ receptors as well as GABA(A)/benzodiazepine receptor complex participate in the anxiolytic-like effect of test compound **4e**.

In this study, the tested compounds exhibited negligible acute toxicity, as neither mortality nor undesirable side effects such as ataxia, paralysis, convulsions, or diarrhea were detected in mice. These findings suggest a favorable preliminary safety profile. However, further detailed investigations are necessary to fully characterize the safety profile of these compounds.

### 2.3. Computational In Silico Studies

The pharmacokinetic properties of the synthesized compounds (**4a**–**4l**) were evaluated by in silico ADME analyses (Table 1). According to the obtained data, all compounds had molecular weights in the range of 343–440 Da and complied with Lipinski’s rule of five (rule of five violation = 0). This finding indicates that the compounds possess suitable properties in terms of potential oral bioavailability.

The number of hydrogen bond donors (donorHB ≈ 1) and acceptors (accptHB ≈ 8–10) of the compounds were within acceptable limits, providing an advantage in terms of passage through cell membranes. Polar surface area (PSA) values in the range of approximately 100–114 Å^2^ indicate that the compounds have moderate polarity and suggest a suitable balance in terms of bioavailability.

QPlogS values ranging from −3.6 to −5.7 indicate that the compounds have moderate water solubility. It is noteworthy that solubility is somewhat reduced, particularly in more lipophilic derivatives (e.g., **4h**, **4j**, and **4l**).

When QPlogBB values are examined (ranging from −1.2 to −1.9), it is predicted that the compounds may cross the blood–brain barrier to a limited extent. This supports the potential of the compounds for CNS targets, while also indicating that the risk of excessive accumulation may be low.

Human oral absorption percentage (PHOA) values ranging from 74 to 90% show that all compounds have high oral absorption potential. Higher absorption rates were observed, particularly in more lipophilic compounds such as **4h** and **4i**.

Overall, it can be said that the synthesized compounds have balanced ADME profiles, possess good oral bioavailability potential, and are promising candidates in terms of pharmacokinetics.

The binding scores of the proteins 7LWD, 6HUO, 7E2Z, and 4XNX, which were examined within the scope of molecular docking studies, are presented comparatively in Table 2, and the binding tendencies of the compounds were evaluated based on these data.

Based on the findings obtained from in vivo tests assessing the mechanism of antidepressant-like efficacy, it was suggested that the observed effects of the compounds **4e**, **4f**, **4g**, **4h** and **4i** may be mediated through the serotonergic and catecholaminergic systems. In line with these findings and monoamine hypothesis in depression [54], molecular modeling studies were performed using the crystal structures of monoamine transporters to elucidate whether the antidepressant-like effects of these compounds are associated with alterations in monoamine levels within the synaptic cleft.

**Figure 7:**
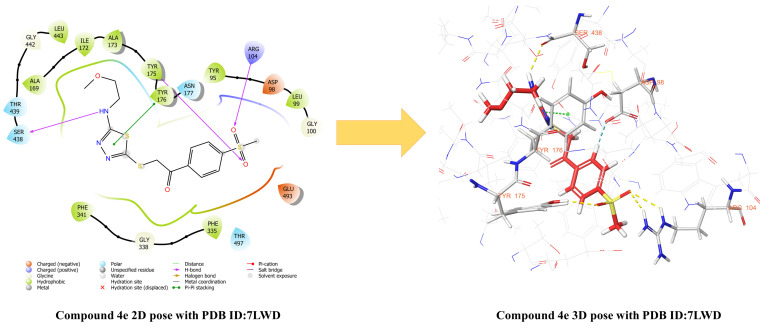
Two-dimensional (2D) and three-dimensional (3D) representation of the interaction of compound **4e** with the active site of the SERT (PDB ID: 7LWD).

**Figure 8:**
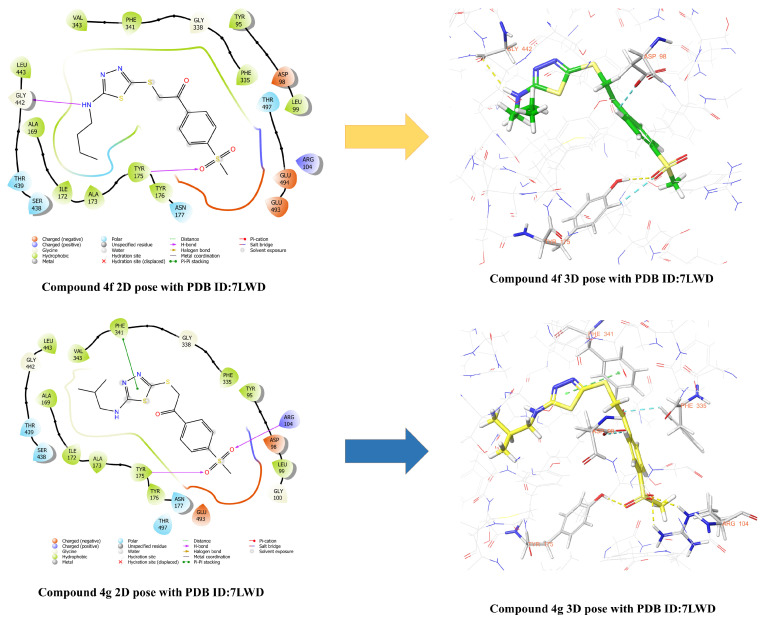
Two-dimensional (2D) and three-dimensional (3D) representation of the interactions of compounds **4f** and **4g** with the active site of the SERT (PDB ID: 7LWD).

**Figure 9:**
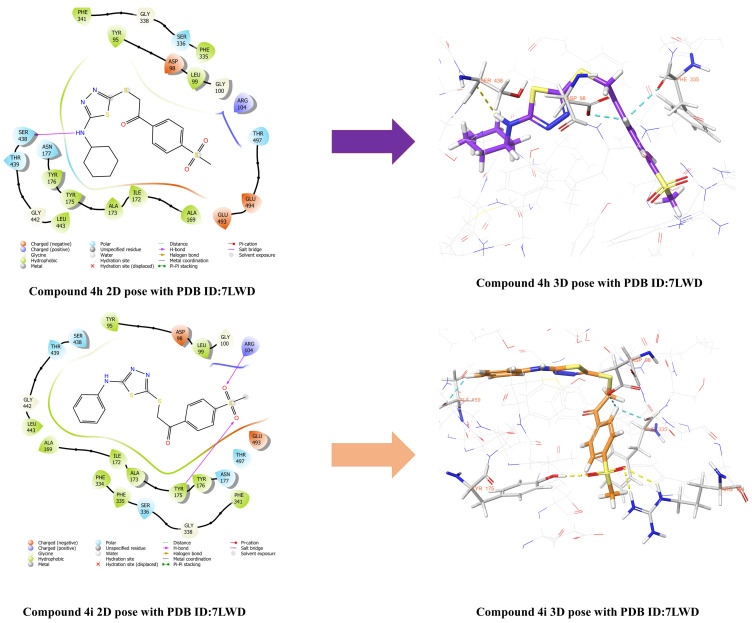
Two-dimensional (2D) and three-dimensional (3D) representation of the interactions of compounds **4h** and **4i** with the active site of the SERT (PDB ID: 7LWD).

Molecular docking studies were initiated using the crystal structure of the SERT, which is widely employed in investigation of the mechanisms underlying antidepressant action of agents [55,56]. Figure 7, Figure 8 and Figure 9 demonstrate the two- (2D) and three-dimensional (3D) representation of the interactions of compounds **4e** (Figure 7), **4f**, **4g** (Figure 8), **4h** and **4i** (Figure 9) with the human SERT complexed of the 7LWD structure. The analyses demonstrated that the compounds exhibited the following interactions with the SERT crystal structure:

When the interactions of compound **4e** in the active site of the 7LWD crystal structure of the SERT protein were examined, it was observed that hydrogen bonds were formed between the sulfone group and the amino group of Arg104 amino acid, and between the hydroxyl group of Tyr175 amino acid. Furthermore, a hydrogen bond exists between the amino group of the compound and the carbonyl group of Ser438 amino acid. In addition, a π–π interaction was observed between the thiadiazole ring and the phenyl ring of Tyr176 amino acid, while an aromatic hydrogen bond interaction was detected between the phenyl ring and the carbonyl group of Asp98 amino acid. When the interactions of compound **4f** with the active site of the 7LWD crystal structure of the SERT protein were examined, it was observed that a hydrogen bond was formed between the sulfone group and the hydroxyl group of the amino acid Tyr175. In addition, an aromatic hydrogen bond interaction was detected between the phenyl ring of the compound and the carbonyl group of the amino acid Asp98. Analysis of the interactions between compound **4g** and the active site of the 7LWD crystal structure of the SERT protein revealed hydrogen bonds between the sulfone group and the amino group of Arg104 amino acid, and between the hydroxyl group of Tyr175 amino acid. In addition, a π–π interaction was observed between the thiadiazole ring and the phenyl ring of Phe341 amino acid. Furthermore, aromatic hydrogen bonding interactions were detected between the phenyl ring of the compound and the carbonyl group of Asp98 amino acid, and between the carbonyl group and the phenyl ring of Phe335 amino acid. When the interactions of compound **4h** with the active site of the 7LWD crystal structure of the SERT protein were examined, it was observed that a hydrogen bond formed between the amino group and the carbonyl group of the Ser438 amino acid. In addition, aromatic hydrogen bond interactions were detected between the phenyl ring of the compound and the carbonyl groups of the Asp98 and Phe335 amino acids. When the interactions of compound **4i** in the active site of the 7LWD crystal structure of the SERT protein were examined, it was observed that hydrogen bonds were formed between the sulfone group and the amino group of the Arg104 amino acid, and between the hydroxyl group of the Tyr175 amino acid. In addition, aromatic hydrogen bonds were detected between the 1,4-disubstituted phenyl ring and the carbonyl group of the Phe335 amino acid and the hydroxyl group of the Asp98 amino acid. Furthermore, the formation of aromatic hydrogen bonds was observed between the monosubstituted benzene ring and the hydroxyl group of the Ala69 amino acid.

Protein–ligand interactions of the reference ligand vilazodone, which has a 7LWD crystal structure, are presented in the Supplementary Materials. It was determined that the reference compound interacts with the amino acids Asp98, Arg104, Phe556, and Ser559. Examination of the obtained docking results revealed that the synthesized compounds were able to interact with the Arg104 residue, particularly via the methylsulfonyl group. This finding indicates that similar interaction regions to the reference ligand were targeted. However, it was considered that this interaction alone is not sufficient to explain biological activity, and that adaptation to the binding pocket, hydrophobic interactions, and the overall conformational positioning of the ligand also play important roles. Therefore, the interaction of the methylsulfonyl group with Arg104 can be considered an important factor contributing to the binding behavior of the compounds.

In conclusion, compounds **4f** and **4h** were found to interact exclusively with the S1 (orthosteric) binding site, whereas compounds **4e**, **4g**, and **4i** demonstrated strong binding to both the S1 and S2 (allosteric) sites. These findings suggest that compounds **4f** and **4h** may exhibit a classical SSRI-like effect profile, while compounds **4e**, **4g**, and **4i** possess the potential for dual-binding activity. Dual-binding compounds are generally considered to display a distinct and potentially superior pharmacological profile compared to classical SSRIs. These compounds not only compete directly with serotonin to inhibit reuptake but also, via the S2 site, slow the dissociation rate of the ligand bound to S1 and limit the conformational transitions of the transporter. Consequently, this dual mechanism may enable more sustained and potent inhibition of serotonin reuptake.

Molecular modeling studies were further conducted using the high-resolution crystal structure of the human dopamine transporter (DAT) (PDB ID: 4XNX), a key member of the monoamine transporter family. Due to its membership in the monoamine transporter family, DAT shares significant structural homology with the norepinephrine transporter (NET) [57,58]. The protein was optimized for docking by adding missing hydrogens, assigning protonation states, and performing energy minimization. Ligands were docked into the binding pocket, and their binding affinities and interactions were evaluated. Docking results, including hydrogen bonds, π–π stacking, and hydrophobic interactions (Supplementary Materials), suggest limited activity, as no interactions were detected with the critical residues Asp46 and Phe43.

**Figure 10:**
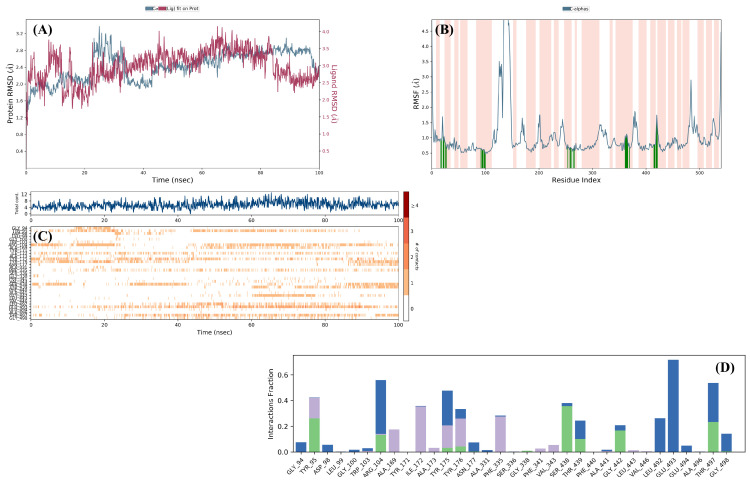
MD simulations results of complex **4e+7LWD**. (**A**) RMSD profiles of the MD simulations (100 ns) (**B**) RMSF profiles of the MD simulations (100 ns) (**C**) Amino acid interactions timeline graphics of complexes (**D**) Amino acid interactions histogram of complexes.

Molecular dynamics (MD) simulations were conducted for compound **4e**, which exhibited a distinct activity profile. The RMSD plot for the compound **4e+7LWD** complex over 100 ns is shown in Figure 10A. RMSD analysis indicates that the complex remained stable throughout the simulation, with compound **4e** maintaining a stable position within the SERT binding site.

To more reliably assess the time-dependent stability of ligand–protein complexes and the dynamics of binding interactions, MD simulations were performed using the high-resolution PDB ID: 7LWD crystal structure, which accurately represents the active-site conformation. Examination of the RMSF plot (Figure 10B) allowed the identification of amino acid residues interacting with compound **4e** and their corresponding fluctuation values as follows: Gly94 (0.93 Å), Tyr95 (0.92 Å), Ala96 (1.01 Å), Asp98 (1.12 Å), Leu99 (0.89 Å), Gly100 (1.04 Å), Trp103 (0.82 Å), Arg104 (0.73 Å), Ala169 (0.66 Å), Tyr171 (0.59 Å), Ile172 (0.60 Å), Ala173 (0.61 Å), Tyr175 (0.60 Å), Tyr176 (0.51 Å), Asn177 (0.47 Å), Gln332 (0.67 Å), Phe335 (0.67 Å), Ser336 (0.68 Å), Pro339 (0.55 Å), Phe341 (0.64 Å), Val343 (0.74 Å), Ser438 (1.06 Å), Thr439 (0.99 Å), Phe440 (1.01 Å), Ala441 (1.12 Å), Gly442 (1.04 Å), Leu443 (0.84 Å), Ile447 (0.66 Å), Leu492 (0.77 Å), Glu493 (0.79 Å), Glu494 (0.76 Å), Ala496 (1.32 Å), Thr497 (1.75 Å), Gly498 (1.39 Å), and Pro499 (1.13 Å).

Figure 10C and Figure 10D illustrate interaction types (blue: water-mediated hydrogen bonds; green: direct hydrogen bonds; purple: hydrophobic interactions) and their time-dependent behavior. Analysis of the graph reveals that interactions, particularly with Arg104, Ser438, and Glu493, were frequent and persistent throughout the simulation. Moreover, examination of video 3 confirmed that the aromatic hydrogen bond with Asp98 is conserved. These findings support the conclusion that compound **4e** exhibits a robust dual-inhibition profile, engaging both the S1 and S2 binding sites within the SERT active region.

This study yielded findings suggesting that the antidepressant-like effects of the compounds **4e**, **4f**, **4g**, **4h** and **4i** may be primarily associated with inhibition of the SERT rather than the DAT. However, although molecular modeling studies provide valuable insights into the potential binding modes and interaction profiles of ligands with their target proteins, these approaches alone are not sufficient to establish definitive mechanisms of action. Therefore, additional experimental validation is required to draw firm conclusions regarding the underlying pharmacological mechanisms of the compounds. In this context, radioligand binding assays and functional uptake inhibition studies could be performed to confirm direct interactions with SERT. Moreover, the determination of central monoamine levels using reliable analytical techniques would help clarify whether the test compounds increase synaptic monoamine levels. On the other hand, considering the well-established involvement of monoaminergic receptors in the etiopathogenesis of depression and in the antidepressant response [24,59], it is also essential to investigate the potential contribution of receptor subtypes (e.g., 5-HT_1A_, 5-HT_2A_, D_2_, α_1_-adrenergic receptors) to the observed antidepressant-like effects. Furthermore, other endogenous neuromodulatory systems, such as opioidergic, GABAergic, glutamatergic, and nitrergic systems, may also have contributed to the antidepressant effect of these compounds. Therefore, comprehensive mechanistic studies are warranted to explore the role of these pathways in antidepressant-like effect in greater detail.

Antagonism studies conducted to elucidate the mechanism underlying the anxiolytic-like effect demonstrated that the effect of compound **4e** is mediated through GABAergic and 5-HT_1A_ receptors. To further characterize and support these findings, molecular modeling studies were performed to investigate the potential binding interactions of compound **4e** with these receptor targets.

**Figure 11:**
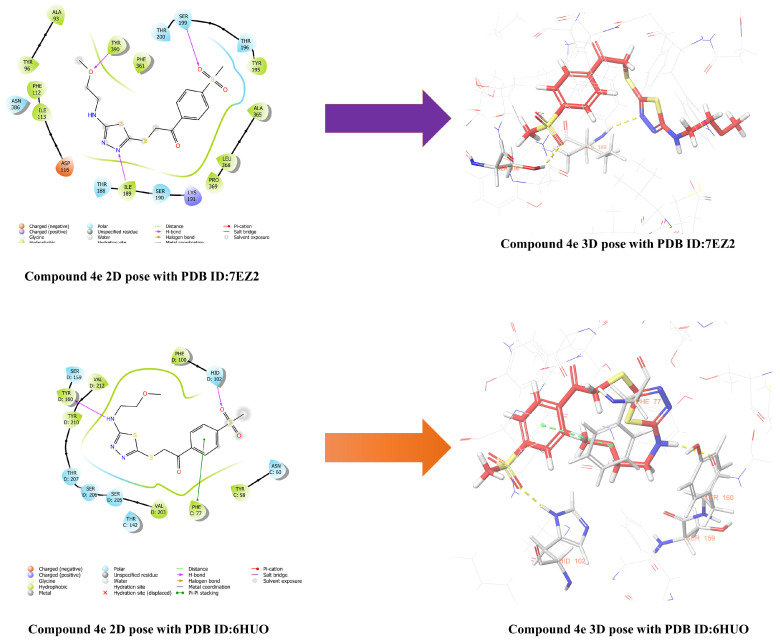
Two-dimensional (2D) and three-dimensional (3D) representation of the interactions of compound **4e** with the active site of the 5-HT_1A_ receptor (PDB ID: 7E2Z) and GABA-A receptor (PDB ID: 6HUO).

Molecular docking studies were initially performed using the crystal structure with PDB ID: 6HUO. The resulting docking poses are presented in Figure 11. The target protein in the PDB ID: 6HUO construct is the GABA-A receptor, with alprazolam as the reference ligand. Analysis of the docking results revealed that sulfone group of compound **4e** established hydrogen bonding with the imidazole ring of His102 residue, phenyl ring of compound **4e** a π–π stacking interaction with the phenyl ring of Phe77 residue, and amine group of compound **4e** formed an additional hydrogen bond with the carbonyl group of Tyr160 residue.

In addition to studies conducted with the GABA-A receptor, molecular docking studies were also performed using the PDB ID: 7E2Z crystal structure. This construct represents the aripiprazole-bound serotonin 5-HT_1A_ receptor-Gi protein complex. The 2D and 3D binding modes of compound **4e** within the active site of the 5-HT_1A_ receptor (PDB ID: 7E2Z) are presented in Figure 11. Analysis of the docking results revealed that the methylsulfonyl group of compound **4e** forms a hydrogen bond with the hydroxyl group of Ser199. Ser199 is known to play a critical role in maintaining the proper orientation of ligands within the binding pocket and in enhancing binding stability. Furthermore, the nitrogen atom of the thiadiazole ring was found to form a hydrogen bond with amine group of Ile189. This residue contributes to shaping the hydrophobic architecture of the ligand-binding pocket and plays an indirect role in partial agonism. It is thought that the interactions with this residue indirectly limit the outward movement of the TM6 helix, thus contributing to the support of a partial agonist profile instead of a full agonistic effect. Finally, the oxygen atom of the 2-methoxyethyl side chain of compound **4e** was observed to form a hydrogen bond with hydroxyl group of Tyr390. This interaction is considered to contribute to the stabilization of the conformational coupling between receptor activation and intracellular signal transduction.

Alprazolam was used as the reference ligand for the 6HUO crystal structure, and aripiprazole for the 7E2Z crystal structure. The docking positions of these drugs are presented in the Supplementary Materials. It was determined that alprazolam interacts with the residues Tyr58, Ser205, Tyr210, Tyr160, Phe100, and His102 in the 6HUO structure. Compound **4e** was observed to exhibit similar interactions with Tyr160 and His102. The interaction with His102, particularly through a methylsulfonyl group, is noteworthy and suggests that this functional group may contribute to interactions in the binding pocket. In the 7E2Z crystal structure, the reference ligand aripiprazole was found to interact with the residues Asp116, Tyr96, and Phe362. While compound **4e** did not directly exhibit these specific interactions in the docking results, it was found to be located within the active site and near the region containing the amino acids.

**Figure 12:**
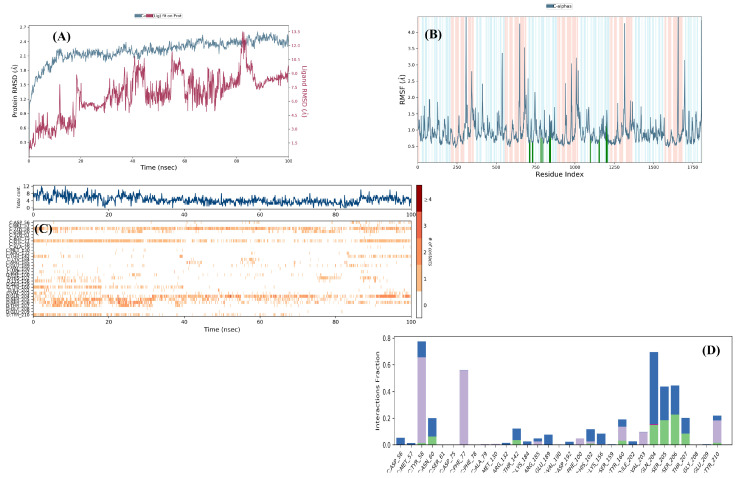
MD simulations results of complex **4e+6HUO**. (**A**) RMSD profiles of the MD simulations (100 ns) (**B**) RMSF profiles of the MD simulations (100 ns) (**C**) Amino acid interactions timeline graphics of complexes (**D**) Amino acid interactions histogram of complexes.

Following molecular modeling studies, MD simulations were also performed to investigate the stability of the interactions of compound **4e** with these receptors. Firstly, the stability of the interaction between compound **4e** and GABA-A receptor was examined under dynamic conditions. The RMSD profile obtained from the 100 ns MD simulation of the compound **4e**-6HUO complex is presented in Figure 12A. Analysis of the RMSD reveals that the complex maintained structural stability throughout the simulation period. Moreover, compound **4e** remained stably positioned within the benzodiazepine binding site of the GABA-A receptor. These findings, which are consistent with both the docking results and the in vivo activity data, support the high structural stability of the complex formed between compound **4e** and the 6HUO crystal structure.

When the RMSF graph (Figure 12B) is examined, the amino acid residues interacting with compound **4e** and their corresponding fluctuation values are determined as follows: His54 (0.62 Å), Asp56 (0.69 Å), Tyr58 (0.66 Å), Asn60 (0.76 Å), Ile62 (0.73 Å), Ile76 (0.65 Å), Phe78 (0.66 Å), Gln80 (0.61 Å), Leu131 (0.76 Å), Arg132 (0.82 Å), Arg144 (0.69 Å), Arg185 (1.42 Å), Ser187 (1.18 Å), Glu189 (0.76 Å), Thr193 (1.06 Å), Asp98 (0.92 Å), His104 (0.70 Å), Leu155 (0.63 Å), Ser159 (0.83 Å), Ala161 (1.09 Å), Ser200 (0.81 Å), Ile202 (1.07 Å), Val203 (1.09 Å), Ser205 (1.30 Å), and Thr207 (1.55 Å).

In addition to these interactions, further ligand–receptor contacts were observed during the MD simulation, as shown in video 2. Notably, aromatic hydrogen bonds were formed with the amino acid residues Tyr58, Gln204, and Asn60.

It is particularly noteworthy that His102 and Phe77, which are located within the active site of the GABA-A receptor, represent key residues contributing to strong ligand binding. Among these, the π–π stacking interaction with Phe77 persisted uninterrupted throughout the simulation, indicating a substantial contribution to overall complex stability. Although a π–π interaction with His102 was observed for approximately 20 ns, this interaction did not persist consistently over the entire simulation period. Furthermore, a π–π interaction was detected between the thiadiazole ring of compound **4e** and Tyr160. A hydrogen bond was formed between the methoxy group of the ligand and Ser205, while a double hydrogen bond was observed between the thiadiazole ring and Thr207. Additional hydrogen bonds were identified between the carbonyl group of the compound and Ser206, as well as between the sulfonyl group and Lys156. The types of these interactions and their time-dependent behavior are presented in Figure 12C and Figure 12D. Analysis of this graph demonstrates a high frequency and persistence of interactions, particularly with Tyr58, Phe77, Gln204, Ser206, and Thr207, throughout the simulation period. Collectively, these findings indicate that these residues play a critical role in stabilizing compound **4e** within the active site of the GABA-A receptor.

**Figure 13:**
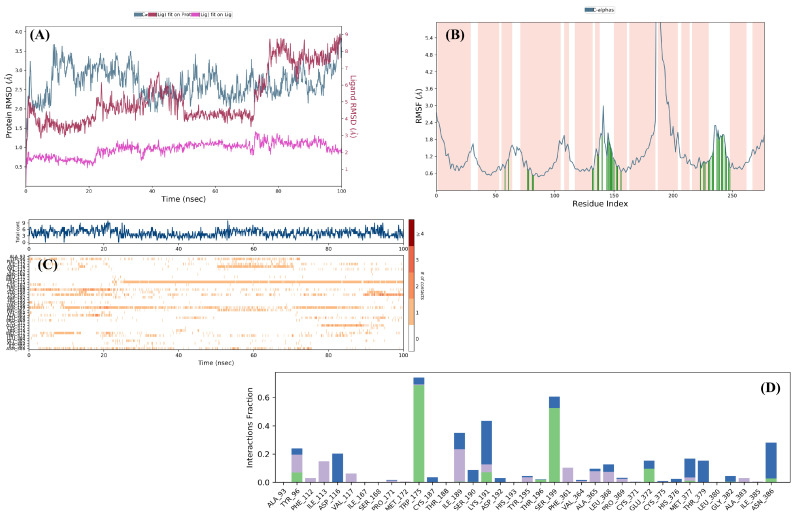
MD simulations results of complex **4e+7EZ2**. (**A**) RMSD profiles of the MD simulations (100 ns) (**B**) RMSF profiles of the MD simulations (100 ns) (**C**) Amino acid interactions timeline graphics of complexes (**D**) Amino acid interactions histogram of complexes

In the final stage of the study, MD simulations were conducted to assess the stability, continuity, and conformational consistency of the interactions between compound **4e** and the 5-HT_1A_ receptor. The RMSD profile obtained from the 100 ns MD simulation of the compound **4e**-7E2Z complex is demonstrated in Figure 13A. The fact that the RMSD value is slightly above the normal limits is clearly seen in video 1, due to the high conformational flexibility and significant oscillatory movements of the methoxy-ethyl side chain. This indicates that the ligand maintains its mobility in peripheral regions without detaching from the active site.

When the RMSF graph (Figure 13) is assessed, the amino acid residues interacting with compound **4e** and their corresponding fluctuation values are determined as follows: Ala93 (0.75 Å), Tyr96 (1.09 Å), Phe112 (0.77 Å), Ile113 (0.69 Å), Asp116 (0.54 Å), Val117 (0.53 Å), Ile167 (0.73 Å), Ser168 (0.79 Å), Pro171 (1.30 Å), Met172 (1.21 Å), Trp175 (1.93 Å), Cys187 (1.16 Å), Thr188 (2.04 Å), Ile189 (1.74 Å), Ser190 (1.57 Å), Lys191 (1.43 Å), Asp192 (1.19 Å), His193 (1.10 Å), Tyr195 (0.87 Å), Thr196 (0.84 Å), Ser199 (0.67 Å), Phe361 (0.98 Å), Val364 (1.03 Å), Ala365 (0.98 Å), Leu368 (1.03 Å), Pro369 (1.08 Å), Cys371 (1.23 Å), Glu372 (1.43 Å), Cys375 (1.85 Å), His376 (2.13 Å), Met377 (1.78 Å), Thr379 (1.89 Å), Leu380 (1.98 Å), Gly382 (1.61 Å), Ala383 (1.15 Å), Ile385 (1.09 Å) and Asn386 (0.78 Å).

Further analysis of the simulation videos revealed several additional interactions of importance. An aromatic hydrogen bond exists between Phe112 and the methoxy group throughout the simulation period. It was observed that the methoxy group formed a hydrogen bond with Tyr96 during the time intervals when this interaction with Phe112 was interrupted. The sulfonyl oxygen atoms of the methylsulfonyl group established hydrogen bonds with Ser199 and Trp175. In addition, the thiadiazole ring formed an aromatic hydrogen bond with Phe361, while the 1,4-disubstituted phenyl ring engaged in an aromatic hydrogen bond with Ser190. A π–π interaction was also detected between the thiadiazole ring and Lys191. The types of these interactions and their time-dependent behavior are presented in Figure 13. Time-dependent interaction analysis further demonstrates that compound **4e** maintained persistent interactions with Ser199 and Ile189 throughout the simulation period, exhibited a continuous interaction with Trp175, and formed particularly strong contacts with Ala116 during the 50–70 ns interval. Collectively, these findings indicate that compound **4e** establishes a stable and dynamically adaptable interaction network within the binding pocket, supporting its proposed partial agonist-like profile at the 5-HT_1A_ receptor.

When molecular modeling and MD simulation findings are evaluated together, it is suggested that the binding behavior of the compounds is supported not only by static docking results but also by interactions occurring in the dynamic process, exhibiting appropriate localization to the active site.

Collectively, these stable ligand–receptor interactions observed in the in silico analyses agree with the in vivo mechanistic findings, which indicate that GABA-A and 5-HT_1A_ receptors play a significant role in mediating the anxiolytic-like effect of compound **4e**. On the other hand, confirming these interactions through radioligand binding assays is essential to fully elucidate the mechanism of action. In addition, the potential roles of other endogenous neuromediator systems (glutaminergic, cholinergic or neuropeptidergic system, etc.) in the anxiolytic-like effect of compound **4e** should also be investigated.

## 3. Materials and Methods

### 3.1. Chemistry

#### 3.1.1. General

In all the synthetic studies described, the progress and completion of reactions were monitored by thin-layer chromatography (TLC). Samples were applied to aluminum plates coated with silica gel 60 F254, which was selected as the adsorbent, via capillary tubes. The plates were run on mobile phases previously saturated with appropriate solvent mixtures. Spots were visualized under ultraviolet light (254 nm and 366 nm). Reactions were terminated or continued based on the TLC results. The most suitable mobile phase for controlling all synthetic steps was determined to be a mixture of petroleum ether: ethyl acetate (3:1, *v*/*v*). The structures of the compounds were determined using ^1^HNMR, ^13^CNMR methods (Supplementary Materials).

Analysis studies were carried out to determine the structure of the compounds. Within the scope of NMR studies, ^1^H-NMR and ^13^C-NMR spectra were taken. DMSO-*d*_6_ was used as solvent. The device is a Bruker brand with a power of 300 MHz. Mass spectra of the compounds were illuminated with LCMS-IT-TOF. High-resolution mass spectra show the purities of the compounds up to four digits after the decimal point.

#### 3.1.2. Synthesis of Target Compounds (**4a**–**4l**)

The synthesis was carried out in four steps.

First step: 1-(4-(Methylsulfonyl)phenyl)ethan-1-one (10 g, 0.05 mol) was dissolved in acetic acid and the resulting reaction mixture was placed in an ice bath with a catalytic amount of HBr. Br2 (3 mL, 0.06 mol) in acetic acid was added to a separating funnel. The Br_2_ solution was added dropwise to the reaction medium. After the dropping was completed, the reaction content was stirred at room temperature for 1 h. After the completion of the reaction was determined by TLC, the reaction mixture was poured into ice water, the crude product was filtered, and crystallized from ethanol.

Second step: Substituted isothiocyanates (4.1 mmol) were dissolved in ethanol. The mixture was placed in an ice bath, and hydrazine hydrate (12.3 mmol, 0.492 mL) in ethanol was added portionwise. After the end of the reaction was determined by TLC, the precipitated product was filtered off and crystallized from ethanol.

Third step: *N*-substituted hydrazinecarbothioamides (4.1 mmol) were dissolved in ethanol. NaOH (4.93 mmol, 0.20 g) was added to the ethanol solution, and CS_2_ (4.93 mmol, 0.30 mL) was added to the medium and refluxed for 4 h. After determining the end of the reaction using TLC, the reaction contents were poured into ice water and acidified with 20% HCl until precipitation was complete (pH ~ 2). The precipitated product was filtered, dried, and crystallized from ethanol.

Fourth step: 1-(4-(Methylsulfonyl)phenyl)ethan-1-one (2 mmol, 0.55 g) was dissolved in acetone. After the appropriate 5-(Substitutedamino)-1,3,4-thiadiazole-2-thiol derivative (2 mmol) was added to the solutions, the reaction was stirred at room temperature using potassium carbonate as a catalyst. After the reaction was continued for 12 h, the reaction was controlled by TLC application. Acetone was evaporated under reduced pressure, and the crude product was washed with water and crystallized from ethanol.

2-((5-(Methylamino)-1,3,4-thiadiazol-2-yl)thio)-1-(4-(methylsulfonyl)phenyl)ethan-1-one (**4a**)

Yield: 79%, M.p.: 172–173 °C. IR (ATR) *ʋ*_max_ (cm^−1^) 3336 (N-H), 1749 (C=O), 844. ^1^H NMR (300 MHz, DMSO-*d*_6_, ppm) δ = 2.85 (3H, d, *J* = 4.8 Hz, -CH_3_), 3.33 (3H, s, -CH_3_), 4.03 (2H, s, -CH_2_-), 7.26 (1H, s, -NH), 7.71–7.74 (2H, m, -phenyl), 7.76–7.79 (2H, m, -phenyl). ^13^C NMR (75 MHz, DMSO-*d*_6_, ppm) δ 31.5, 39.2, 40.9, 119.2, 127.2, 139.2, 142.1, 149.2, 166.9, 171.1. HRMS (*m*/*z*): [M + H]^+^ calcd for C_12_H_13_N_3_O_3_S_3_: 344.0192; found: 344.019

2-((5-(Ethylamino)-1,3,4-thiadiazol-2-yl)thio)-1-(4-(methylsulfonyl)phenyl)ethan-1-one (**4b**)

Yield: 85%, M.p.: 185–186 °C. IR (ATR) *ʋ*_max_ (cm^−1^) 3230 (N-H), 1676 (C=O), 941. ^1^H NMR (300 MHz, DMSO-*d*_6_, ppm) δ = 1.14 (3H, t, *J* = 7.2 Hz, -CH_3_), 3.23–3.27 (2H, m, -CH_2_-), 3.38 (3H, y, -CH_3_), 4.03 (2H, s, -CH_2_-), 7.25 (1H, s, -NH), 7.70–7.73 (2H, m, -phenyl), 7.76–7.79 (2H, m, -phenyl). ^13^C NMR (75 MHz, DMSO-*d*_6_, ppm) δ 14.6, 39.2, 40.0, 40.9, 119.2, 127.2, 139.1, 142.1, 149.1, 166.9, 170.1. HRMS (*m*/*z*): [M + H]^+^ calcd for C_13_H_15_N_3_O_3_S_3_: 358.0348; found: 358.0345.

2-((5-(Propylamino)-1,3,4-thiadiazol-2-yl)thio)-1-(4-(methylsulfonyl)phenyl)ethan-1-one (**4c**)

Yield: 72%, M.p.: 180–181 °C. IR (ATR) *ʋ*_max_ (cm^−1^) 3201 (N-H), 1653 (C=O), 825. ^1^H NMR (300 MHz, DMSO-*d*_6_, ppm) δ = 3.25 (3H, s, -CH_3_), 3.31–3.33 (2H, m, -CH_2_-), 3.40–3.43 (3H, m, -CH_3_), 3.46–3.49 (2H, m, -CH_2_-), 7.26 (1H, br.s., -NH), 7.71–7.74 (2H, m, -phenyl), 7.77–7.79 (2H, m, -CH_2_-). ^13^C NMR (75 MHz, DMSO-*d*_6_, ppm) δ 39.1, 40.9, 44.4, 58.4, 70.4, 119.2, 127.2, 139.2, 142.1, 149.4, 166.9, 170.2. HRMS (*m*/*z*): [M + H]^+^ calcd for C_14_H_17_N_3_O_3_S_3_: 372.0505; found: 372.0510.

2-((5-(Allylamino)-1,3,4-thiadiazol-2-yl)thio)-1-(4-(methylsulfonyl)phenyl)ethan-1-one (**4d**)

Yield: 75%, M.p.: 165–166 °C. IR (ATR) *ʋ*_max_ (cm^−1^) 3277 (N-H), 1654 (C=O), 813. ^1^H NMR (300 MHz, DMSO-*d*_6_, ppm) δ = 3.32 (3H, s, -CH_3_), 3.86–3.90 (2H, m, -CH_2_-), 4.03 (2H, s, -CH_2_-), 5.10–5.25 (2H, m, -CH_2_-), 5.81–5.94 (1H, m, -CH-), 7.25 (1H, br.s., -NH), 7.71–7.74 (2H, m, -phenyl), 7.76–7.79 (2H, m, -phenyl). ^13^C NMR (75 MHz, DMSO-*d*_6_, ppm) δ 39.2, 40.9, 47.1, 116.7, 119.2, 127.2, 134.7, 139.2, 142.1, 149.6, 166.9, 170.2. HRMS (*m*/*z*): [M + H]^+^ calcd for C_14_H_15_N_3_O_3_S_3_: 370.0348; found: 370.0350.

2-((5-((2-Methoxyethyl)amino)-1,3,4-thiadiazol-2-yl)thio)-1-(4-(methylsulfonyl)phen yl)ethan-1-one (**4e**)

Yield: 82%, M.p.: 185–186 °C. IR (ATR) *ʋ*_max_ (cm^−1^) 3255 (N-H), 1654 (C=O), 906. ^1^H NMR (300 MHz, DMSO-*d*_6_, ppm) δ = 0.88 (3H, m, -CH_3_), 1.51–1.57 (2H, m, -CH_2_-), 3.16–3.22 (2H, m, -CH_2_-), 3.32 (3H, s, -CH_3_), 4.03 (2H, s, -CH_2_-), 7.26 (1H, s, -NH), 7.71–7.74 (2H, m, -phenyl), 7.76–7.79 (2H, m, -phenyl). ^13^C NMR (75 MHz, DMSO-*d*_6_, ppm) δ 11.8, 22.2, 39.2, 40.9, 46.8, 119.2, 127.2, 139.1, 142.1, 148.9, 166.9, 170.4. HRMS (*m*/*z*): [M + H]^+^ calcd for C_14_H_17_N_3_O_4_S_3_: 388.0454; found: 388.0454.

2-((5-(Butylamino)-1,3,4-thiadiazol-2-yl)thio)-1-(4-(methylsulfonyl)phenyl)ethan-1-one (**4f**)

Yield: 70%, M.p.: 177–178 °C. IR (ATR) *ʋ*_max_ (cm^−1^) 3240 (N-H), 1654 (C=O), 908. ^1^H NMR (300 MHz, DMSO-*d*_6_, ppm) δ = 0.88 (3H, m, -CH_3_), 1.25–1.38 (2H, m, -CH_2_-), 1.47–1.56 (2H, m, -CH_2_-), 3.19–3.29 (2H, m, -CH_2_-), 3.38 (3H, s, -CH_3_), 4.03 (2H, s, -CH_2_-), 7.25 (1H, br.s., -NH), 7.71–7.75 (2H, m, -phenyl), 7.76–7.79 (2H, m, -phenyl). ^13^C NMR (75 MHz, DMSO-*d*_6_, ppm) δ 14.1, 19.9, 30.9, 39.2, 40.9, 44.7, 119.2, 127.2, 139.1, 142.1, 148.9, 166.9, 170.4. HRMS (*m*/*z*): [M + H]^+^ calcd for C_15_H_19_N_3_O_3_S_3_: 386.0661; found: 386.0665.

2-((5-(Isobutylamino)-1,3,4-thiadiazol-2-yl)thio)-1-(4-(methylsulfonyl)phenyl)ethan-1-one (**4g**)

Yield: 85%, M.p.: 190–191 °C. IR (ATR) *ʋ*_max_ (cm^−1^) 3255 (N-H), 1660(C=O), 817. ^1^H NMR (300 MHz, DMSO-*d*_6_, ppm) δ = 0.88 (6H, d, *J* = 6.7 Hz, -CH_3_), 1.78–1.91 (1H, m, -CH-), 3.04–3.08 (2H, m, -CH_2_-), 3.31 (3H, s, -CH_3_), 4.02 (2H, s, -CH_2_-), 7.26 (1H, br.s., -NH), 7.71–7.74 (2H, m, -phenyl), 7.76–7.79 (2H, m, -phenyl). ^13^C NMR (75 MHz, DMSO-*d*_6_, ppm) δ 20.5, 27.9, 39.2, 40.9, 52.7, 119.2, 127.2, 139.2, 142.1, 148.8, 166.9, 170.6. HRMS (*m*/*z*): [M + H]^+^ calcd for C_15_H_19_N_3_O_3_S_3_: 386.0661; found: 386.0662.

2-((5-(Cyclohexylamino)-1,3,4-thiadiazol-2-yl)thio)-1-(4-(methylsulfonyl)phenyl) ethan-1-one (**4h**)

Yield: 82%, M.p.: 189–190 °C. IR (ATR) *ʋ*_max_ (cm^−1^) 3215 (N-H), 1674 (C=O), 817. ^1^H NMR (300 MHz, DMSO-*d*_6_, ppm) δ = 1.19–1.31 (5H, m, -cyclohexyl), 1.52–1.56 (1H, m, -cyclohexyl), 1.65–1.69 (2H, m, -cyclohexyl), 1.90–1.93 (2H, m, -cyclohexyl), 3.32 (3H, s, -CH_3_), 3.42–3.49 (1H, m, -cyclohexyl), 4.01 (2H, s, -CH_2_-), 7.25 (1H, br.s., -NH), 7.70–7.73 (2H, m, -phenyl), 7.76–7.79 (2H, m, -phenyl). ^13^C NMR (75 MHz, DMSO-*d*_6_, ppm) δ 24.6, 25.7, 32.4, 39.2, 40.9, 53.9, 119.2, 127.2, 139.2, 142.1, 148.7, 166.9, 169.4. HRMS (*m*/*z*): [M + H]^+^ calcd for C_17_H_21_N_3_O_3_S_3_: 412.0818; found: 412.0808.

2-((5-(Phenylamino)-1,3,4-thiadiazol-2-yl)thio)-1-(4-(methylsulfonyl)phenyl)ethan-1-one (**4i**)

Yield: 86%, M.p.: 200–201 °C. IR (ATR) *ʋ*_max_ (cm^−1^) 3226 (N-H), 1656 (C=O), 823. ^1^H NMR (300 MHz, DMSO-*d*_6_, ppm) δ = 3.29 (3H, s, -CH_3_), 4.16 (2H, s, -CH_2_-), 6.97–7.02 (1H, m, -phenyl), 7.30–7.35 (2H, m, -phenyl), 7.54–7.57 (2H, m, -phenyl), 7.57–7.76 (2H, m, -phenyl), 7.76–7.77 (2H, m, -phenyl). ^13^C NMR (75 MHz, DMSO-*d*_6_, ppm) δ 38.9, 40.9, 117.9, 119.2, 122.5, 127.3, 129.6, 139.2, 140.9, 142.1, 165.7, 166.8. HRMS (*m*/*z*): [M + H]^+^ calcd for C_17_H_15_N_3_O_3_S_3_: 406.0348; found: 406.0338.

2-((5-(p-Tolylamino)-1,3,4-thiadiazol-2-yl)thio)-1-(4-(methylsulfonyl)phenyl)ethan-1-one (**4j**)

Yield: 88%, M.p.: 210–211 °C. IR (ATR) *ʋ*_max_ (cm^−1^) 3207 (N-H), 1664 (C=O), 835. ^1^H NMR (300 MHz, DMSO-*d*_6_, ppm) δ = 2.25 (3H, s, -CH_3_), 3.31 (3H, s, -CH_3_), 4.13 (2H, s, -CH_2_-), 7.12 (2H, d, *J* = 8.2 Hz, -phenyl), 7.42 (2H, d, *J* = 8.5 Hz, -phenyl), 7.71–7.74 (2H, m, -phenyl), 7.75–7.79 (2H, m, -phenyl). ^13^C NMR (75 MHz, DMSO-*d*_6_, ppm) δ 20.8, 38.9, 40.9, 118.1, 119.2, 127.3, 129.9, 131.4, 138.7, 139.2, 142.1, 151.6, 165.9, 166.8. HRMS (*m*/*z*): [M + H]^+^ calcd for C_18_H_17_N_3_O_3_S_3_: 420.0505; found: 420.0511.

2-((5-((4-Methoxyphenyl)amino)-1,3,4-thiadiazol-2-yl)thio)-1-(4-(methylsulfonyl) phenyl)ethan-1-one (**4k**)

Yield: 82%, M.p.: 205–206 °C. IR (ATR) *ʋ*_max_ (cm^−1^) 3165 (N-H), 1660 (C=O), 808. ^1^H NMR (300 MHz, DMSO-*d*_6_, ppm) δ = 3.34 (3H, s, -CH_3_), 3.86 (3H, s, -OCH_3_), 4.90 (2H, s, -CH_2_-), 7.08 (2H, d, *J* = 8.9 Hz, -phenyl), 7.37 (2H, d, *J* = 8.9 Hz, -phenyl), 7.59 (2H, d, *J* = 8.9 Hz, -phenyl), 8.03 (2H, d, *J* = 8.9 Hz, -phenyl), 10.51 (1H, s, -NH). ^13^C NMR (75 MHz, DMSO-*d*_6_, ppm) δ 40.9, 41.6, 56.1, 114.5, 119.3, 125.8, 128.5, 129.4, 131.4, 139.7, 153.3, 164.1, 164.9. HRMS (*m*/*z*): [M + H]^+^ calcd for C_18_H_17_N_3_O_4_S_3_: 436.0454; found: 436.0454.

2-((5-((4-Chlorophenyl)amino)-1,3,4-thiadiazol-2-yl)thio)-1-(4-(methylsulfonyl)phen yl)ethan-1-one (**4l**)

Yield: 81%, M.p.: 215–216 °C. IR (ATR) *ʋ*_max_ (cm^−1^) 3215 (N-H), 1664 (C=O), 833. ^1^H NMR (300 MHz, DMSO-*d*_6_, ppm) δ = 3.35 (3H, s, -CH_3_), 4.97 (2H, s, -CH_2_-), 7.35–7.39 (2H, m, -phenyl), 7.57–7.60 (2H, m, -phenyl), 7.67–7.72 (2H, m, -phenyl), 8.03–8.06 (2H, m, -phenyl), 10.51 (1H, s, -NH). ^13^C NMR (75 MHz, DMSO-*d*_6_, ppm) δ 40.8, 41.8, 119.3, 125.8, 128.9, 129.3, 129.4, 134.3, 135.7, 139.7, 153.1, 164.9, 193.6. HRMS (*m*/*z*): [M + H]^+^ calcd for C_17_H_14_N_3_O_3_S_3_Cl: 439.9959; found: 439.9950.

### 3.2. Pharmacology

#### 3.2.1. Animals

Adult male Balb/c mice (30–35 g) of the same age were used for the experimental studies (Total number: 553). To minimize potential confounding effects, animals were housed under identical environmental conditions. Explicitly, they were housed in cages with appropriate bedding and nesting materials, in well-ventilated rooms at 24 ± 1 °C with a 12 h light-dark cycle. The mice were acclimated to the experimental rooms for at least 48 h prior to the experiments. Throughout the study period, mice had ad libitum access to water and rodent chow without competition. All procedures were conducted in a consistent manner across groups.

The study included animals within predefined health (without any experimental modification), age, and weight ranges. Animals showing signs of illness or with abnormal baseline measurement results were excluded from the study. Data values outside a biologically plausible range or those resulting from errors during data collection were not included in the analysis.

The animals were obtained from the Anadolu University Experimental Animals Research and Application Unit. The experimental protocol was approved by the Local Ethical Committee on Animal Experimentation of Anadolu University, Eskişehir, Türkiye. All procedures complied with current legislation of the Republic of Türkiye (Regulation on the Welfare and Protection of Animals Used for Experimental and Other Scientific Purposes, No. 28141; 15 February 2014).

#### 3.2.2. Administration of Test Compounds and Drugs

Mice were randomly assigned to the experimental groups using the online tool QuickCalcs (GraphPad Software, San Diego, CA, USA). The researchers were blinded to group allocations throughout the experimental procedures, outcome assessment, and data analysis.

Test compounds were dissolved in sunflower oil and administered intraperitoneally (i.p.) at a dosage of 30 mg/kg [14,17]. The control group received the same volume of sunflower oil. Fluoxetine (10 mg/kg, i.p.) [35] and reboxetine (20 mg/kg, i.p.) [60] were used as reference drugs in the antidepressant-like effect screening tests while diazepam (1 mg/kg, i.p.) was utilized as a positive control in the assessment of anxiolytic-like effect [26]. Experiments were conducted 30 min following the administrations.

Pharmacological agents used in this study included fluoxetine hydrochloride, reboxetine mesylate hydrate, PCPA, AMPT, flumazenil, and NAN-190 hydrobromide, all of which were procured from Sigma-Aldrich (St. Louis, MO, USA). Diazepam was obtained from Deva Company (Diazem^®^ ampule, Deva, Türkiye).

#### 3.2.3. Evaluation of Antidepressant-like Activity

##### Tail Suspension Test

The tail suspension test was performed in accordance with the method outlined by Steru et al. (1985) [29]. In this test, mice were suspended by their tails (approximately 1 cm from the tip) at a height of 30 cm from the ground using an adhesive patch. The immobility time was measured with a stopwatch during the last 4 min of a 6 min test period. Mice were considered immobile when they hung passively without exhibiting struggling movements [61].

##### Modified Forced Swimming Test

The modified forced swimming experiments were conducted as previously described in the literature [25,32,61]. A glass cylinder (12 cm diameter × 30 cm height) was filled with 20 cm of water maintained at 25 ± 1 °C. Mice were trained in the experimental setup for 15 min 24 h before the test. During the test phase, the immobility, climbing and swimming times of the animals, over a 5 s interval, were recorded using a stopwatch during the 5 min experimental period. Following the training and test phases, the animals were swiftly removed from the water and dried using a light source.

#### 3.2.4. Evaluation of Anxiolytic-like Activity

##### Hole Board Test

Exploratory behavior of animals was assessed using a hole board device (Ugo-basile, 6650, Varese, Italy). This device features 16 equivalent holes (3 cm diameter) on a gray Plexiglas panel (40 × 40 cm) elevated 15 cm above the ground. During the experiment, animals were placed one by one in the center of the device, facing away from the researcher and latencies to the first head-dip, total number of head-dips, and total number of explored holes were recorded for 5 min [35,62].

##### Elevated Plus Maze Test

Anxiety levels of animals were assessed using the elevated plus maze apparatus (Ugo Basile, 40143, Varese, Italy) [25,26]. The apparatus consists of two open arms (35 cm × 5 cm), two closed arms (35 cm × 5 cm × 15 cm), and a central area which connects the open and closed arms. The apparatus is 60 cm above the floor. In the experiments, the number of entries and time spent in the open versus closed arms of animals were recorded over a 5 min period.

POAE% and PTOA% for each animal were calculated as follows:

$$\mathrm{POAE}\%\;=\;\frac{\mathrm{Number}\;\mathrm{of}\;\mathrm{the}\;\mathrm{open}\;\mathrm{arm}\;\mathrm{entries}}{\mathrm{Number}\;\mathrm{of}\;\mathrm{the}\;\mathrm{open}\;\mathrm{and}\;\mathrm{closed}\;\mathrm{arms}\;\mathrm{entries}}\;\times \;100$$

$$\mathrm{PTOA}\%=\frac{\mathrm{Time}\;\mathrm{spent}\;\mathrm{in}\;\mathrm{open}\;\mathrm{arms}}{\mathrm{Time}\;\mathrm{spent}\;\mathrm{in}\;\mathrm{open}\;\mathrm{and}\;\mathrm{closed}\;\mathrm{arms}}\;\times \;100$$

##### Open Field Test

The open field test was conducted using an acrylic apparatus featuring transparent walls and a black floor, measuring 41 cm × 41 cm × 33 cm. The floor of the apparatus was virtually partitioned into central and peripheral areas [63]. At the beginning of the test, each mouse was placed in the center of the floor and allowed to explore a period of 5 min. Throughout this time, the time each mouse spent in the central area was recorded. Each mouse was tested only once, and the apparatus was thoroughly cleaned with ethanol after each trial to prevent any potential residue [27].

#### 3.2.5. Evaluation of Motor Activity

##### Activity-Meter Test

The motor activity of the animals was assessed using an activity-meter device (Commat, MayAMS02, Ankara, Türkiye). Each mouse was positioned in the apparatus’s center, and total activity counts, number of ambulatory activities, and walking distance, were recorded over a 5 min period [38]. The apparatus was cleaned with ethanol after each use.

#### 3.2.6. Mechanistic Studies

To investigate the roles of the serotonergic and catecholaminergic systems in the antidepressant-like effects of the test compounds, mechanistic studies were performed using PCPA (serotonin synthesis inhibitor) and AMPT (catecholamine synthesis inhibitor), respectively.

In studies conducted with PCPA, mice received an intraperitoneal injection of PCPA at a dosage of 100 mg/kg for four consecutive days and the test compounds were administered 24 h after the last PCPA injection. Tail suspension tests were performed 30 min after the administrations of the test compounds and the control solution [64]. In the AMPT studies, mice were injected intraperitoneally with AMPT at a dose of 100 mg/kg 4 h prior to the administrations of the test compounds and control solution, and the tail suspension test conducted 30 min after these administrations [25,64].

Antagonism studies were carried out using flumazenil (6 mg/kg), a benzodiazepine receptor antagonist [45], and NAN-190 (0.5 mg/kg), a selective 5-HT_1A_ receptor antagonist [46], to investigate the mechanisms underlying the anxiolytic-like activity. The antagonists were administered intraperitoneally 15 min prior to the administrations of the test compound and control solution.

#### 3.2.7. Statistical Analysis

Statistical analyses and graph illustrations were performed using GraphPad Prism version 8.4.3. The sample size was determined based on the “numbers of animals” used in similar studies in the literature and our previous experiences in the laboratory. All data were tested for normality distribution using Shapiro–Wilk test. Data obtained from experiments (*n* = 7 in each group) were analyzed using one-way analysis of variance (ANOVA) followed by Tukey-HSD multiple comparison tests. Results are expressed as mean ± standard error of the mean, with *p* < 0.05 considered significant.

### 3.3. Computational In Silico Studies

#### 3.3.1. Molecular Docking

Molecular docking studies were performed using an in silico approach to elucidate the binding modes of the active compounds within the active sites of 5-HT_1A_ and GABA-A receptors, which are key targets involved in CNS disorders. The selection of these proteins was based on their well-established roles in neurotransmission and their pharmacological relevance in the treatment of neuropsychiatric conditions such as anxiety and depression. In addition, to further support the mechanistic interpretation and provide a broader perspective on monoaminergic signaling, the SERT and DAT were also included in the study, as they play crucial roles in serotonin and dopamine reuptake processes, respectively.

The X-ray crystal structures of SERT (PDB ID: 7LWD) [65], DAT (PDB ID: 4XNX) [66] GABA-A (PDB ID: 6HUO) [67], and 5HT_1A_ (PDB ID: 7E2Z) [68], and were retrieved from the Protein Data Bank (https://www.wwpdb.org/) (Accessed on 1 July 2025). Protein structures were prepared using the Schrödinger Maestro interface [69] and subsequently processed with the Protein Preparation Wizard protocol of Schrödinger Suite 2020. Ligands were prepared using the LigPrep module [70] to assign appropriate protonation states and atom types. Grid generation was performed using the Glide module [71], and docking simulations were carried out using the standard precision (SP) docking mode.

#### 3.3.2. Molecular Dynamics Simulations

MD simulations use groups of the Schrödinger Suite to determine the stability of selected samples with the target enzyme and the connectivity of the linkages. The detailed protocol and steps taken for simulations using Desmond (Schrödinger’s MD module) and Maestro (Schrödinger’s graphical user interface) have been previously reported by our system [72,73,74,75,76,77,78].

## 4. Conclusions

In conclusion, in this study, antidepressant-like and anxiolytic-like effects of some novel 1,3,4-thiadiazole derivatives were examined using various in vivo methods. The obtained results indicated that compounds **4f**, **4g**, **4h** and **4i** exhibit antidepressant-like effects whereas compound **4e** displays both antidepressant-like and anxiolytic-like effects. Mechanistic studies pointed out that the monoaminergic system plays a role in the antidepressant-like effects of compounds **4e**, **4f**, **4g**, **4h** and **4i** while 5-HT_1A_ receptors and GABA(A)/benzodiazepine receptor complex participate in the anxiolytic-like effect of compound **4e**. In silico studies conducted following the in vivo experiments revealed that compounds **4e**–**4i** interact with the SERT but not the DAT; additionally, compound **4e** displayed affinity for GABA-A and 5-HT_1A_ receptors. Furthermore, MD simulations indicated that these interactions are stable throughout dynamic conditions. According to MD simulation results, compound **4e** exhibited RMSD values of 3.2 Å, 2.7 Å, and 4.0 Å in its complexes with SERT (7LWD), GABA-A receptor (6HUO), and 5-HT_1A_ receptor (7E2Z), respectively. These results indicate that the compound forms a more stable complex with the GABA-A receptor, exhibits moderate stability with SERT, and shows relatively higher conformational fluctuations with the 5-HT_1A_ receptor. The findings suggest that compound **4e** has the potential to interact with multiple targets but exhibits different target-dependent stability profiles.

When the ADME estimation results obtained in this study were examined, it was observed that the molecular weights of the synthesized compounds ranged from 343.433 to 439.949, and their PSA values ranged from 100.095 to 114.421 Å^2^. When the solubility values (QPlogS: −3.618 to −5.717) and blood–brain barrier crossing potentials (QPlogBB: −1.284 to −1.922) of the compounds were evaluated, it was understood that the compounds exhibited a limited but improvable profile in terms of CNS penetration. Furthermore, the fact that none of the compounds showed a violation of the Lipinski Rule indicates that these derivatives offer a suitable profile in terms of drug-like properties.

This study supports the previous papers reporting the antidepressant-like and anxiolytic-like activities of 1,3,4-thiadiazole derivatives [12,13,14,15,16,17,18,19]. In addition, it provides new insights into the mechanisms underlying the pharmacological effects of compounds containing the 1,3,4-thiadiazole ring. Nonetheless, comprehensive preclinical and clinical studies are necessary to validate the antidepressant and anxiolytic efficacy of these compounds.

## Figures and Tables

**Table 1:** Predicted ADME parameters of compounds **4a**–**4l**.

Comp.	Mol MW *	Volume *	DHB *	AHB *	QPlogS *	QPlogBB *	PHOA *	PSA *	Rule of Five *	Rule of Three *
**4a**	343.433	1016.943	1	8.5	−3.618	−1.456	75.130	104.710	0	0
**4b**	357.460	1077.289	1	8.5	−3.935	−1.674	75.575	106.041	0	0
**4c**	371.487	1126.996	1	8.5	−4.199	−1.427	83.489	101.076	0	0
**4d**	369.471	1115.709	1	8.5	−4.070	−1.730	77.640	106.111	0	0
**4e**	387.486	1158.427	1	10.2	−3.681	−1.848	74.536	113.249	0	0
**4f**	385.513	1197.622	1	8.5	−4.713	−1.922	79.618	105.716	0	0
**4g**	385.513	1168.739	1	8.5	−4.322	−1.588	81.963	102.892	0	0
**4h**	411.551	1247.661	1	8.5	−5.402	−1.284	90.340	100.095	0	0
**4i**	405.504	1192.329	1	8.5	−4.987	−1.477	86.007	100.986	0	0
**4j**	419.531	1253.734	1	8.5	−5.565	−1.536	87.676	101.030	0	0
**4k**	435.530	1260.353	1	9.25	−4.814	−1.905	79.327	114.421	0	0
**4l**	439.949	1236.563	1	8.5	−5.717	−1.338	88.870	100.949	0	1

* Mol MW, molecular weight (Da); DHB, number of hydrogen bond donors; AHB, number of hydrogen bond acceptors; QPlogS, predicted aqueous solubility (log S); QPlogBB, predicted brain/blood partition coefficient (log BB); PHOA, predicted human oral absorption (%); PSA, polar surface area (Å^2^); rule of five, number of Lipinski’s rule of five violations; rule of three, number of rules of three violations.

**Table 2:** Molecular docking scores (kcal/mol) of compounds **4a**–**4l** against target proteins (7LWD, 6HUO, 7E2Z, and 4XNX).

Comp.	PDB ID: 7LWD	PDB ID: 7EZ2	PDB ID: 6HUO	PDB ID: 4XNX
**4a**	−6.321	−5.845	−7.852	−6.171
**4b**	−6.760	−5.236	−8.118	−5.773
**4c**	−6.525	−7.165	−8.253	−5.567
**4d**	−6.817	−6.133	−7.834	−5.253
**4e**	−6.721	−5.883	−8.202	−5.133
**4f**	−6.729	−5.980	−8.182	−5.780
**4g**	−6.850	−6.400	−7.387	−5.377
**4h**	−5.999	−6.133	−6.317	−6.313
**4i**	−7.401	−6.890	−8.184	−5.266
**4j**	−7.114	−6.301	−7.474	−5.504
**4k**	−7.025	−5.929	−7.622	−5.340
**4l**	−7.709	−6.306	−7.777	−5.511

## Data Availability

All relevant data are included within the article or Supplementary Materials. The raw data are available upon request from the corresponding author.

## Supplementary Materials

The following supporting information can be downloaded at: https://www.mdpi.com/article/10.3390/ph19050797/s1. ^1^H-NMR, ^13^C-NMR and HRMS spectrums of compounds **4a**–**4l** and 2D representations of the interactions of compounds **4e**, **4f**, **4g**, **4h** and **4i** with the active site of the DAT and 2D representations of the interactions of vilazodone, alprazolam and aripiprazole with the active site of the SERT, GABA, 5HT_1A_ are available in Supplementary Materials. Figure S1. Chemical structure and spectral data of compound **4a**. Figure S2. Chemical structure and spectral data of compound **4b**. Figure S3. Chemical structure and spectral data of compound **4c**. Figure S4. Chemical structure and spectral data of compound **4d**. Figure S5. Chemical structure and spectral data of compound **4e**. Figure S6. Chemical structure and spectral data of compound **4f**. Figure S7. Chemical structure and spectral data of compound **4g**. Figure S8. Chemical structure and spectral data of compound **4h**. Figure S9. Chemical structure and spectral data of compound **4i**. Figure S10. Chemical structure and spectral data of compound **4j**. Figure S11. Chemical structure and spectral data of compound **4k**. Figure S12. Chemical structure and spectral data of compound **4l**. Figure S13. HRMS spectrum of compound **4a**. Figure S14. HRMS spectrum of compound **4b**. Figure S15. HRMS spectrum of compound **4c**. Figure S16. HRMS spectrum of compound **4d**. Figure S17. HRMS spectrum of compound **4e**. Figure S18. HRMS spectrum of compound **4f**. Figure S19. HRMS spectrum of compound **4g**. Figure S20. HRMS spectrum of compound **4h**. Figure S21. HRMS spectrum of compound **4i**. Figure S22. HRMS spectrum of compound **4j**. Figure S23. HRMS spectrum of compound **4k**. Figure S24. HRMS spectrum of compound **4l**. Figure S25. Two-dimensional (2D) representation of the interactions of compounds **4e**, **4f** and **4g** with the active site of the DAT (PDB ID: 4XNX). Figure S26. Two-dimensional (2D) representation of the interactions of compounds **4h** and **4i** with the active site of the DAT (PDB ID: 4XNX). Figure S27. Two-dimensional (2D) representation of the interactions of vilazadone with the active site of the SERT (PDB ID: 7LWD). Figure S28. Two-dimensional (2D) representation of the interactions of alprazolam with the active site of the GABA-A receptor (PDB ID: 6HUO). Figure S29. Two-dimensional (2D) representation of the interactions of aripiprazole with the active site of the 5HT_1A_ receptor (PDB ID: 7E2Z).

## Abbreviations

The following abbreviations are used in this manuscript:

PCPA	*p*-chlorophenylalanine methyl ester
AMPT	α-methyl-para-tyrosine methyl ester
ANOVA	Analysis of variance
CNS	Central nervous system
DAT	Dopamine transporter
HSD	Honestly significant difference
i.p.	Intraperitoneally
MD	Molecular dynamics
NET	Norepinephrine transporter
POAE%	Percentage of open arm entries
PTOA%	Percentage of time spent in open arms
SERT	Serotonin transporter
SP	Standard precision
TLC	Thin-layer chromatography

## References

[1] WHO Mental Disorders. https://www.who.int/news-room/fact-sheets/detail/mental-disorders.

[2] Nallapu S., Ghonge S., Johnson S., Vajjala S.M., Palal D. (2023). Impact of COVID-19 pandemic on mental health of general population: A comparison study between rural and urban population. Ind. Psychiatry J..

[3] Tokgöz G., Demir Özkay Ü., Osmaniye D., Turan Yücel N., Can Ö.D., Kaplancıklı Z.A. (2018). Synthesis of novel benzazole derivatives and evaluation of their antidepressant-like activities with possible underlying mechanisms. Molecules.

[4] Żmudzka E., Lustyk K., Głuch-Lutwin M., Wolak M., Jaśkowska J., Kołaczkowski M., Sapa J., Pytka K. (2023). Novel multimodal salicylamide derivative with antidepressant-like, anxiolytic-like, antipsychotic-like, and anti-amnesic activity in mice. Pharmaceuticals.

[5] Haider S., Alam M.S., Hamid H. (2015). 1,3,4-Thiadiazoles: A potent multi targeted pharmacological scaffold. Eur. J. Med. Chem..

[6] Gomha S.M., Edrees M.M., Muhammad Z.A., El-Reedy A.A. (2018). 5-(Thiophen-2-yl)-1,3,4-thiadiazole derivatives: Synthesis, molecular docking and in vitro cytotoxicity evaluation as potential anticancer agents. Drug Des. Dev. Ther..

[7] Madhu Sekhar M., Nagarjuna U., Padmavathi V., Padmaja A., Reddy N.V., Vijaya T. (2018). Synthesis and antimicrobial activity of pyrimidinyl 1,3,4-oxadiazoles, 1,3,4-thiadiazoles and 1,2,4-triazoles. Eur. J. Med. Chem..

[8] Serban G. (2020). Synthetic compounds with 2-amino-1,3,4-thiadiazole moiety against viral infections. Molecules.

[9] Bekhit A.A., Hassan A.M., Abd El Razik H.A., El-Miligy M.M., El-Agroudy E.J., Bekhit A.-D. (2015). New heterocyclic hybrids of pyrazole and its bioisosteres: Design, synthesis and biological evaluation as dual acting antimalarial-antileishmanial agents. Eur. J. Med. Chem..

[10] Salgin-Gökşen U., Gökhan-Kelekçi N., Göktaş O., Köysal Y., Kiliç E., Işik S., Aktay G., Ozalp M. (2007). 1-Acylthiosemicarbazides, 1,2,4-triazole-5(4H)-thiones, 1,3,4-thiadiazoles and hydrazones containing 5-methyl-2-benzoxazolinones: Synthesis, analgesic-anti-inflammatory and antimicrobial activities. Bioorg. Med. Chem..

[11] Pandey A., Rajavel R., Chandraker S., Dash D. (2012). Synthesis of schiff bases of 2-amino-5-aryl-1,3,4-thiadiazole and its analgesic, anti-inflammatory and anti-bacterial activity. J. Chem..

[12] Yusuf M.M., Khan R.A., Ahmed B. (2008). Syntheses and anti-depressant activity of 5-amino-1,3,4-thiadiazole-2-thiol imines and thiobenzyl derivatives. Bioorg. Med. Chem..

[13] Pattanayak P., Sharma R., Sahoo P.K. (2009). Synthesis and evaluation of 2-amino-5-sulfanyl-1,3,4-thiadiazoles as antidepressant, anxiolytic, and anticonvulsant agents. Med. Chem. Res..

[14] Sharma R., Misra G.P., Sainy J., Chaturvedi S.C. (2011). Synthesis and biological evaluation of 2-amino-5-sulfanyl-1,3,4-thiadiazole derivatives as antidepressant, anxiolytics and anticonvulsant agents. Med. Chem. Res..

[15] Sharma R., Prasad Y., Mishra G.P., Chaturvedi S.C. (2014). Some substituted 1,3,4-thiadiazoles: A novel centrally acting agents. Med. Chem. Res..

[16] Can O.D., Altintop M.D., Ozkay U.D., Uçel U.I., Doğruer B., Kaplancikli Z.A. (2012). Synthesis of thiadiazole derivatives bearing hydrazone moieties and evaluation of their pharmacological effects on anxiety, depression, and nociception parameters in mice. Arch. Pharm. Res..

[17] Can N.O., Can O.D., Osmaniye D., Ozkay U.D. (2018). Synthesis of some novel thiadiazole derivative compounds and screening their antidepressant-like activities. Molecules.

[18] Clerici F., Pocar D., Guido M., Loche A., Perlini V., Brufani M. (2001). Synthesis of 2-amino-5-sulfanyl-1,3,4-thiadiazole derivatives and evaluation of their antidepressant and anxiolytic activity. J. Med. Chem..

[19] Singh V.K., Bharadwaj P., Rishishwar P. (2018). Synthesis and anxiolytic activity of 2-(substituted)-5-[(N-benzotriazolomethyl)-1,3,4-thiadiazolyl]-4-thiazolidinone. Drug Des. Int. Prop. Int. J..

[20] Altıntop M.D., Can M.D., Demir Özkay Ü., Kaplancıklı Z.A. (2016). Synthesis and evaluation of new 1,3,4-thiadiazole derivatives as antinociceptive agents. Molecules.

[21] O’Neill D.J., Adedoyin A., Bray J.A., Deecher D.C., Fensome A., Goldberg J.A., Harrison J., Leventhal L., Mann C., Mark L. (2011). Discovery of novel selective norepinephrine inhibitors: 1-(2-morpholin-2-ylethyl)-3-aryl-1,3-dihydro-2,1,3-benzothiadiazole 2,2-dioxides (WYE-114152). J. Med. Chem..

[22] Sabb A.L., Vogel R.L., Kelly M.G., Palmer Y., Smith D.L., Andree T.H., Schecter L.E. (2001). 1,2,5-Thiadiazole derivatives are potent and selective ligands at human 5-HT1A receptors. Bioorg. Med. Chem. Lett..

[23] Karaküçük-İyidoğan A., Başaran E., Tatar-Yılmaz G., Oruç-Emre E.E. (2024). Development of new chiral 1,2,4-triazole-3-thiones and 1,3,4-thiadiazoles with promising in vivo anticonvulsant activity targeting GABAergic system and voltage-gated sodium channels (VGSCs). Bioorg. Chem..

[24] Can O.D., Turan N., Ozkay U.D., Öztürk Y. (2017). Antidepressant-like effect of gallic acid in mice: Dual involvement of serotonergic and catecholaminergic systems. Life Sci..

[25] Can O.D., Ozkay U.D., Üçel U.İ. (2013). Anti-depressant-like effect of vitexin in BALB/c mice and evidence for the involvement of monoaminergic mechanisms. Eur. J. Pharmacol..

[26] Can O.D., Turan N., Alyu F. (2016). Benzodiazepine receptors mediated anxiolytic-like effects of some 1,3,5-triaryl-4,5-dihydro-1h-pyrazole derivatives. Cukurova Med. J..

[27] Can O.D., Ozkay U.D., Kıyan H.T., Demirci B. (2012). Psychopharmacological profile of Chamomile (*Matricaria recutita* L.) essential oil in mice. Phytomedicine.

[28] Prut L., Belzung C. (2003). The open field as a paradigm to measure the effects of drugs on anxiety-like behaviors: A review. Eur. J. Pharmacol..

[29] Steru L., Chermat R., Thierry B., Simon P. (1985). The tail suspension test: A new method for screening antidepressants in mice. Psychopharmacology.

[30] Cryan J.F., Mombereau C., Vassout A. (2005). The tail suspension test as a model for assessing antidepressant activity: Review of pharmacological and genetic studies in mice. Neurosci. Biobehav. Rev..

[31] Oliveira C.E., Sari M.H., Zborowski V.A., Araujo P.C., Nogueira C.W., Zeni G. (2017). p,p′-Methoxyl-diphenyl diselenide elicits an antidepressant-like effect in mice without discontinuation anxiety phenotype. Pharmacol. Biochem. Behav..

[32] Cryan J.F., Markou A., Lucki I. (2002). Assessing antidepressant activity in rodents: Recent developments and future needs. Trends Pharmacol. Sci..

[33] Detke M.J., Rickels M., Lucki I. (1995). Active behaviors in the rat forced swimming test differentially produced by serotonergic and noradrenergic antidepressants. Psychopharmacology.

[34] Slattery D.A., Cryan J.F. (2012). Using the rat forced swim test to assess antidepressant-like activity in rodents. Nat. Protoc..

[35] Can O.D., Ozkay U.D., Kaplancikli Z.A., Oztürk Y. (2009). Effects of some 1,3,5-trisubstitued-2-pyrazoline derivatives on depression and anxiety parameters of mice. Arch. Pharm. Res..

[36] File S.E., Pellow S. (1985). The effects of triazolobenzodiazepines in two animal tests of anxiety and in the holeboard. Br. J. Pharmacol..

[37] Sampath C., Holbik M., Krenn L., Butterweck V. (2011). Anxiolytic effects of fractions obtained from *Passiflora incarnata* L. in the elevated plus maze in mice. Phytother. Res..

[38] Güzelad Ö., Özkan A., Parlak H., Sinen O., Afşar E., Öğüt E., Yıldırım F.B., Bülbül M., Ağar A., Aslan M. (2021). Protective mechanism of Syringic acid in an experimental model of Parkinson’s disease. Metab. Brain Dis..

[39] Perez-Caballero L., Torres-Sanchez S., Romero-López-Alberca C., González-Saiz F., Mico J.A., Berrocoso E. (2019). Monoaminergic system and depression. Cell Tissue Res..

[40] Koe B.K., Weissman A. (1966). *p*-Chlorophenylalanine: A specific depletor of brain serotonin. J. Pharmacol. Exp. Ther..

[41] Redrobe J.P., Bourin M., Colombel M.C., Baker G.B. (1998). Dose-dependent noradrenergic and serotonergic properties of venlafaxine in animal models indicative of antidepressant activity. Psychopharmacology.

[42] Redrobe J.P., Bourin M., Colombel M.C., Baker G.B. (1998). Psychopharmacological profile of the selective serotonin reuptake inhibitor, paroxetine: Implication of noradrenergic and serotonergic mechanisms. J. Psychopharmacol..

[43] Widerlöv E., Lewander T. (1978). Inhibition of the in vivo biosynthesis and changes of catecholamine levels in rat brain after alpha-methyl-p-tyrosine; time- and dose-response relationships. Naunyn-Schmiedeberg’s Arch. Pharmacol..

[44] Mayorga A.J., Dalvi A., Page M.E., Zimov-Levinson S., Hen R., Lucki I. (2001). Antidepressant-like behavioral effects in 5-hydroxytryptamine(1A) and 5-hydroxytryptamine(1B) receptor mutant mice. J. Pharmacol. Exp. Ther..

[45] Narasingam M., Vijeepallam K., Mohamed Z., Pandy V. (2017). Anxiolytic- and antidepressant-like activities of a methanolic extract of *Morinda citrifolia* Linn. (noni) fruit in mice: Involvement of benzodiazepine-GABA_A_ergic, serotonergic and adrenergic systems. Biomed. Pharmacother..

[46] Gabriel de Oliveira M., Kelle da Silva Moreira L., Turones L.C., de Souza Almeida D., Martins A.N., Silva Oliveira T.L., Barreto da Silva V., Borges L.L., Costa E.A., Realino de Paula J. (2021). Mechanism of action involved in the anxiolytic-like effects of Hibalactone isolated from *Hydrocotyle umbellata* L.. J. Tradit. Complement. Med..

[47] Nuss P. (2015). Anxiety disorders and GABA neurotransmission: A disturbance of modulation. Neuropsychiatr. Dis. Treat..

[48] Griffin C.E., Kaye A.M., Bueno F.R., Kaye A.D. (2013). Benzodiazepine pharmacology and central nervous system-mediated effects. Ochsner J..

[49] Almeida L.S., Santana I.G.C., da Silva Moreira L.K., Turones L.C., Sanz G., Vaz B.G., de Carvalho F.S.L.M., Lião R., Menegatti E.A., de Brito C.A.F. (2022). Neuropharmacological activity of the new piperazine derivative 2-(4-((1-phenyl-1h-pyrazol-4-yl)methyl)piperazin-1-yl)ethyl acetate is modulated by serotonergic and GABAergic pathways. CNS Neurol. Disord. Drug Targets.

[50] Blier P., Lista A., De Montigny C. (1993). Differential properties of pre- and postsynaptic 5-hydroxytryptamine1A receptors in the dorsal raphe and hippocampus: I. Effect of spiperone. J. Pharmacol. Exp. Ther..

[51] Liu J., Zhai W.M., Yang Y.X., Shi J.L., Liu Q.T., Liu G.L., Fang N., Li J., Guo J.Y. (2015). GABA and 5-HT systems are implicated in the anxiolytic-like effect of spinosin in mice. Pharmacol. Biochem. Behav..

[52] Shanmugasundaram J., Subramanian V., Nadipelly J., Kathirvelu P., Sayeli V., Cheriyan B.V. (2020). Anxiolytic-like activity of 5-methoxyflavone in mice with involvement of GABAergic and serotonergic systems—In vivo and in silico evidences. Eur. Neuropsychopharmacol..

[53] Ravindran L.N., Stein M.B. (2010). The pharmacologic treatment of anxiety disorders: A review of progress. J. Clin. Psychiatry.

[54] Cui L., Li S., Wang S., Wu X., Liu Y., Yu W., Wang Y., Tang Y., Xia M., Li B. (2024). Major depressive disorder: Hypothesis, mechanism, prevention and treatment. Signal Transduct. Target. Ther..

[55] Dashyan S.S., Babaev E.V., Paronikyan E.G., Ayvazyan A.G., Paronikyan R.G., Hunanyan L.S. (2022). Evaluation of neurotropic activity and molecular docking study of new derivatives of pyrano[4″,3″:4′,5′]pyrido[3′,2′:4,5]thieno[3,2-d]pyrimidines on the basis of pyrano[3,4-c]pyridines. Molecules.

[56] Wu Y., Cai J., Liu H., Li C., Tang Q., Zhang Y.W. (2024). (-)-syringaresinol exerts an antidepressant-like activity in mice by noncompetitive inhibition of the serotonin transporter. Pharmaceuticals.

[57] Penmatsa A., Wang K.H., Gouaux E. (2013). X-ray structure of dopamine transporter elucidates antidepressant mechanism. Nature.

[58] Beuming T., Kniazeff J., Bergmann M.L., Shi L., Gracia L., Raniszewska K., Newman A.H., Javitch J.A., Weinstein H., Gether U. (2008). The binding sites for cocaine and dopamine in the dopamine transporter overlap. Nat. Neurosci..

[59] Pytka K., Podkowa K., Rapacz A., Podkowa A., Żmudzka E., Olczyk A., Sapa J., Filipek B. (2016). The role of serotonergic, adrenergic and dopaminergic receptors in antidepressant-like effect. Pharmacol. Rep..

[60] Can O.D., Ismail I.B., Oztürk Y., Oztürk N., Potoğlu-Erkara I., Sagratini G., Ricciutelli M., Vittori S., Maggi F. (2011). New antidepressant drug candidate: *Hypericum montbretti* extract. Nat. Prod. Res..

[61] Kaya C., Turan-Yücel N., Kandemir Ü., Osmaniye D., Can Ö.D., Demir Özkay Ü. (2022). Synthesis and antidepressant-like activities of some piperidine derivatives: Involvements of monoaminergic and opioidergic systems. Acta Pol. Pharm. Drug Res..

[62] Takeda H., Tsuji M., Matsumiya T. (1998). Changes in head-dipping behavior in the hole-board test reflect the anxiogenic and/or anxiolytic state in mice. Eur. J. Pharmacol..

[63] Panlilio L.V., Solinas M., Matthews S.A., Goldberg S.R. (2007). Previous exposure to THC alters the reinforcing efficacy and anxiety-related effects of cocaine in rats. Neuropsychopharmacology.

[64] Kwon S., Lee B., Kim M., Lee H., Park H.J., Hahm D.H. (2010). Antidepressant-like effect of the methanolic extract from *Bupleurum falcatum* in the tail suspension test. Prog. Neuropsychopharmacol. Biol. Psychiatry.

[65] Plenge P., Yang D., Salomon K., Laursen L., Kalenderoglou I.E., Newman A.H., Gouaux E., Coleman J.A., Loland C.J. (2021). The antidepressant drug vilazodone is an allosteric inhibitor of the serotonin transporter. Nat. Commun..

[66] Penmatsa A., Wang K.H., Gouaux E. (2015). X-ray structures of Drosophila dopamine transporter in complex with nisoxetine and reboxetine. Nat. Struct. Mol. Biol..

[67] Masiulis S., Desai R., Uchański T., Serna Martin I., Laverty D., Karia D., Malinauskas T., Zivanov J., Pardon E., Kotecha A. (2019). GABA_A_ receptor signalling mechanisms revealed by structural pharmacology. Nature.

[68] Xu P., Huang S., Zhang H., Mao C., Zhou X.E., Cheng X., Simon I.A., Shen D.D., Yen H.Y., Robinson C.V. (2021). Structural insights into the lipid and ligand regulation of serotonin receptors. Nature.

[69] Schrödinger, LLC (2020). Maestro.

[70] Schrödinger, LLC (2020). LigPrep.

[71] Schrödinger, LLC (2020). Glide.

[72] Liu X., Shi D., Zhou S., Liu H., Liu H., Yao X. (2018). Molecular dynamics simulations and novel drug discovery. Expert Opin. Drug Discov..

[73] Tools M.D.I. (2020). Schrödinger Release 2018-3: Prime, 2018.

[74] Sureshkumar B., Mary Y.S., Resmi K.S., Suma S., Armaković S., Armaković S.J., Van Alsenoy C., Narayana B., Sobhana D. (2018). Spectroscopic characterization of hydroxyquinoline derivatives with bromine and iodine atoms and theoretical investigation by DFT calculations, MD simulations and molecular docking studies. J. Mol. Struct..

[75] Humphreys D.D., Friesner R.A., Berne B.J. (1994). A Multiple-Time-Step Molecular dynamics algorithm for macromolecules. J. Phys. Chem..

[76] Hoover W.G. (1985). Canonical dynamics: Equilibrium phase-space distributions. Phys. Rev. A.

[77] Martyna G.J., Tobias D.J., Klein M.L. (1994). Constant pressure molecular dynamics algorithms. J. Chem. Phys..

[78] Essmann U., Perera L., Berkowitz M.L., Darden T., Lee H., Pedersen L.G. (1995). A smooth particle mesh Ewald method. J. Chem. Phys..

