# Supplementary material for: Synthesis, Antidepressant-like and Anxiolytic-like Effects of Novel Thiadiazole Derivatives: Behavioral Assessment and Mechanistic Investigation
Source: Pharmaceuticals (Basel). 2026 May 19;19(5):797. doi: 10.3390/ph19050797 (PMC13210115; doi:10.3390/ph19050797)

SYNTHESIS, ANTIDEPRESSANT-LIKE and ANXIOLYTIC-LIKE EFFECTS of NOVEL THIADIAZOLE DERIVATIVES: BEHAVIORAL ASSESSMENT and MECHANISTIC INVESTIGATION

Ümmühan Kandemir[1], Gizem Türkoğlu Sağlik[2], Derya Osmaniye[3,4], Zafer Asım Kaplancikli[3], Özgür Devrim Can[2], Ümide Demir Özkay[2,*]

[1] Department of Medical Pharmacology, Faculty of Medicine, Bilecik Şeyh Edebali University, 11100, Bilecik, Türkiye

[2] Department of Pharmacology, Faculty of Pharmacy, Anadolu University, 26470, Eskişehir, Türkiye

[3] Department of Pharmaceutical Chemistry, Faculty of Pharmacy, Anadolu University, 26470, Eskişehir, Türkiye

[4] Central Research Laboratory, Faculty of Pharmacy, Anadolu University, 26470, Eskişehir, Türkiye

***Corresponding author:**

Ümide DEMİR ÖZKAY

Anadolu University, Faculty of Pharmacy,

Department of Pharmacology, 26470 Eskişehir, TÜRKİYE

e-mail address: udemir@anadolu.edu.tr

Figure S1. Chemical structure and spectral data of compound **4a**

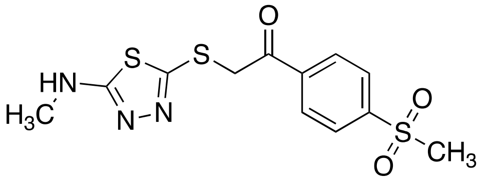

DOPNALAB

Item	Value
Acquired Date&Time	5.12.2022 09:43:26
Acquired by	System Administrator
Filename	C:\Users\dopnalab\Desktop\derya.ispd
Spectrum name	FPO-11
Sample name	FPO-1
Sample ID	
Option	
Comment	
No. of Scans	10
Resolution	4 [cm-1]
Apodization	Happ-Genzel

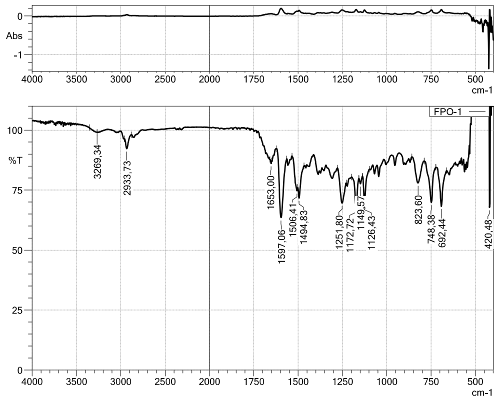

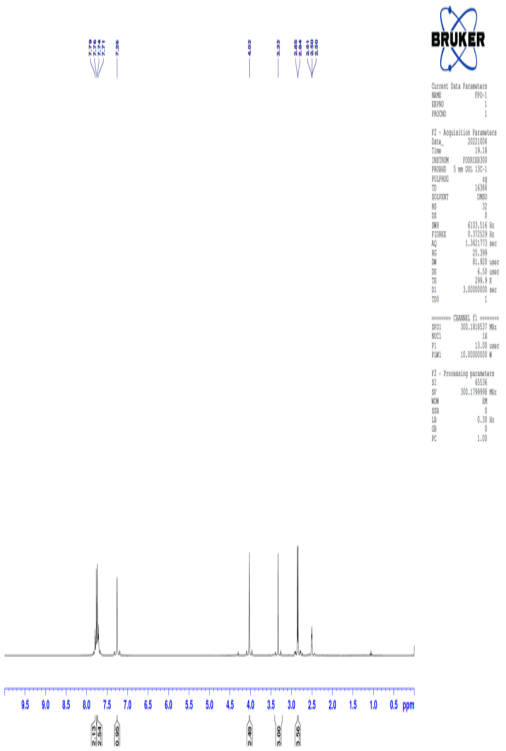

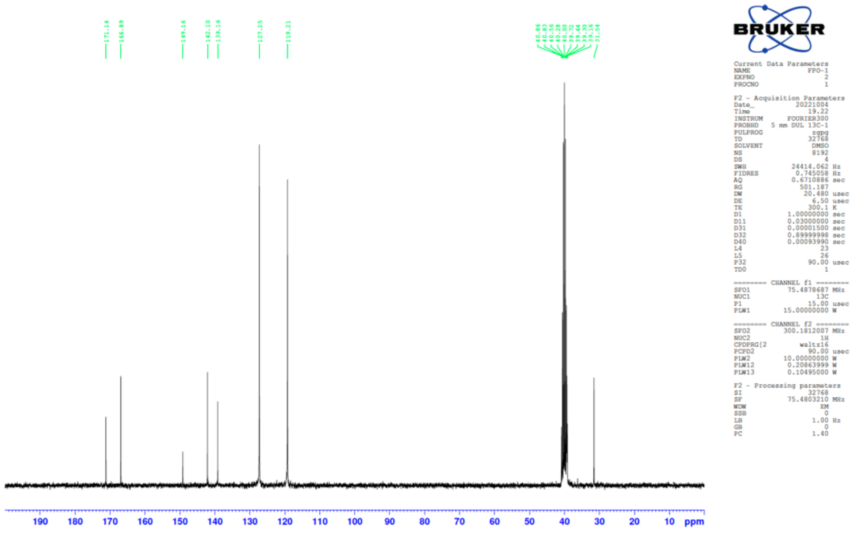

Figure S2. Chemical structure and spectral data of compound **4b**

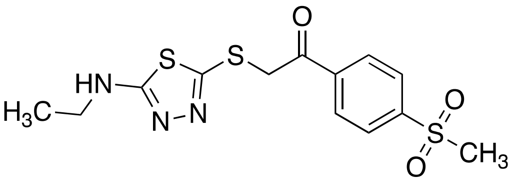

DOPNALAB

	Item	Value
	Acquired Date&Time	23.12.2022 14:58:50
	Acquired by	System Administrator
	Filename	C:\Users\dopnalab\Desktop\derya.ispd
	Spectrum name	FPO-12
	Sample name	FPO-2
	Sample ID	
	Option	
	Comment	
	No. of Scans	10
	Resolution	4 [cm-1]
	Apodization	Happ-Genzel

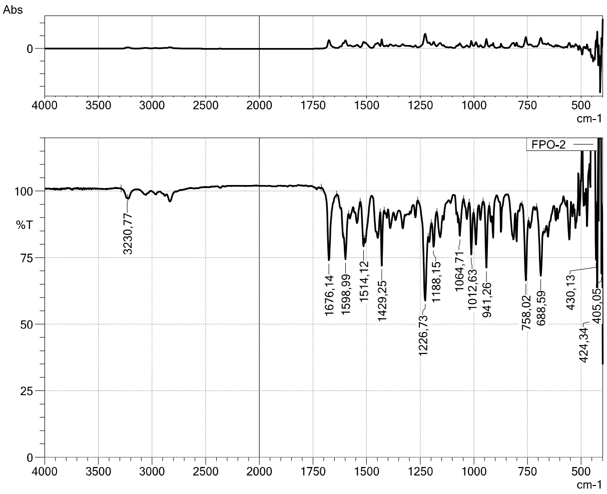

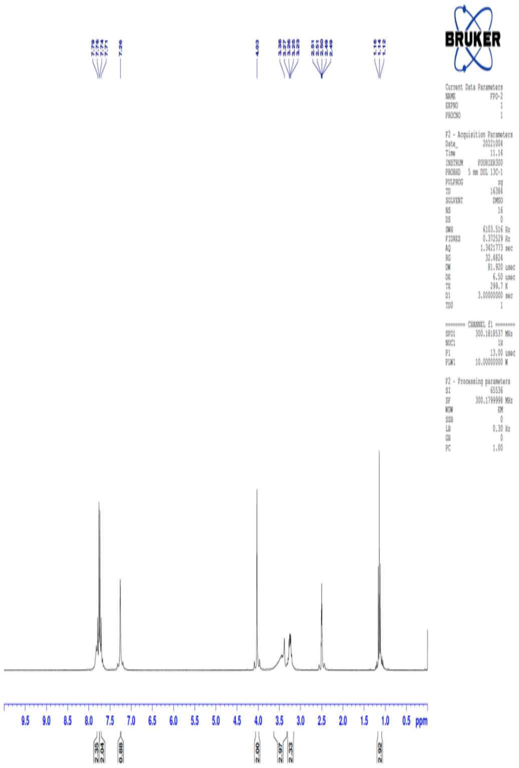

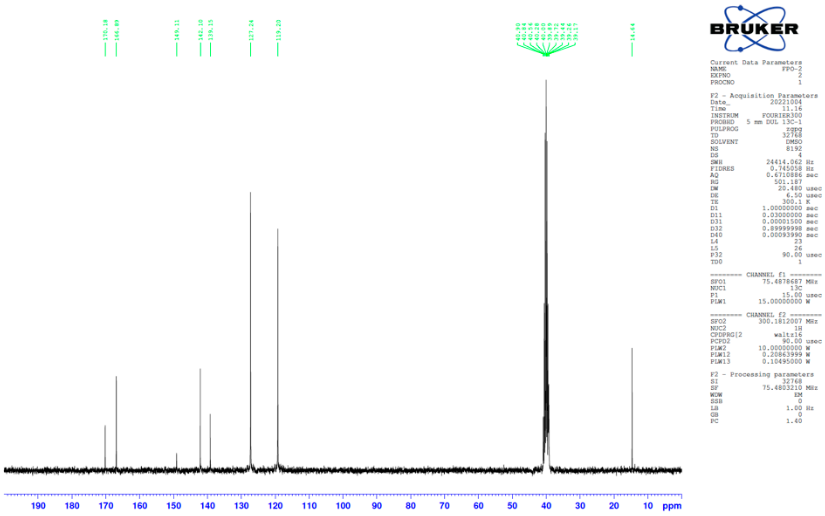

Figure S3. Chemical structure and spectral data of compound **4c**

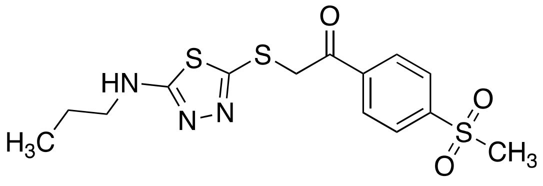

DOPNALAB

Item	Value
Acquired Date&Time	23.12.2022 15:03:05
Acquired by	System Administrator
Filename	C:\Users\dopnalab\Desktop\derya.ispd
Spectrum name	FPO-13
Sample name	FPO-3
Sample ID	
Option	
Comment	
No. of Scans	10
Resolution	4 [cm-1]
Apodization	Happ-Genzel

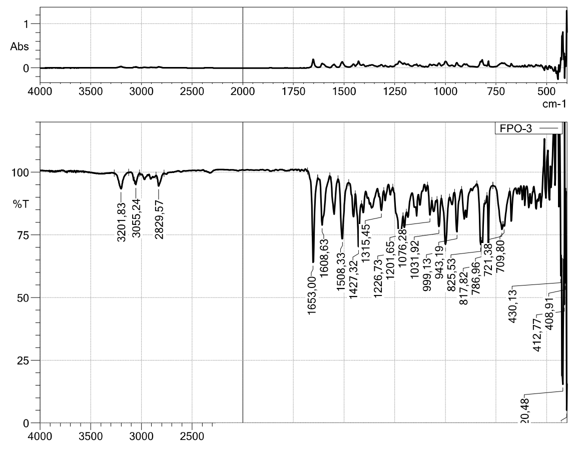

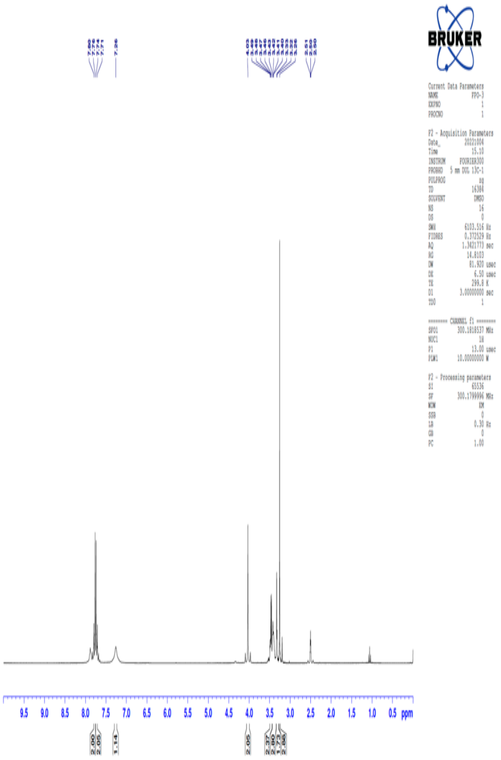

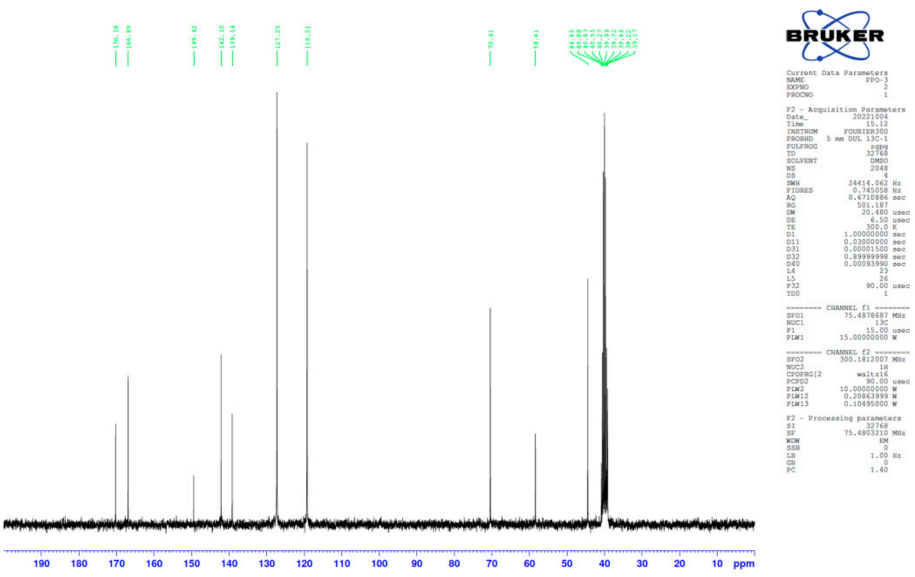

Figure S4. Chemical structure and spectral data of compound **4d**

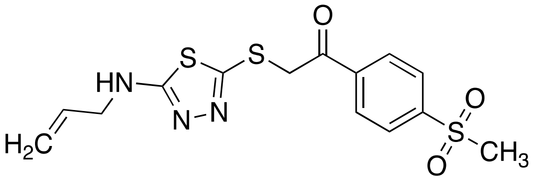

DOPNALAB

Item	Value
Acquired Date&Time	23.12.2022 15:06:08
Acquired by	System Administrator
Filename	C:\Users\dopnalab\Desktop\derya.ispd
Spectrum name	FPO-14
Sample name	FPO-4
Sample ID	
Option	
Comment	
No. of Scans	10
Resolution	4 [cm-1]
Apodization	Happ-Genzel

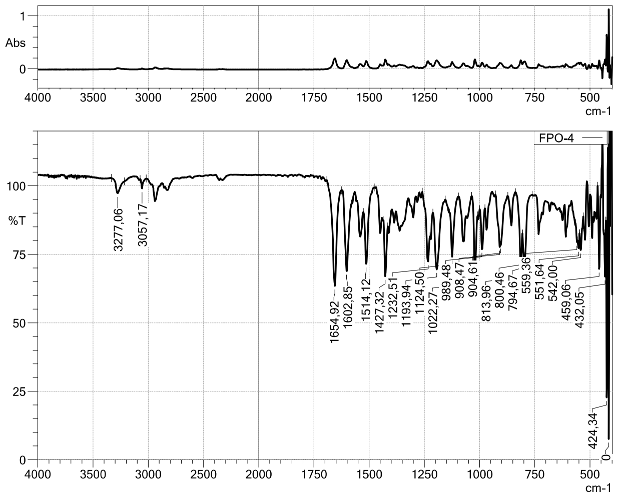

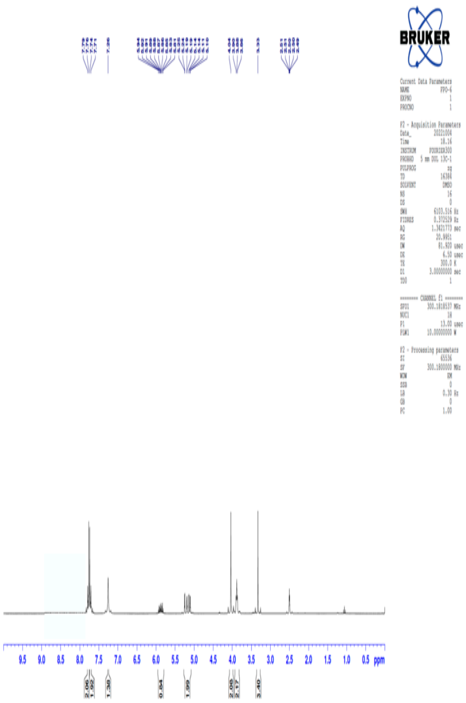

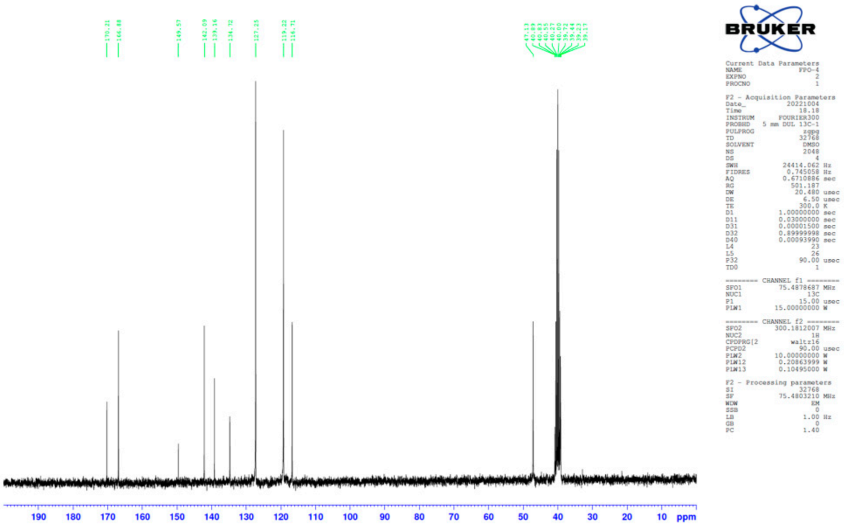

Figure S5. Chemical structure and spectral data of compound **4e**

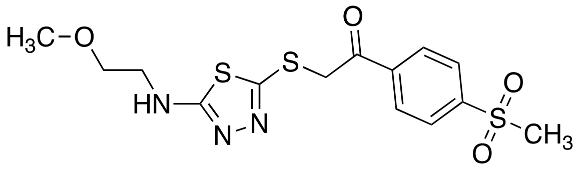

DOPNALAB

	Item	Value
	Acquired Date&Time	23.12.2022 15:09:51
	Acquired by	System Administrator
	Filename	C:\Users\dopnalab\Desktop\derya.ispd
	Spectrum name	FPO-15
	Sample name	FPO-5
	Sample ID	
	Option	
	Comment	
	No. of Scans	10
	Resolution	4 [cm-1]
	Apodization	Happ-Genzel

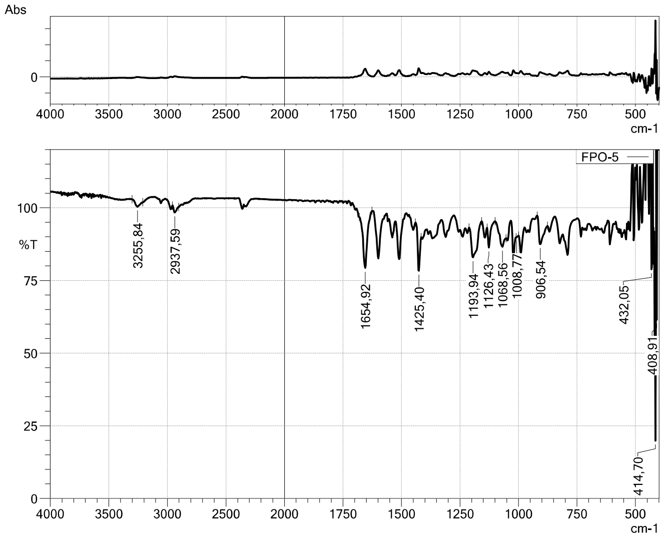

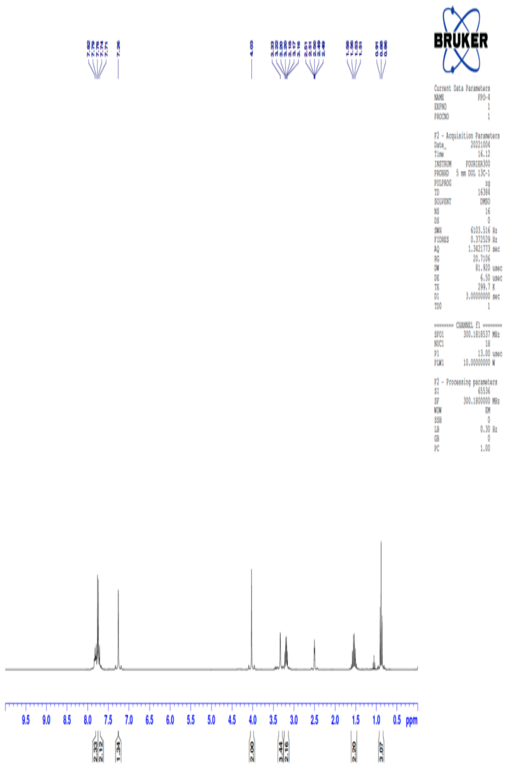

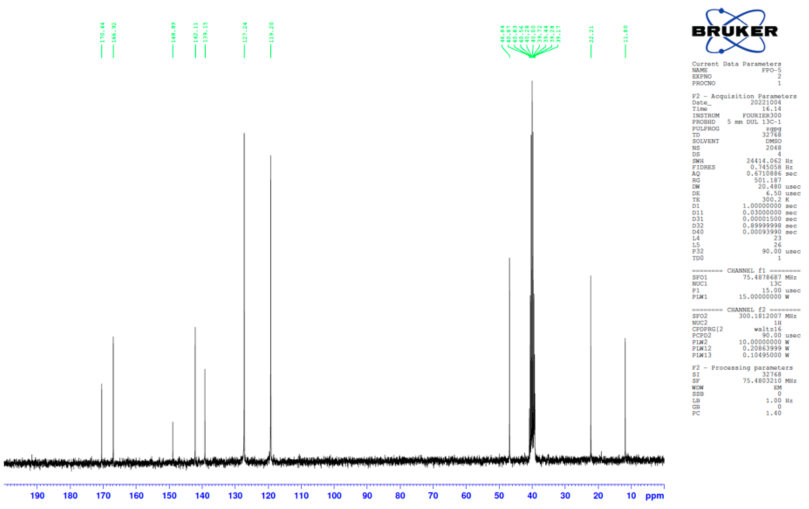

Figure S6. Chemical structure and spectral data of compound **4f**

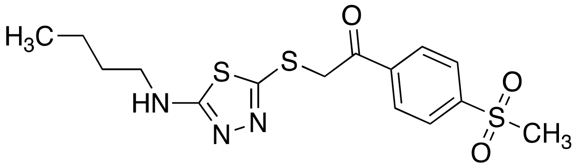

DOPNALAB

	Item	Value
	Acquired Date&Time	23.12.2022 15:12:02
	Acquired by	System Administrator
	Filename	C:\Users\dopnalab\Desktop\derya.ispd
	Spectrum name	FPO-16
	Sample name	FPO-6
	Sample ID	
	Option	
	Comment	
	No. of Scans	10
	Resolution	4 [cm-1]
	Apodization	Happ-Genzel

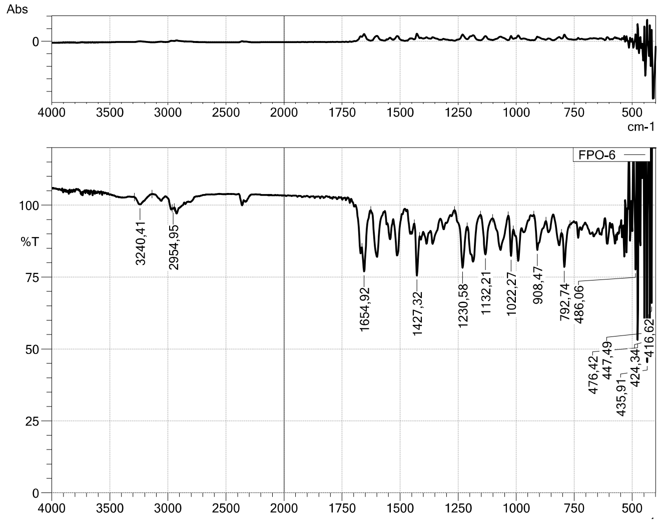

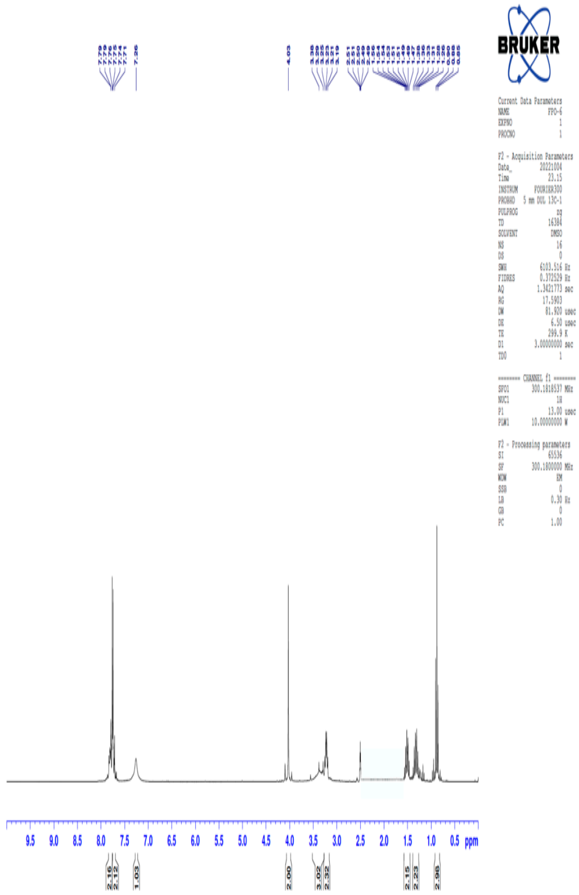

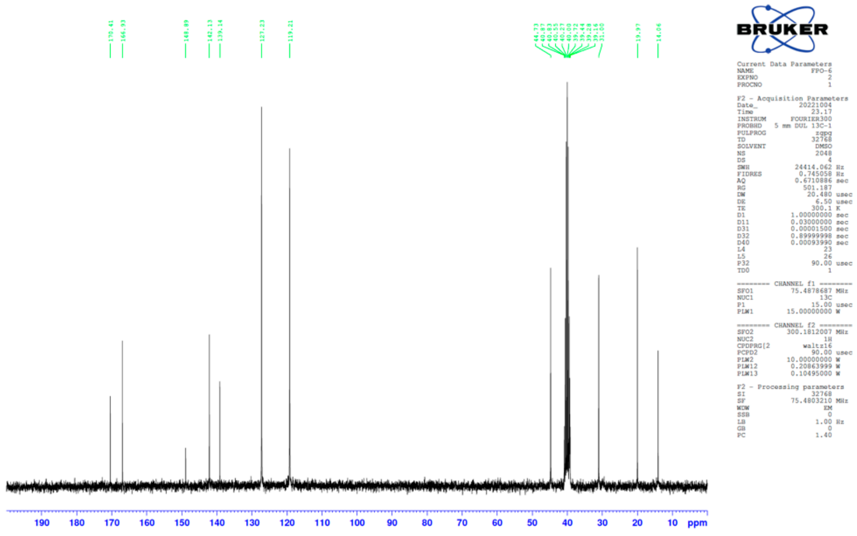

Figure S7. Chemical structure and spectral data of compound **4g**

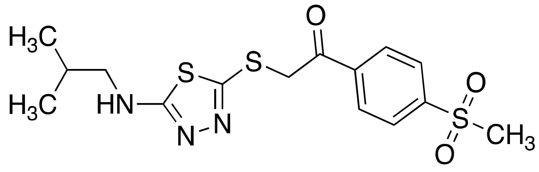

DOPNALAB

	Item	Value
	Acquired Date&Time	23.12.2022 15:53:23
	Acquired by	System Administrator
	Filename	C:\Users\dopnalab\Desktop\derya.ispd
	Spectrum name	FPO-17
	Sample name	FPO-7
	Sample ID	
	Option	
	Comment	
	No. of Scans	10
	Resolution	4 [cm-1]
	Apodization	Happ-Genzel

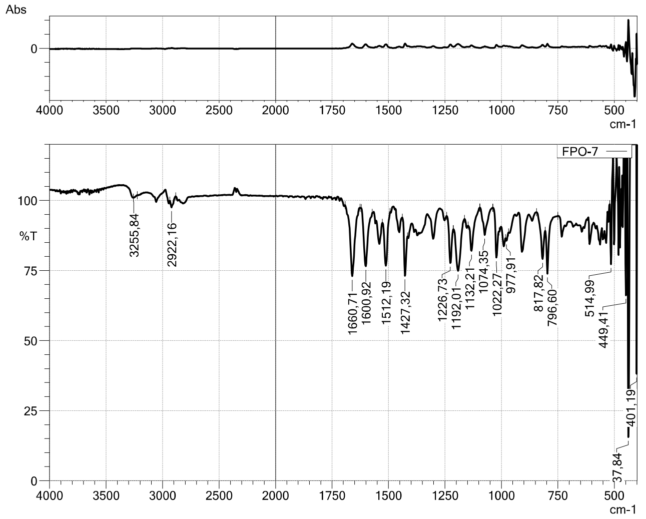

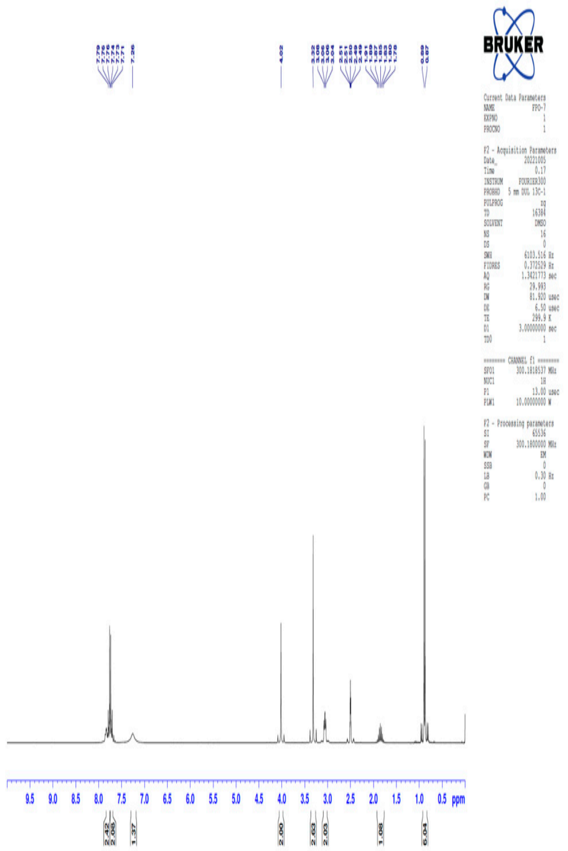

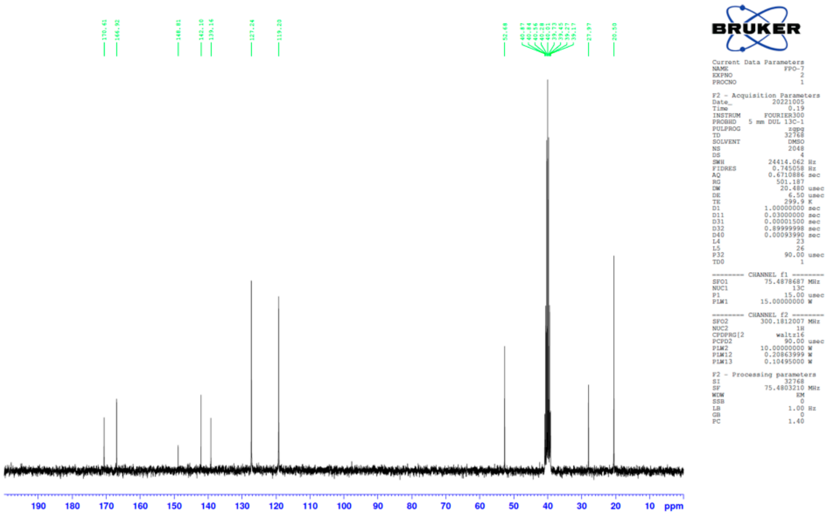

Figure S8. Chemical structure and spectral data of compound **4h**

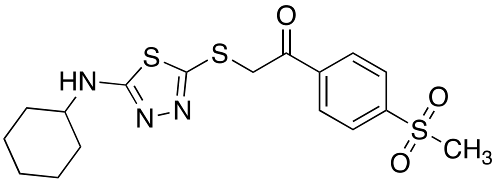

DOPNALAB

Item	Value
Acquired Date&Time	23.12.2022 15:16:48
Acquired by	System Administrator
Filename	C:\Users\dopnalab\Desktop\derya.ispd
Spectrum name	FPO-18
Sample name	FPO-8
Sample ID	
Option	
Comment	
No. of Scans	10
Resolution	4 [cm-1]
Apodization	Happ-Genzel

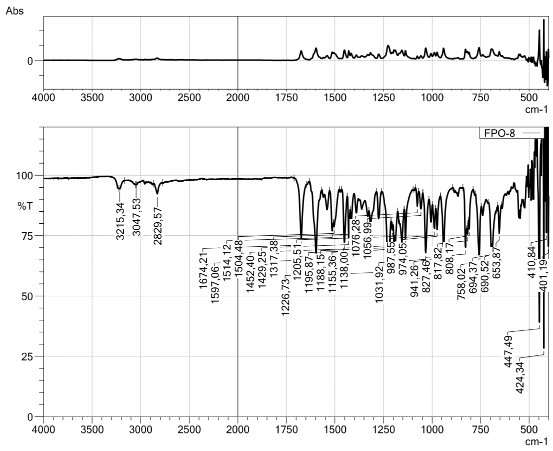

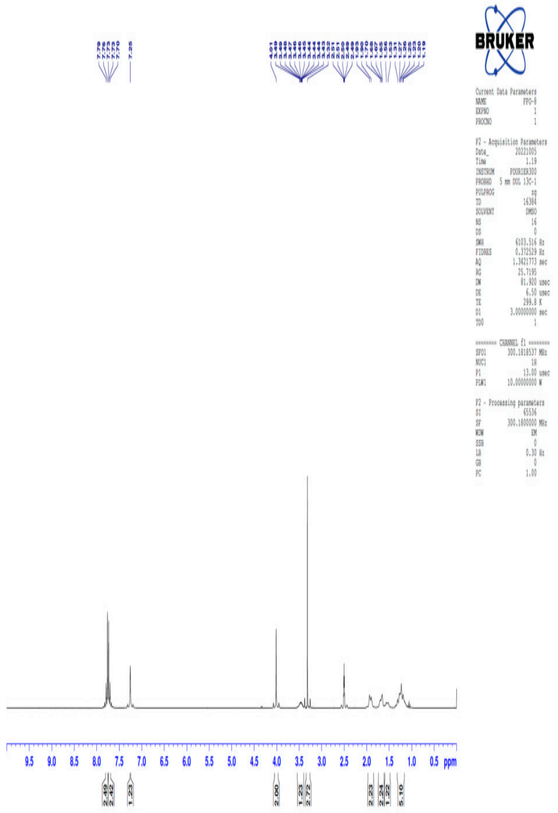

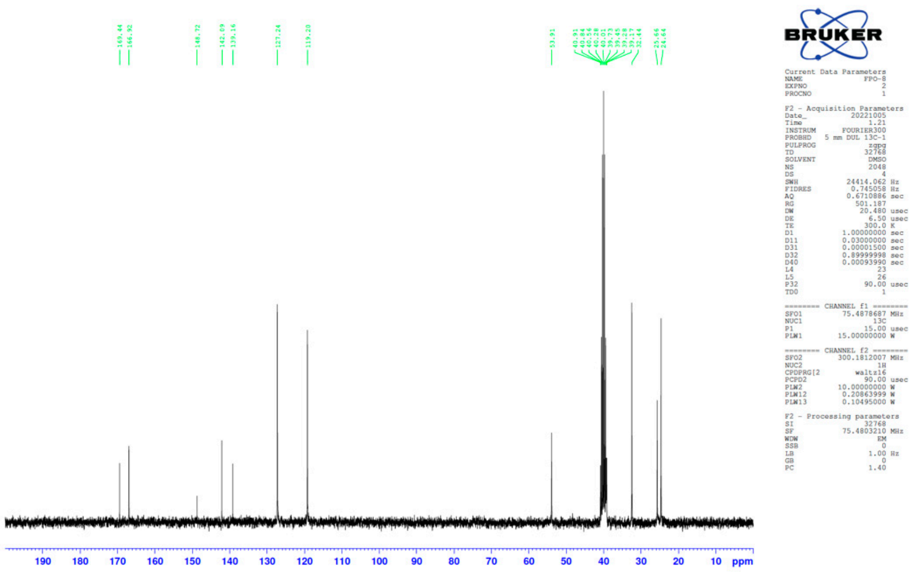

Figure S9. Chemical structure and spectral data of compound **4i**

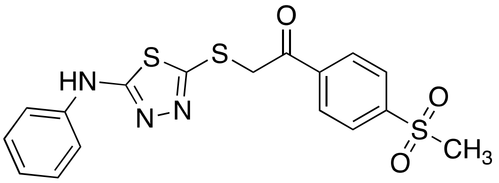

DOPNALAB

Item	Value
Acquired Date&Time	23.12.2022 15:55:32
Acquired by	System Administrator
Filename	C:\Users\dopnalab\Desktop\derya.ispd
Spectrum name	FPO-19
Sample name	FPO-9
Sample ID	
Option	
Comment	
No. of Scans	10
Resolution	4 [cm-1]
Apodization	Happ-Genzel

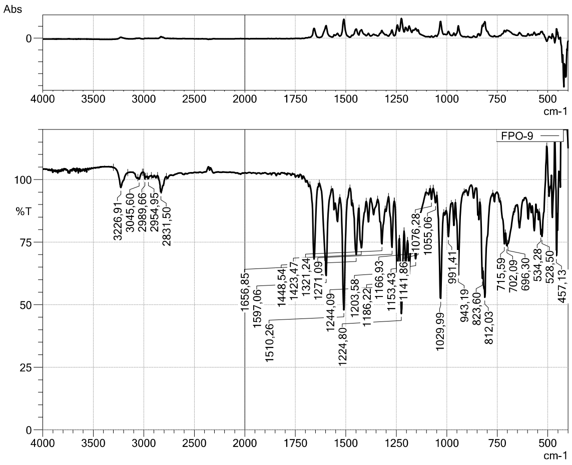

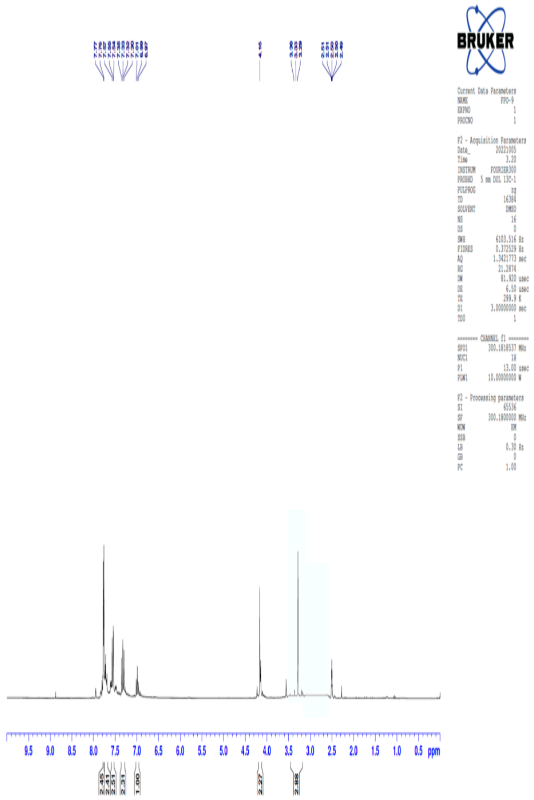

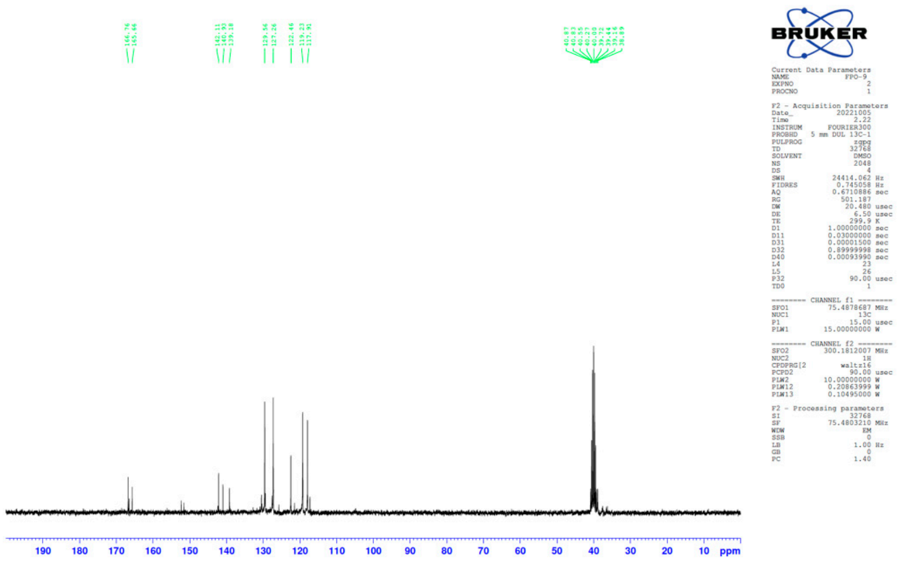

Figure S10. Chemical structure and spectral data of compound **4j**

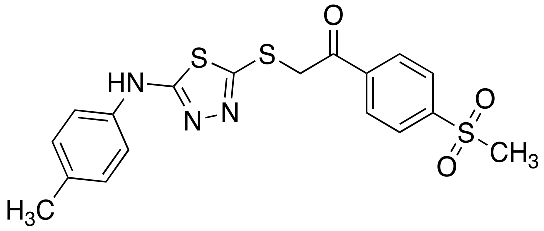

DOPNALAB

Item	Value
Acquired Date&Time	23.12.2022 15:57:46
Acquired by	System Administrator
Filename	C:\Users\dopnalab\Desktop\derya.ispd
Spectrum name	FPO-110
Sample name	FPO-10
Sample ID	
Option	
Comment	
No. of Scans	10
Resolution	4 [cm-1]
Apodization	Happ-Genzel

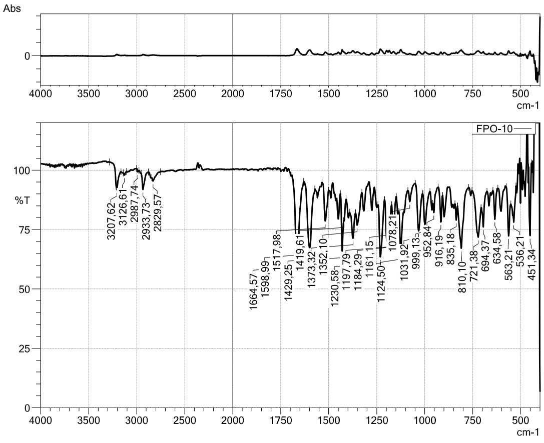

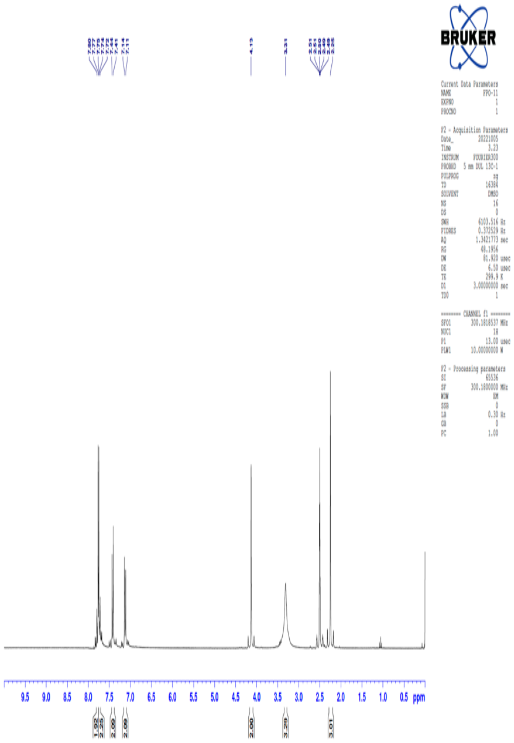

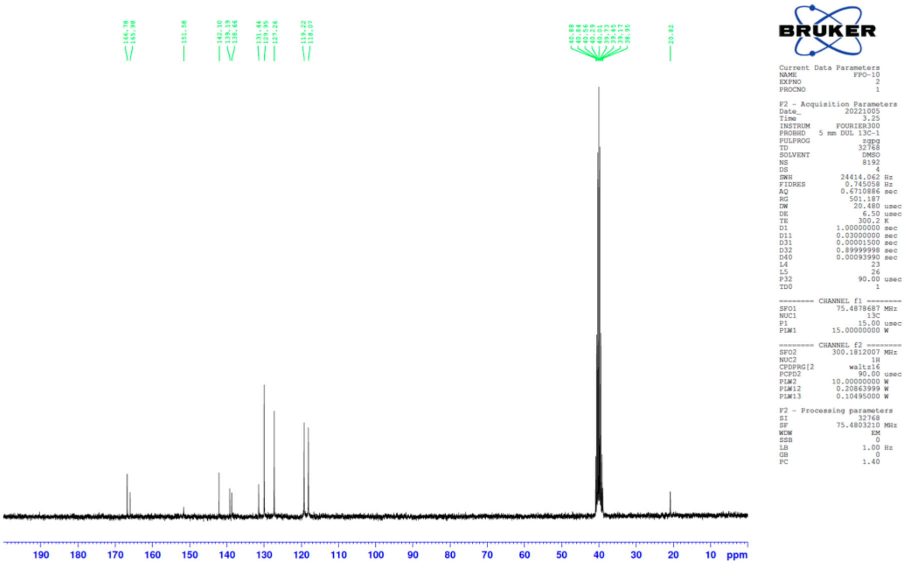

Figure S11. Chemical structure and spectral data of compound **4k**

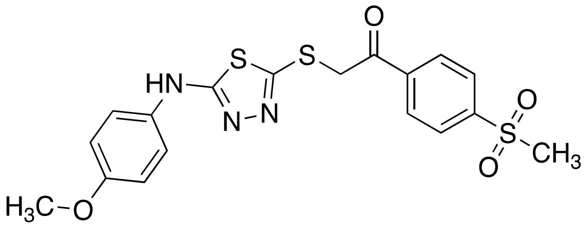

DOPNALAB

Item	Value
Acquired Date&Time	23.12.2022 16:04:09
Acquired by	System Administrator
Filename	C:\Users\dopnalab\Desktop\derya.ispd
Spectrum name	FPO-111
Sample name	FPO-11
Sample ID	
Option	
Comment	
No. of Scans	10
Resolution	4 [cm-1]
Apodization	Happ-Genzel

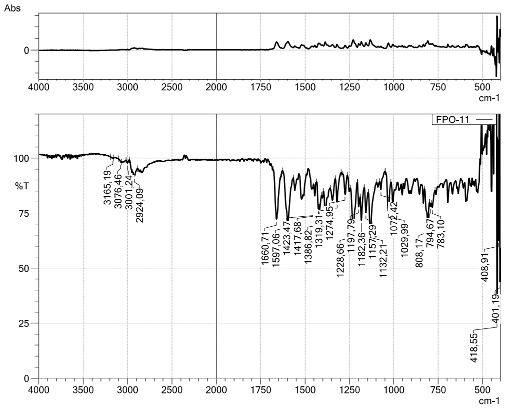

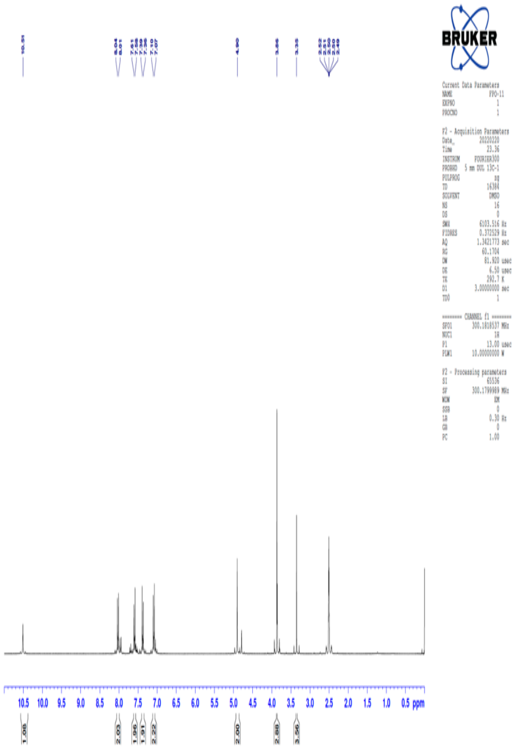

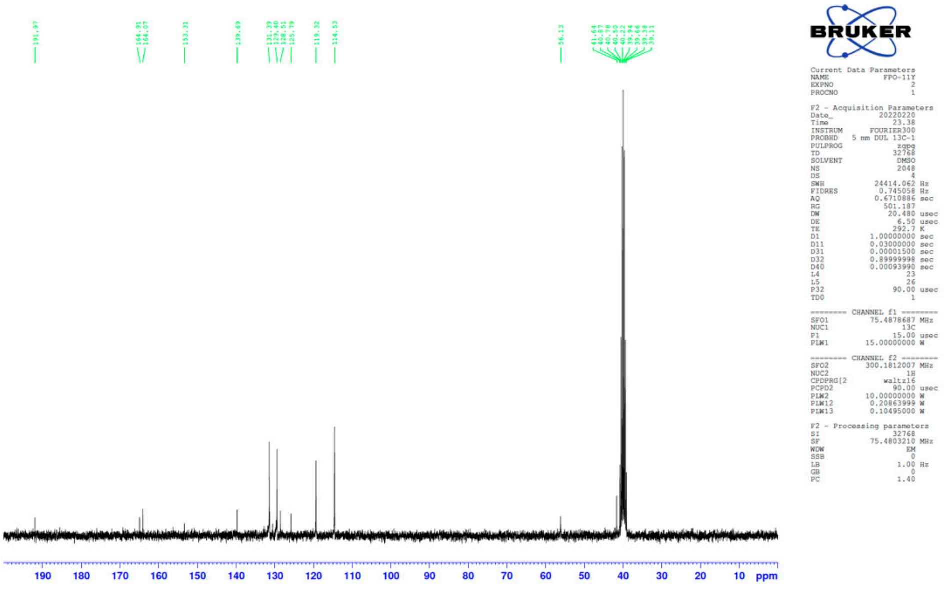

Figure S12. Chemical structure and spectral data of compound **4l**

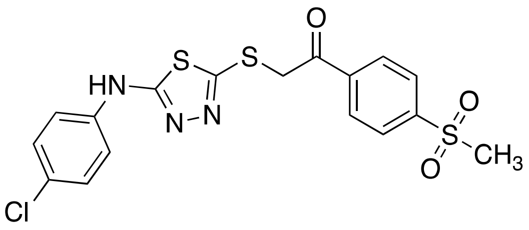

DOPNALAB

Item	Value
Acquired Date&Time	23.12.2022 16:07:42
Acquired by	System Administrator
Filename	C:\Users\dopnallab\Desktop\derya.ispd
Spectrum name	FPO-112
Sample name	FPO-12
Sample ID	
Option	
Comment	
No. of Scans	10
Resolution	4 [cm-1]
Apodization	Happ-Genzel

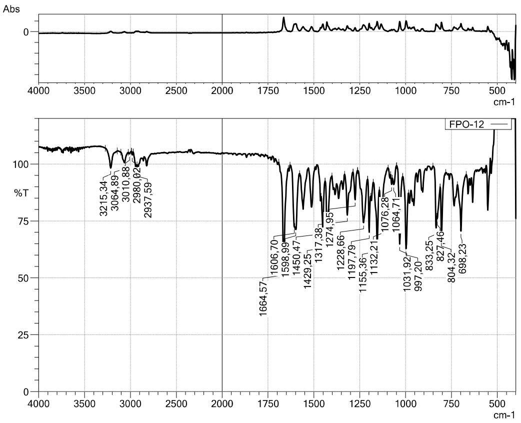

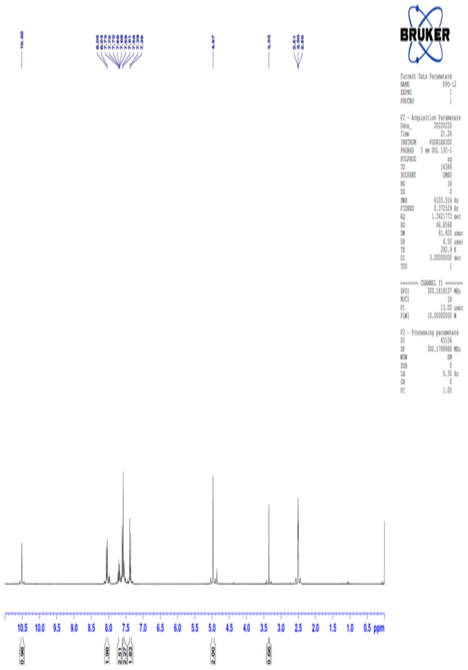

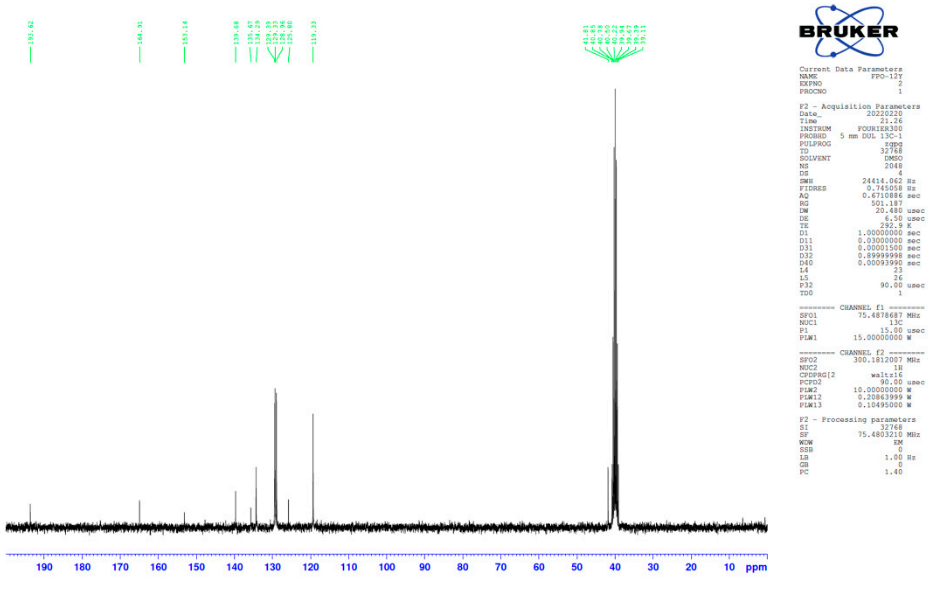

Figure S13. HRMS spectrum of compound **4a**

Formula Predictor Report - FPO-1__53.lcd	Page 1 of 1

Data File: C:\LabSolutions\Data\Analiz\Derya\2\FPO-1__53.lcd

Elmt	Val.	Min	Max	Elmt	Val.	Min	Max	Elmt	Val.	Min	Max	Elmt	Val.	Min	Max	Use Adduct
H	1	10	33	O	2	0	5	S	2	0	3	Ru	2	0	0	H
C	4	10	30	F	1	0	0	Cl	1	0	0	Pd	2	0	0	
N	3	3	6	P	3	0	0	Br	1	0	0	I	3	0	0	

Error Margin (ppm):	5	DBE Range:	8.0 - 30.0	Electron Ions:	both
HC Ratio:	unlimited	Apply N Rule:	yes	Use MSn Info:	yes
Max Isotopes:	5	Isotope RI (%):	1.00	Isotope Res:	9000
MSn Iso RI (%):	10.00	MSn Logic Mode:	AND	Max Results:	50

Event#: 1 MS(E+) Ret. Time : 2.240 -> 2.373 Scan# : 337 -> 357

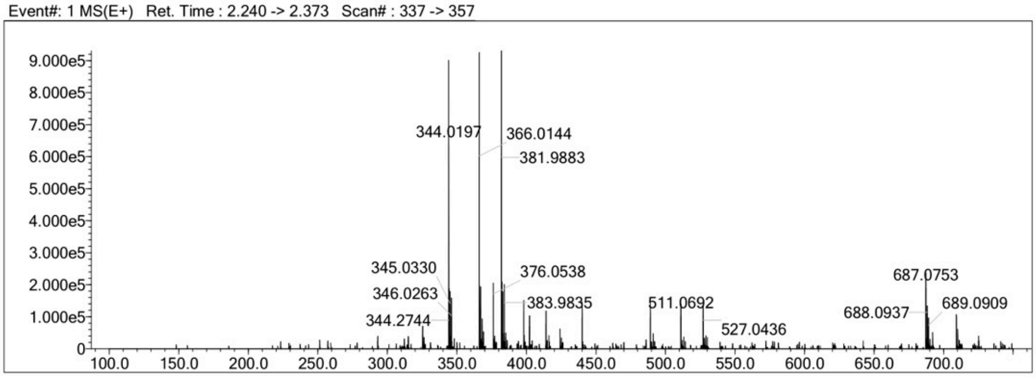

Measured region for 344.0197 m/z

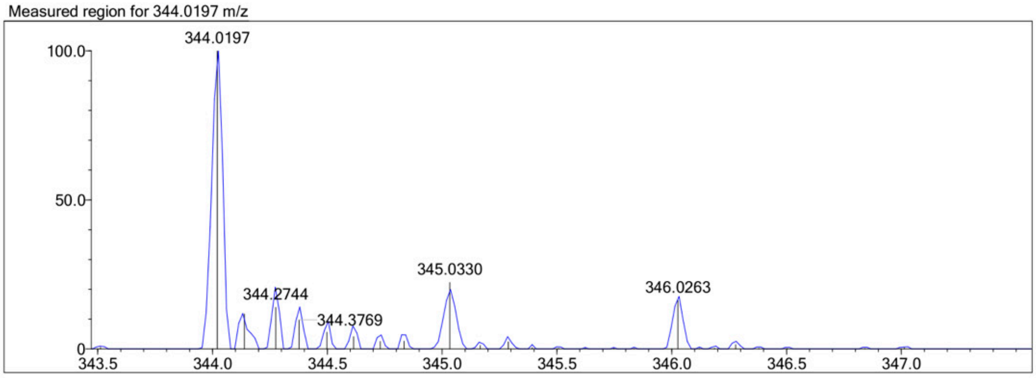

C12 H13 N3 O3 S3 [M+H]+ : Predicted region for 344.0192 m/z

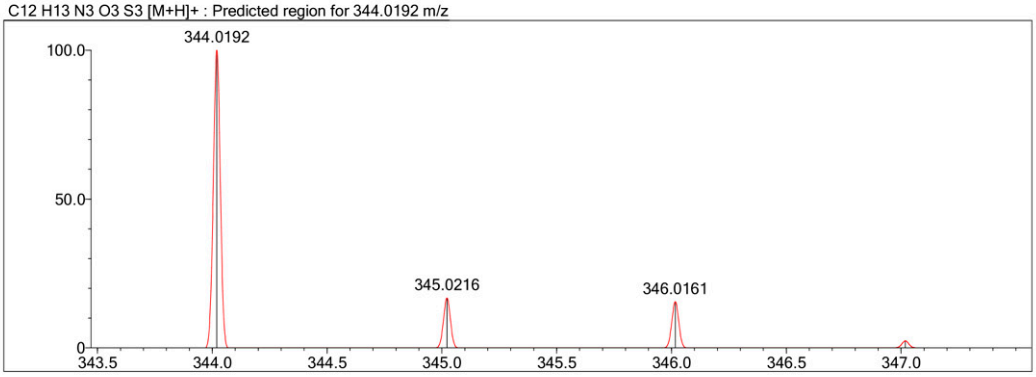

Rank	Score	Formula (M)	Ion	Meas. m/z	Pred. m/z	Df. (mDa)	Df. (ppm)	Iso	DBE
1	65.64	C12 H13 N3 O3 S3	[M+H]+	344.0197	344.0192	0.5	1.45	66.38	8.0

Figure S14. HRMS spectrum of compound **4b**

Formula Predictor Report - FPO-2__54.lcd | Page 1 of 1

Data File: C:\LabSolutions\Data\Analiz\Derya\2\FPO-2__54.lcd

Elmt	Val.	Min	Max	Elmt	Val.	Min	Max	Elmt	Val.	Min	Max	Elmt	Val.	Min	Max	Use Adduct
H	1	10	33	O	2	0	5	S	2	0	3	Ru	2	0	0	H
C	4	10	30	F	1	0	0	Cl	1	0	0	Pd	2	0	0	
N	3	3	6	P	3	0	0	Br	1	0	0	I	3	0	0	

Error Margin (ppm):	5	DBE Range:	8.0 - 30.0	Electron Ions:	both
HC Ratio:	unlimited	Apply N Rule:	yes	Use MSn Info:	yes
Max Isotopes:	5	Isotope RI (%):	1.00	Isotope Res:	9000
MSn Iso RI (%):	10.00	MSn Logic Mode:	AND	Max Results:	50

Event#: 1 MS(E+) Ret. Time : 2.280 -> 2.493 Scan# : 343 -> 375

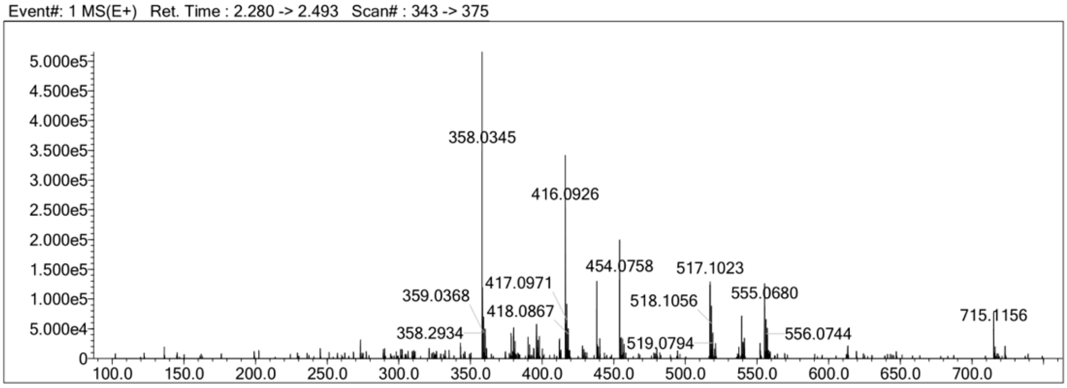

Measured region for 358.0345 m/z

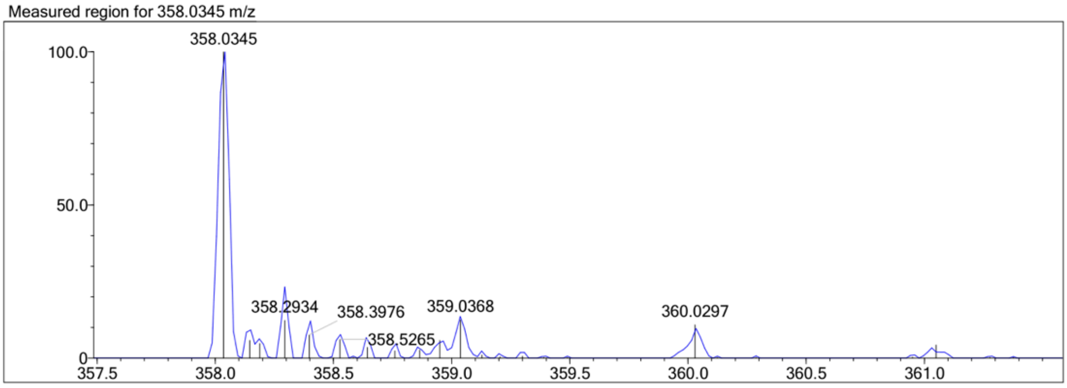

C13 H15 N3 O3 S3 [M+H]+ : Predicted region for 358.0348 m/z

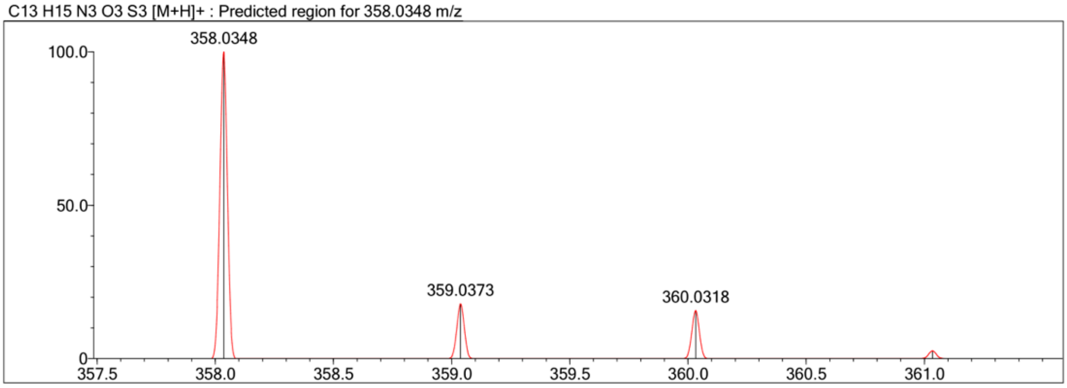

Rank	Score	Formula (M)	Ion	Meas. m/z	Pred. m/z	Df. (mDa)	Df. (ppm)	Iso	DBE
1	73.49	C13 H15 N3 O3 S3	[M+H]+	358.0345	358.0348	-0.3	-0.84	73.49	8.0

Figure S15. HRMS spectrum of compound **4c**

Formula Predictor Report - FPO-3__55.lcd

Page 1 of 1

Data File: C:\LabSolutions\Data\Analiz\Derya\2\FPO-3__55.lcd

Elmt	Val.	Min	Max	Elmt	Val.	Min	Max	Elmt	Val.	Min	Max	Elmt	Val.	Min	Max	Use Adduct
H	1	10	33	O	2	0	5	S	2	0	3	Ru	2	0	0	H
C	4	10	30	F	1	0	0	Cl	1	0	0	Pd	2	0	0	
N	3	3	6	P	3	0	0	Br	1	0	0	I	3	0	0	

Error Margin (ppm):	5	DBE Range:	8.0 - 30.0	Electron Ions:	both
HC Ratio:	unlimited	Apply N Rule:	yes	Use MSn Info:	yes
Max Isotopes:	5	Isotope RI (%):	1.00	Isotope Res:	9000
MSn Iso RI (%):	10.00	MSn Logic Mode:	AND	Max Results:	50

Event#: 1 MS(E+) Ret. Time : 2.347 -> 2.560 Scan# : 353 -> 385

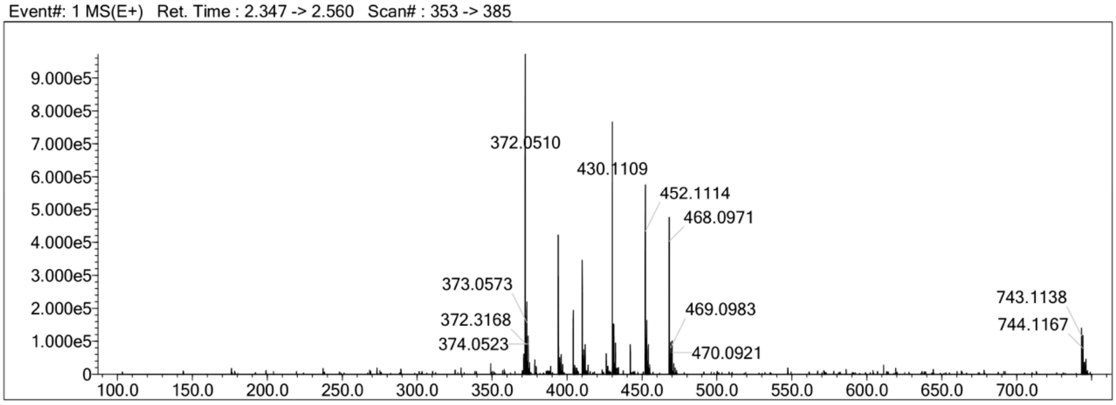

Measured region for 372.0510 m/z

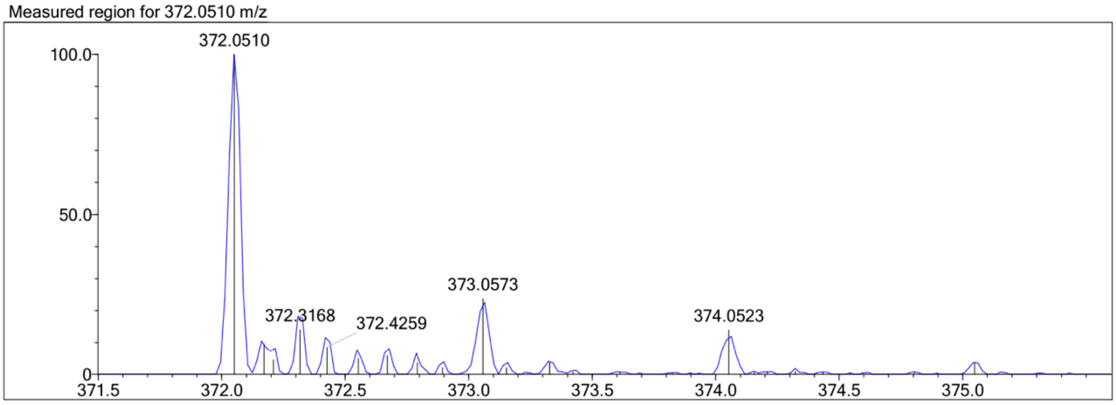

C14 H17 N3 O3 S3 [M+H]+ : Predicted region for 372.0505 m/z

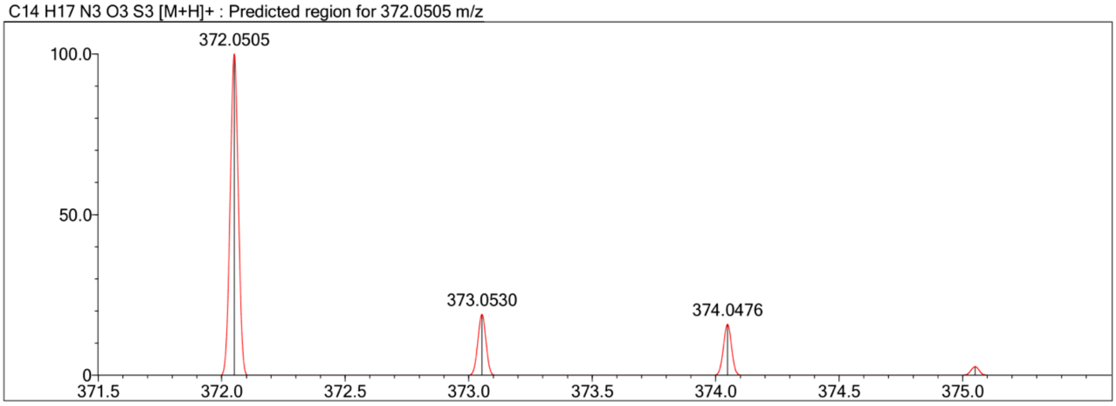

Rank	Score	Formula (M)	Ion	Meas. m/z	Pred. m/z	Df. (mDa)	Df. (ppm)	Iso	DBE
1	89.41	C14 H17 N3 O3 S3	[M+H]+	372.0510	372.0505	0.5	1.34	90.18	8.0

Figure S16. HRMS spectrum of compound **4d**

Formula Predictor Report - FPO-4__56.lcd

Data File: C:\LabSolutions\Data\Analiz\Derya\2\FPO-4__56.lcd

Elmt	Val.	Min	Max	Elmt	Val.	Min	Max	Elmt	Val.	Min	Max	Elmt	Val.	Min	Max	Use Adduct
H	1	10	33	O	2	0	5	S	2	0	3	Ru	2	0	0	H
C	4	10	30	F	1	0	0	Cl	1	0	0	Pd	2	0	0	
N	3	3	6	P	3	0	0	Br	1	0	0	I	3	0	0	

Error Margin (ppm):	5	DBE Range:	8.0 - 30.0	Electron Ions:	both
HC Ratio:	unlimited	Apply N Rule:	yes	Use MSn Info:	yes
Max Isotopes:	5	Isotope RI (%):	1.00	Isotope Res:	9000
MSn Iso RI (%):	10.00	MSn Logic Mode:	AND	Max Results:	50

Event#: 1 MS(E+) Ret. Time : 2.240 -> 2.427 Scan# : 337 -> 365

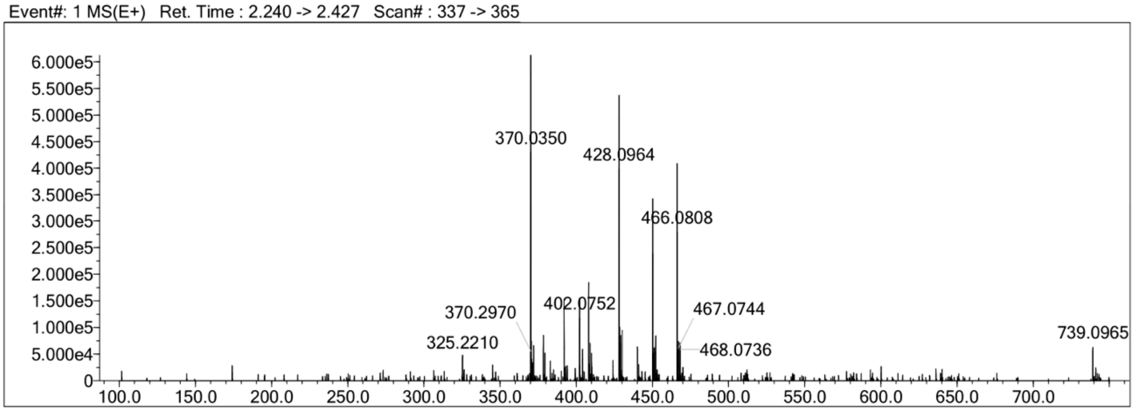

Measured region for 370.0350 m/z

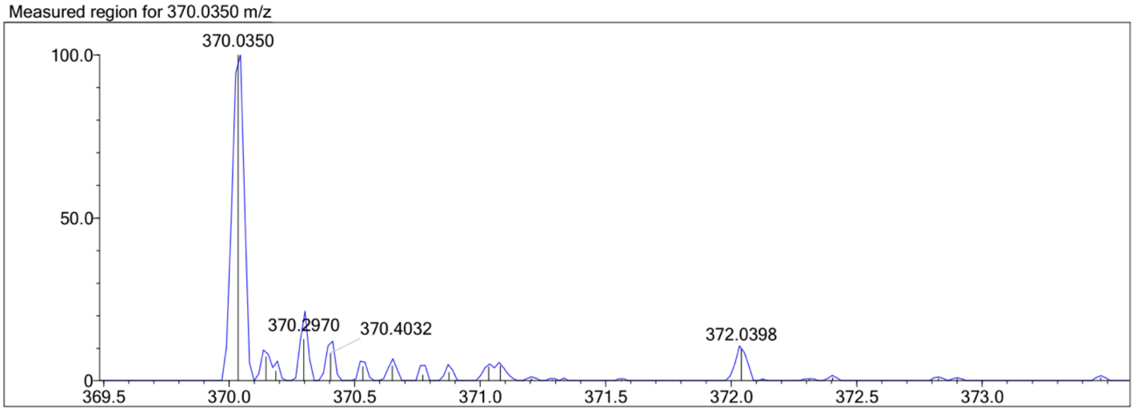

C14 H15 N3 O3 S3 [M+H]+ : Predicted region for 370.0348 m/z

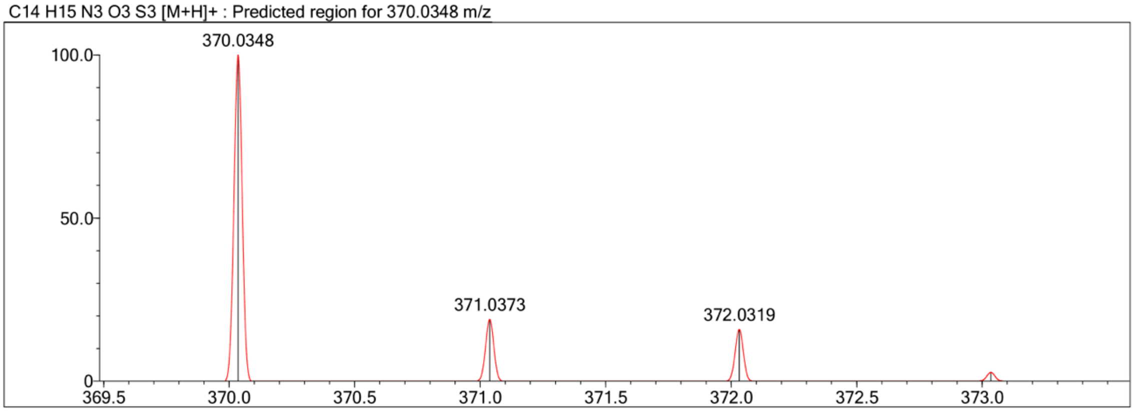

Rank	Score	Formula (M)	Ion	Meas. m/z	Pred. m/z	Df. (mDa)	Df. (ppm)	Iso	DBE
1	56.37	C14 H15 N3 O3 S3	[M+H]+	370.0350	370.0348	0.2	0.54	56.37	9.0

Figure S17. HRMS spectrum of compound **4e**

Formula Predictor Report - 1_21.04.2026_FPO-5_2.lcd

Page 1 of 1

Data File: C:\LabSolutions\Data\Analiz\AEE\1_21.04.2026_FPO-5_2.lcd

Elmt	Val.	Min	Max	Elmt	Val.	Min	Max	Elmt	Val.	Min	Max	Elmt	Val.	Min	Max	Use Adduct
H	1	10	33	O	2	1	5	S	2	0	3	Ru	2	0	0	H
C	4	10	21	F	1	0	0	Cl	1	0	0	Pd	2	0	0	
N	3	3	6	P	3	0	0	Br	1	0	0	I	3	0	0	

Error Margin (ppm): 5
HC Ratio: unlimited
Max Isotopes: 5
MSn Iso RI (%): 10.00

DBE Range: 8.0 - 30.0
Apply N Rule: yes
Isotope RI (%): 1.00
MSn Logic Mode: AND

Electron Ions: both
Use MSn Info: yes
Isotope Res: 9000
Max Results: 50

Event#: 1 MS(E+) Ret. Time : 2.187 Scan# : 329

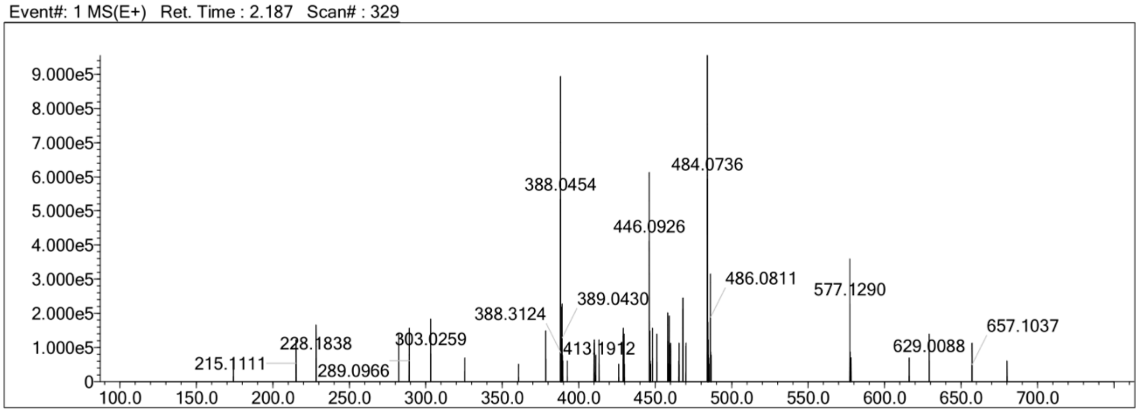

Measured region for 388.0454 m/z

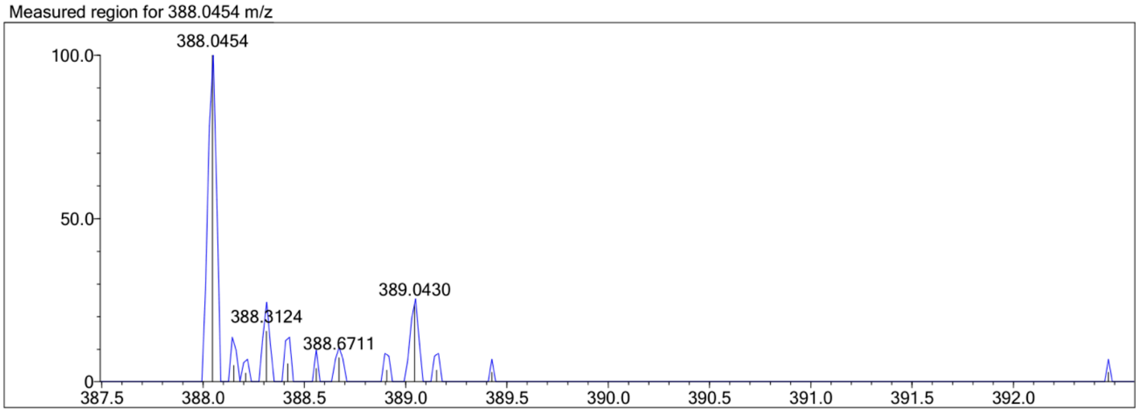

C14 H17 N3 O4 S3 [M+H]+ : Predicted region for 388.0454 m/z

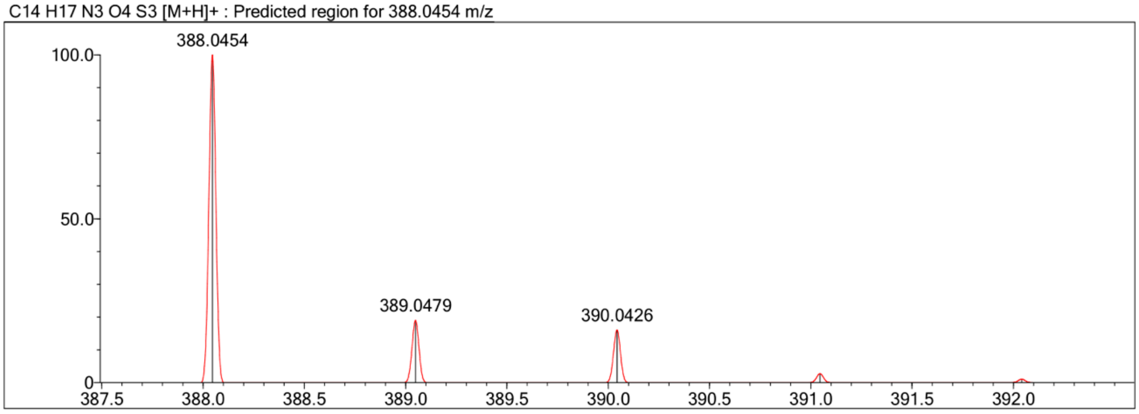

Rank	Score	Formula (M)	Ion	Meas. m/z	Pred. m/z	Df. (mDa)	Df. (ppm)	Iso	DBE
1	0.00	C14 H17 N3 O4 S3	[M+H]+	388.0454	388.0454	0.0	0.00	0.00	8.0

Figure S18. HRMS spectrum of compound **4f**

Data File: C:\LabSolutions\Data\Analiz\Derya\2\FPO-6__57.lcd

Elmt	Val.	Min	Max	Elmt	Val.	Min	Max	Elmt	Val.	Min	Max	Elmt	Val.	Min	Max	Use Adduct
H	1	10	33	O	2	0	5	S	2	0	3	Ru	2	0	0	H
C	4	10	30	F	1	0	0	Cl	1	0	0	Pd	2	0	0	
N	3	3	6	P	3	0	0	Br	1	0	0	I	3	0	0	

Error Margin (ppm):	5	DBE Range:	8.0 - 30.0	Electron Ions:	both
HC Ratio:	unlimited	Apply N Rule:	yes	Use MSn Info:	yes
Max Isotopes:	5	Isotope RI (%):	1.00	Isotope Res:	9000
MSn Iso RI (%):	10.00	MSn Logic Mode:	AND	Max Results:	50

Event#: 1 MS(E+) Ret. Time : 2.707 Scan# : 407

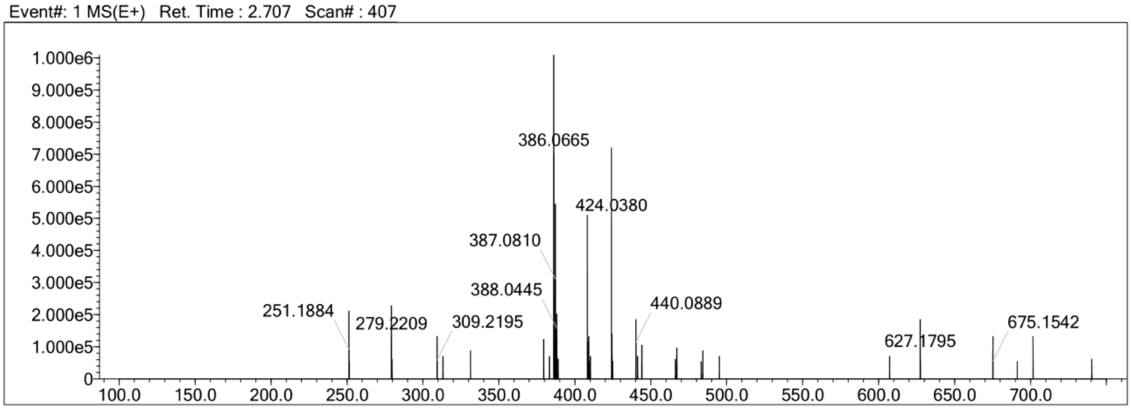

Measured region for 386.0665 m/z

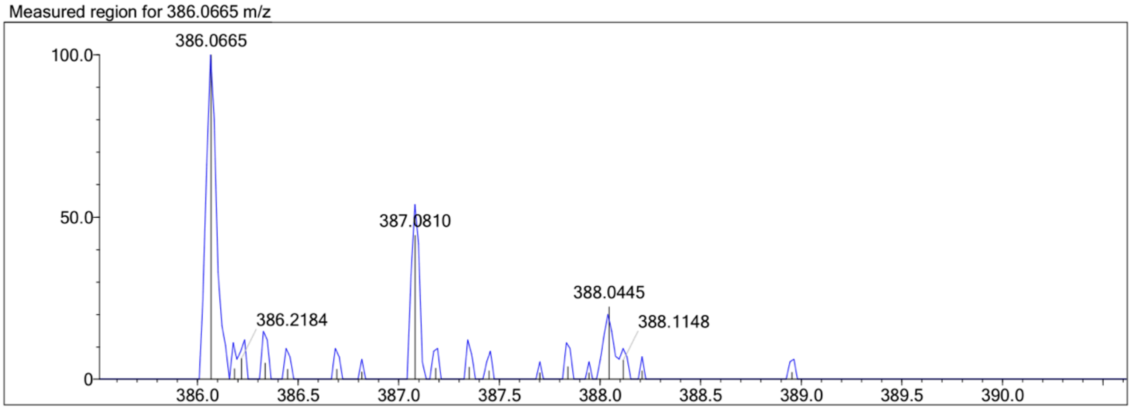

C15 H19 N3 O3 S3 [M+H]+ : Predicted region for 386.0661 m/z

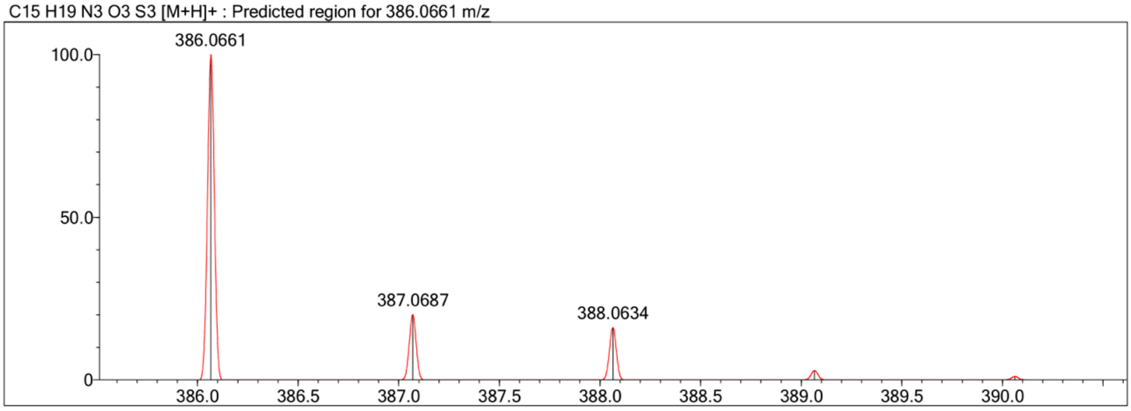

Rank	Score	Formula (M)	Ion	Meas. m/z	Pred. m/z	Df. (mDa)	Df. (ppm)	Iso	DBE
1	0.00	C15 H19 N3 O3 S3	[M+H]+	386.0665	386.0661	0.4	1.04	0.00	8.0

Figure S19. HRMS spectrum of compound **4g**

Formula Predictor Report - FPO-7__58.lcd

Data File: C:\LabSolutions\Data\Analiz\Derya\2\FPO-7__58.lcd

Elmt	Val.	Min	Max	Elmt	Val.	Min	Max	Elmt	Val.	Min	Max	Elmt	Val.	Min	Max	Use Adduct
H	1	10	33	O	2	0	5	S	2	0	3	Ru	2	0	0	H
C	4	10	30	F	1	0	0	Cl	1	0	0	Pd	2	0	0	
N	3	3	6	P	3	0	0	Br	1	0	0	I	3	0	0	

Error Margin (ppm):	5	DBE Range:	8.0 - 30.0	Electron Ions:	both
HC Ratio:	unlimited	Apply N Rule:	yes	Use MSn Info:	yes
Max Isotopes:	5	Isotope RI (%):	1.00	Isotope Res:	9000
MSn Iso RI (%):	10.00	MSn Logic Mode:	AND	Max Results:	50

Event#: 1 MS(E+) Ret. Time : 2.747 Scan# : 413

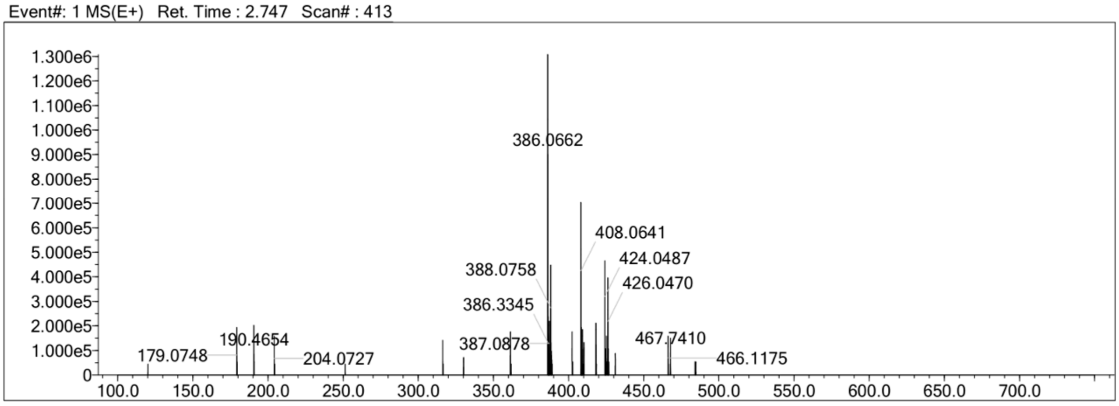

Measured region for 386.0662 m/z

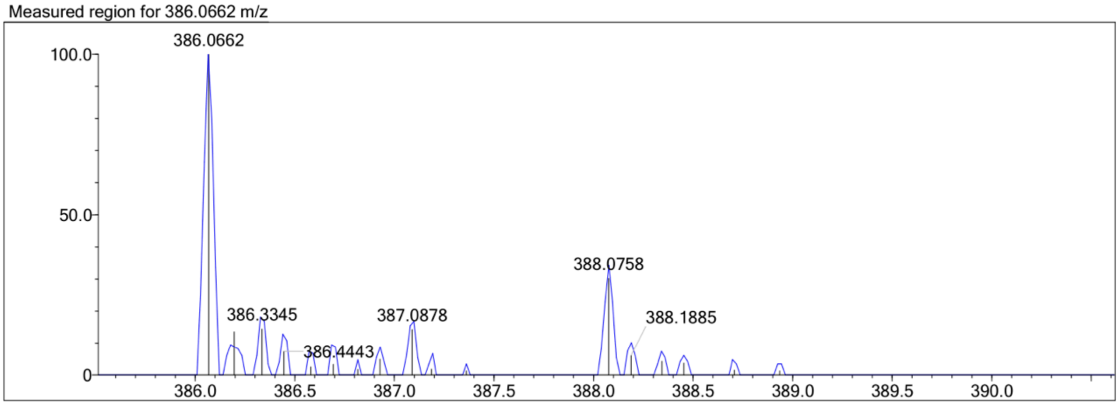

C15 H19 N3 O3 S3 [M+H]+ : Predicted region for 386.0661 m/z

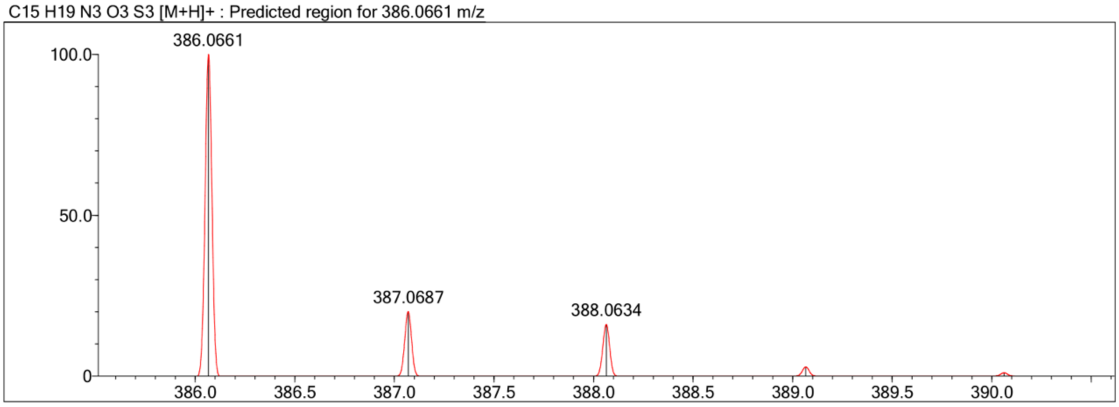

Rank	Score	Formula (M)	Ion	Meas. m/z	Pred. m/z	Df. (mDa)	Df. (ppm)	Iso	DBE
1	0.00	C15 H19 N3 O3 S3	[M+H]+	386.0662	386.0661	0.1	0.26	0.00	8.0

Figure S20. HRMS spectrum of compound **4h**

Formula Predictor Report - FPO-8__59.lcd

Data File: C:\LabSolutions\Data\Analiz\Derya\2\FPO-8__59.lcd

Elmt	Val.	Min	Max	Elmt	Val.	Min	Max	Elmt	Val.	Min	Max	Elmt	Val.	Min	Max	Use Adduct
H	1	10	33	O	2	0	5	S	2	0	3	Ru	2	0	0	H
C	4	10	30	F	1	0	0	Cl	1	0	0	Pd	2	0	0	
N	3	3	6	P	3	0	0	Br	1	0	0	I	3	0	0	

Error Margin (ppm): 5
HC Ratio: unlimited
Max Isotopes: 5
MSn Iso RI (%): 10.00

DBE Range: 8.0 - 30.0
Apply N Rule: yes
Isotope RI (%): 1.00
MSn Logic Mode: AND

Electron Ions: both
Use MSn Info: yes
Isotope Res: 9000
Max Results: 50

Event#: 1 MS(E+) Ret. Time : 3.000 -> 3.267 Scan# : 451 -> 491

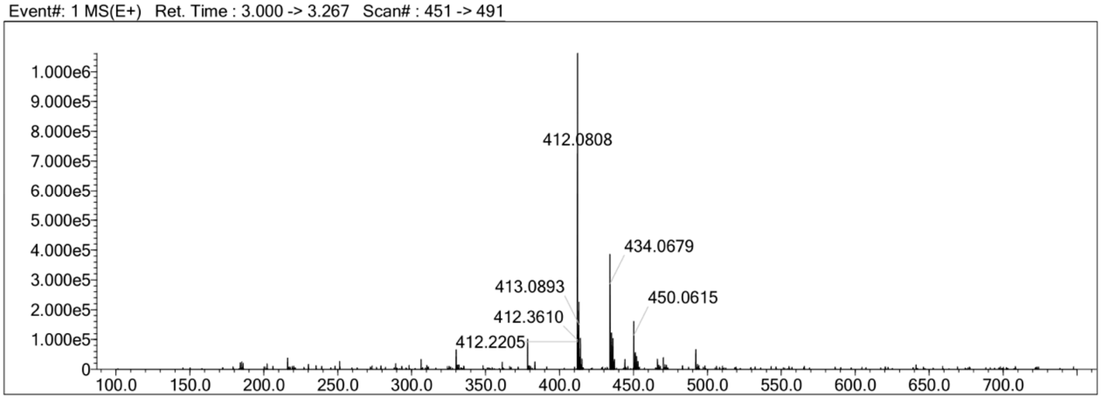

Measured region for 412.0808 m/z

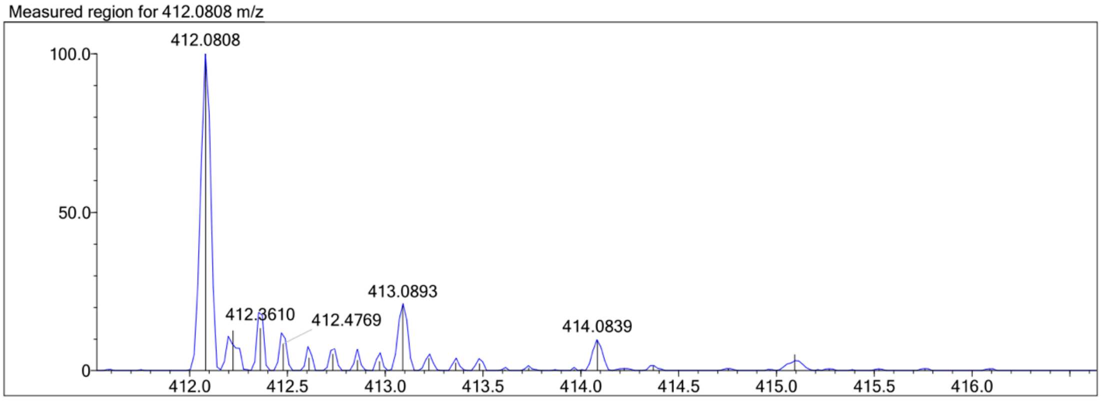

C17 H21 N3 O3 S3 [M+H]+ : Predicted region for 412.0818 m/z

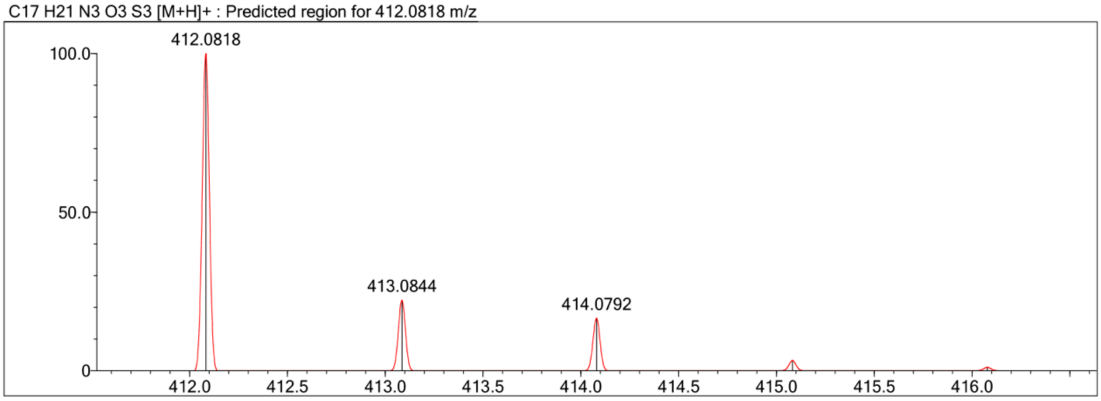

Rank	Score	Formula (M)	Ion	Meas. m/z	Pred. m/z	Df. (mDa)	Df. (ppm)	Iso	DBE
1	58.21	C17 H21 N3 O3 S3	[M+H]+	412.0808	412.0818	-1.0	-2.43	60.37	9.0

Figure S21. HRMS spectrum of compound **4i**

Formula Predictor Report - 1_21.04.2026_FPO-9_3.lcd

Data File: C:\LabSolutions\Data\Analiz\AEE\1_21.04.2026_FPO-9_3.lcd

Elmt	Val.	Min	Max	Elmt	Val.	Min	Max	Elmt	Val.	Min	Max	Elmt	Val.	Min	Max	Use Adduct
H	1	10	33	O	2	1	5	S	2	0	3	Ru	2	0	0	H
C	4	10	21	F	1	0	0	Cl	1	0	1	Pd	2	0	0	
N	3	3	6	P	3	0	0	Br	1	0	0	I	3	0	0	

Error Margin (ppm):	5	DBE Range:	8.0 - 30.0	Electron Ions:	both
HC Ratio:	unlimited	Apply N Rule:	yes	Use MSn Info:	yes
Max Isotopes:	5	Isotope RI (%):	1.00	Isotope Res:	9000
MSn Iso RI (%):	10.00	MSn Logic Mode:	AND	Max Results:	50

Event#: 1 MS(E+) Ret. Time : 2.547 -> 2.707 Scan# : 383 -> 407

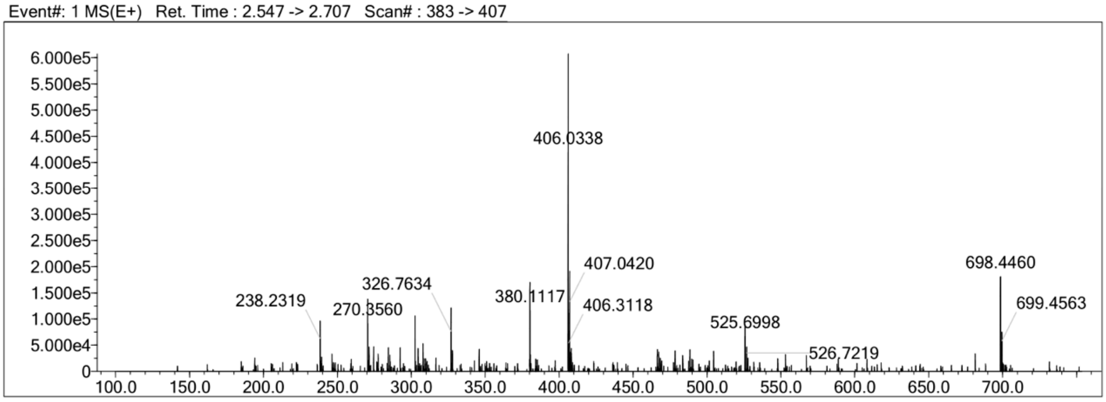

Measured region for 406.0338 m/z

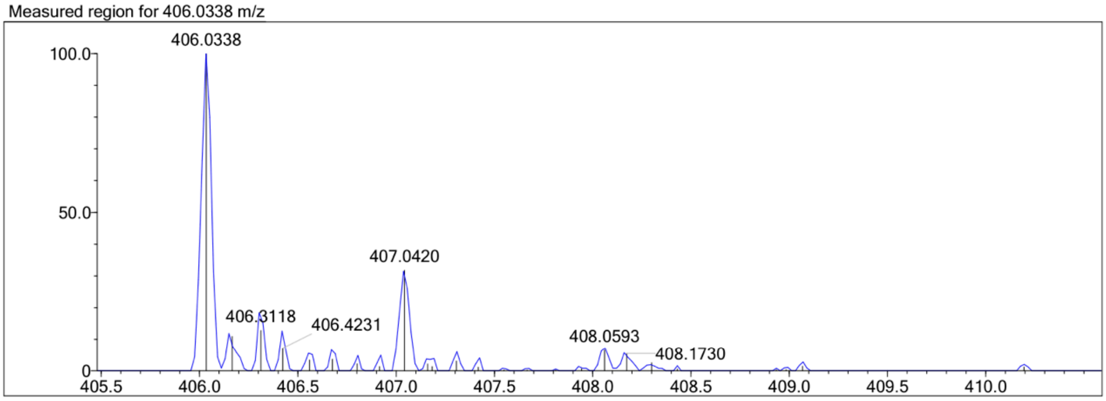

C17 H15 N3 O3 S3 [M+H]+ : Predicted region for 406.0348 m/z

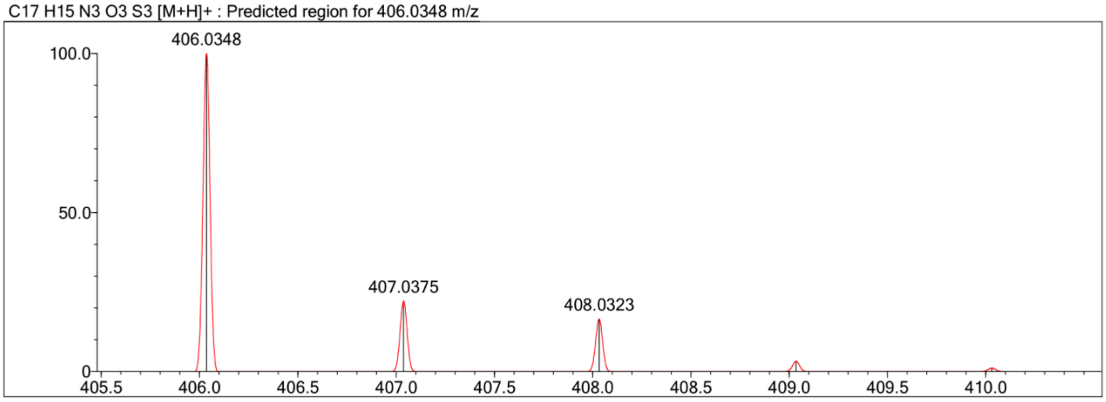

Rank	Score	Formula (M)	Ion	Meas. m/z	Pred. m/z	Df. (mDa)	Df. (ppm)	Iso	DBE
1	14.48	C17 H15 N3 O3 S3	[M+H]+	406.0338	406.0348	-1.0	-2.46	15.02	12.0

Figure S22. HRMS spectrum of compound **4j**

Formula Predictor Report - FPO-10__61.lcd

Data File: C:\LabSolutions\Data\Analiz\Derya\2\FPO-10__61.lcd

Elmt	Val.	Min	Max	Elmt	Val.	Min	Max	Elmt	Val.	Min	Max	Elmt	Val.	Min	Max	Use Adduct
H	1	10	33	O	2	0	5	S	2	0	3	Ru	2	0	0	H
C	4	10	30	F	1	0	0	Cl	1	0	0	Pd	2	0	0	
N	3	3	6	P	3	0	0	Br	1	0	0	I	3	0	0	

Error Margin (ppm): 5
HC Ratio: unlimited
Max Isotopes: 5
MSn Iso RI (%): 10.00

DBE Range: 8.0 - 30.0
Apply N Rule: yes
Isotope RI (%): 1.00
MSn Logic Mode: AND

Electron Ions: both
Use MSn Info: yes
Isotope Res: 9000
Max Results: 50

Event#: 1 MS(E+) Ret. Time : 3.213 -> 3.627 Scan# : 483 -> 545

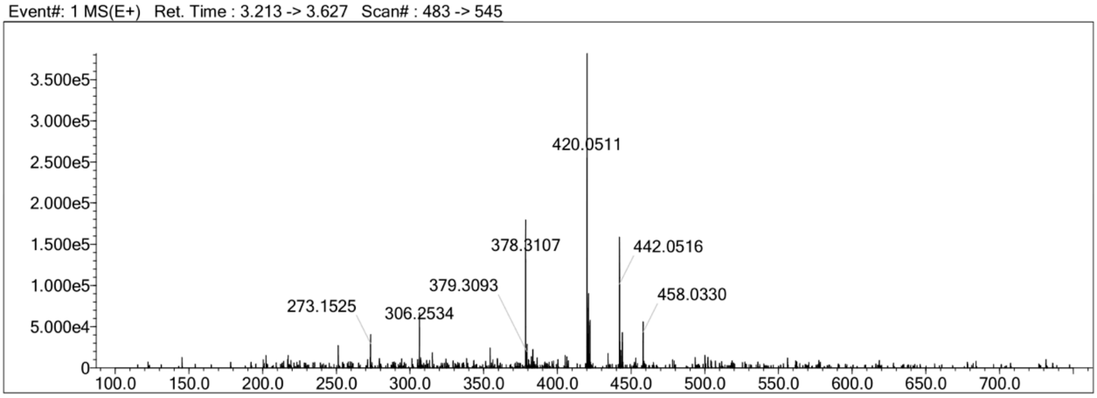

Measured region for 420.0511 m/z

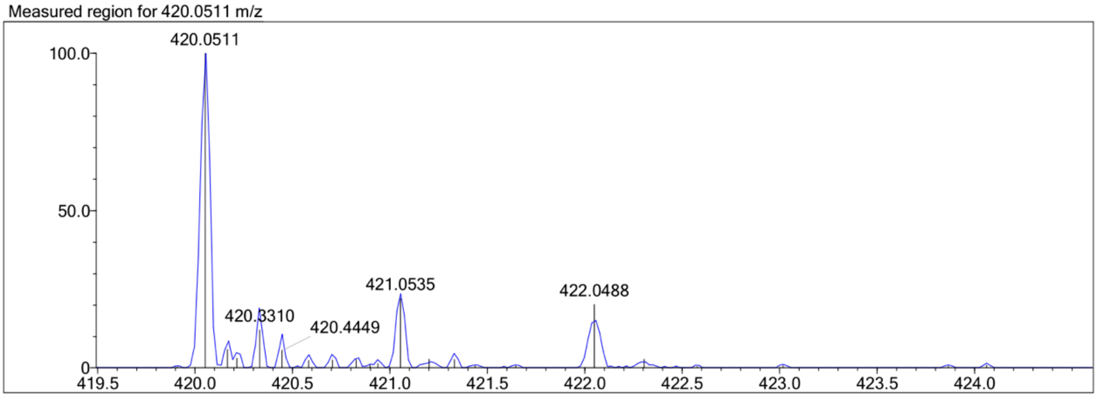

C18 H17 N3 O3 S3 [M+H]+ : Predicted region for 420.0505 m/z

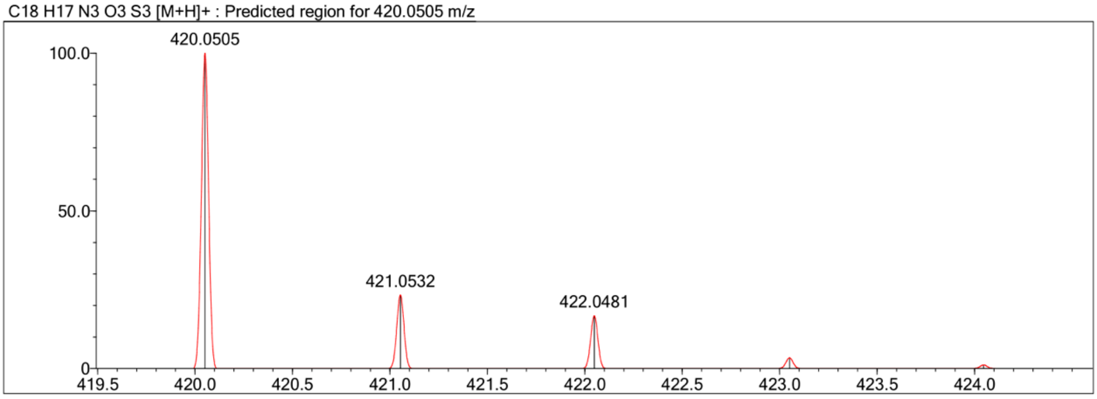

Rank	Score	Formula (M)	Ion	Meas. m/z	Pred. m/z	Df. (mDa)	Df. (ppm)	Iso	DBE
1	49.18	C18 H17 N3 O3 S3	[M+H]+	420.0511	420.0505	0.6	1.43	49.72	12.0

Figure S23. HRMS spectrum of compound **4k**

Data File: C:\LabSolutions\Data\Analiz\AEE\1_21.04.2026_FPO-11_1.lcd

Elmt	Val.	Min	Max	Elmt	Val.	Min	Max	Elmt	Val.	Min	Max	Elmt	Val.	Min	Max	Use Adduct
H	1	10	33	O	2	1	5	S	2	0	3	Ru	2	0	0	H
C	4	10	21	F	1	0	0	Cl	1	0	0	Pd	2	0	0	
N	3	3	6	P	3	0	0	Br	1	0	0	I	3	0	0	

Error Margin (ppm):	5	DBE Range:	8.0 - 30.0	Electron Ions:	both
HC Ratio:	unlimited	Apply N Rule:	yes	Use MSn Info:	yes
Max Isotopes:	5	Isotope RI (%):	1.00	Isotope Res:	9000
MSn Iso RI (%):	10.00	MSn Logic Mode:	AND	Max Results:	50

Event#: 1 MS(E+) Ret. Time : 2.853 -> 2.973 - 3.360 -> 3.757 Scan# : 429 -> 447 - 505 -> 565

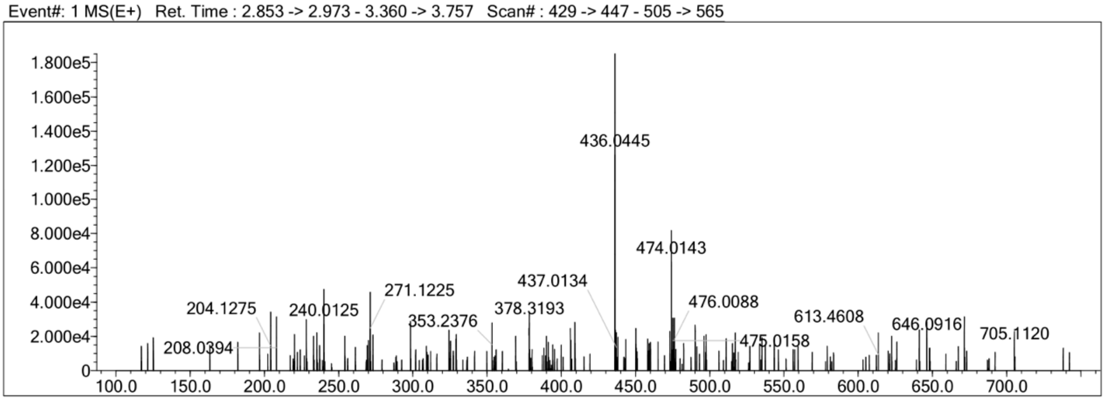

Measured region for 436.0445 m/z

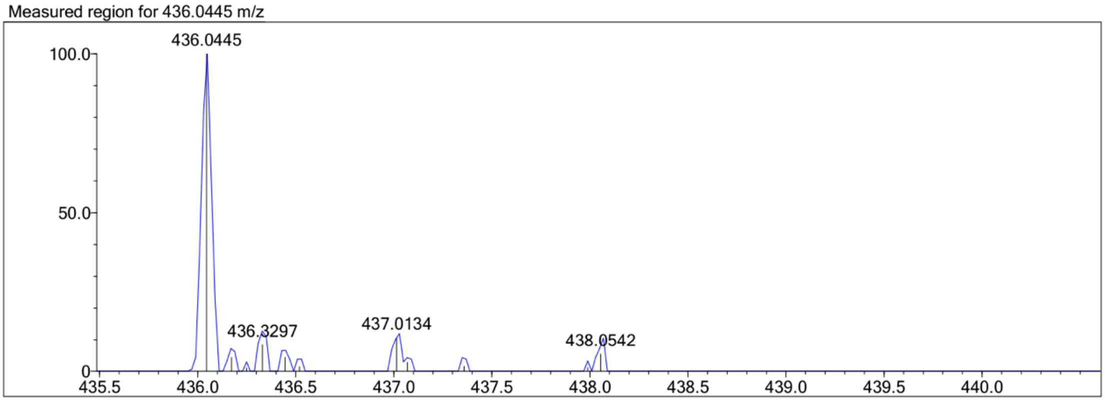

C18 H17 N3 O4 S3 [M+H]+ : Predicted region for 436.0454 m/z

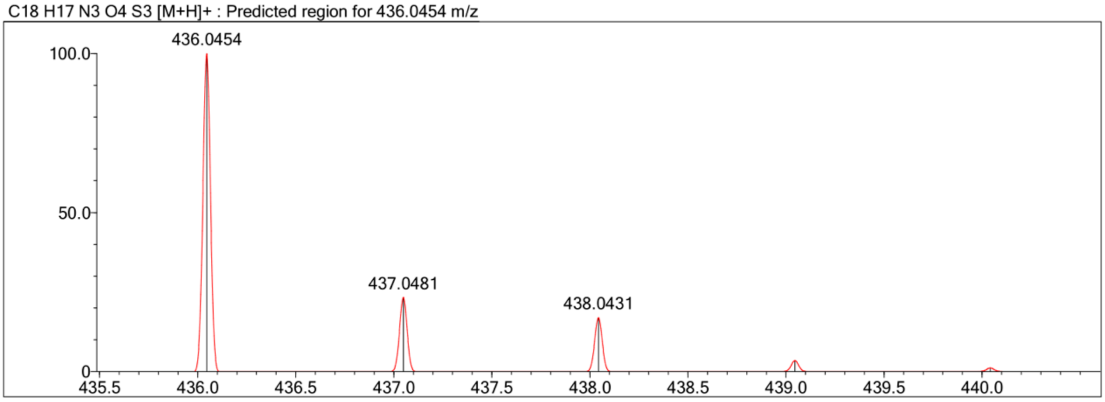

Rank	Score	Formula (M)	Ion	Meas. m/z	Pred. m/z	Df. (mDa)	Df. (ppm)	Iso	DBE
1	0.00	C18 H17 N3 O4 S3	[M+H]+	436.0445	436.0454	-0.9	-2.06	0.00	12.0

Figure S24. HRMS spectrum of compound **4l**

Formula Predictor Report - 1_21.04.2026_FPO-12_4.lcd   Page 1 of 1

Data File: C:\LabSolutions\Data\Analiz\AEE\1_21.04.2026_FPO-12_4.lcd

Elmt	Val.	Min	Max	Elmt	Val.	Min	Max	Elmt	Val.	Min	Max	Elmt	Val.	Min	Max	Use Adduct
H	1	10	33	O	2	1	5	S	2	0	3	Ru	2	0	0	H
C	4	10	21	F	1	0	0	Cl	1	0	1	Pd	2	0	0	
N	3	3	6	P	3	0	0	Br	1	0	0	I	3	0	0	

Error Margin (ppm): 5
HC Ratio: unlimited
Max Isotopes: 5
MSn Iso RI (%): 10.00

DBE Range: 8.0 - 30.0
Apply N Rule: yes
Isotope RI (%): 1.00
MSn Logic Mode: AND

Electron Ions: both
Use MSn Info: yes
Isotope Res: 9000
Max Results: 50

Event#: 1 MS(E+) Ret. Time : 3.507 -> 3.840 Scan# : 527 -> 577

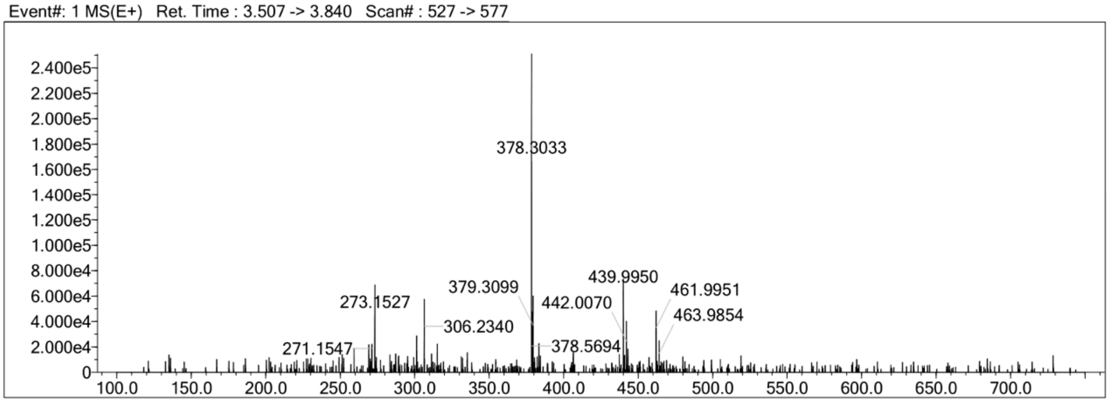

Measured region for 439.9950 m/z

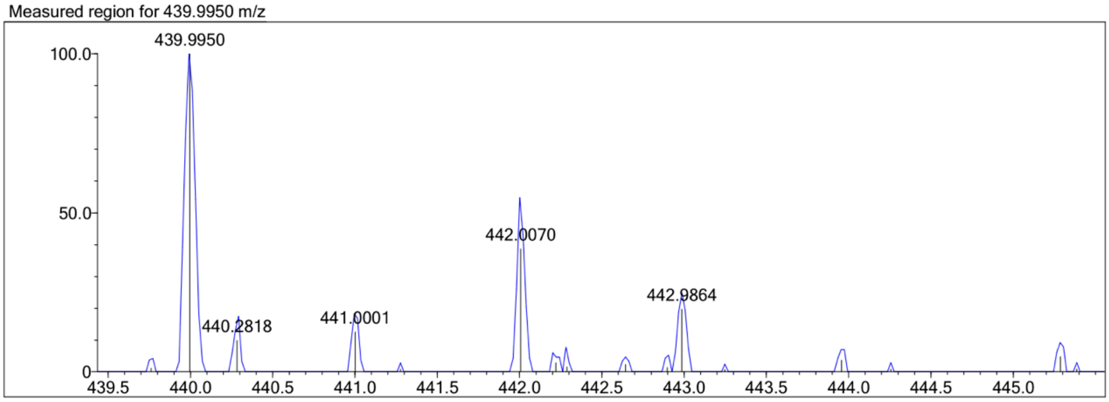

C17 H14 N3 O3 S3 Cl [M+H]+ : Predicted region for 439.9959 m/z

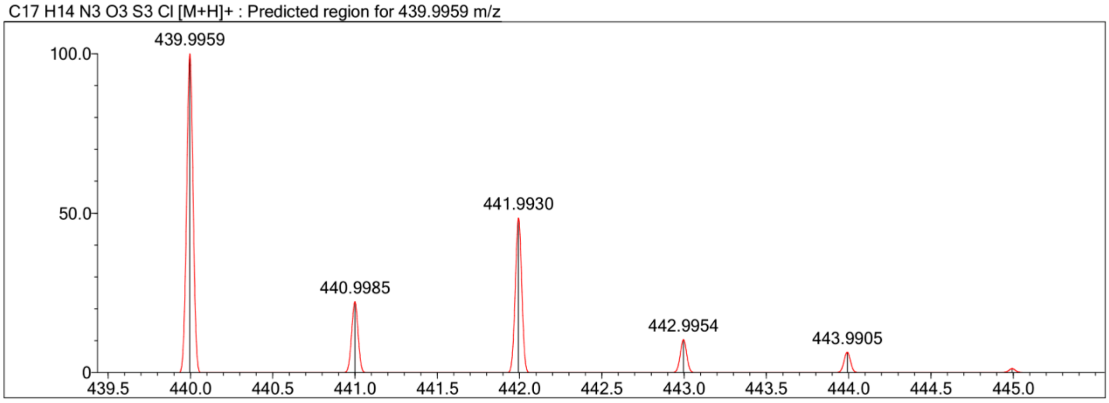

Rank	Score	Formula (M)	Ion	Meas. m/z	Pred. m/z	Df. (mDa)	Df. (ppm)	Iso	DBE
1	72.68	C17 H14 N3 O3 S3 Cl	[M+H]+	439.9950	439.9959	-0.9	-2.05	74.64	12.0

Figure S25. Two-dimensional (2D) representation of the interactions of compounds **4e, 4f** and **4g** with the active site of the DAT (PDB ID: 4XNX)

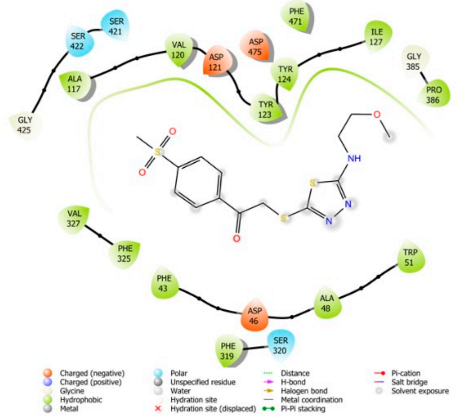

Compound 4e 2D pose with PDB ID:4XNX

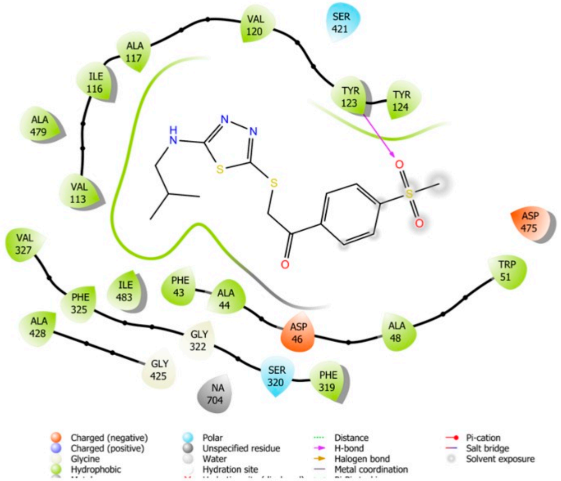

Compound 4g 2D pose with PDB ID:4XNX

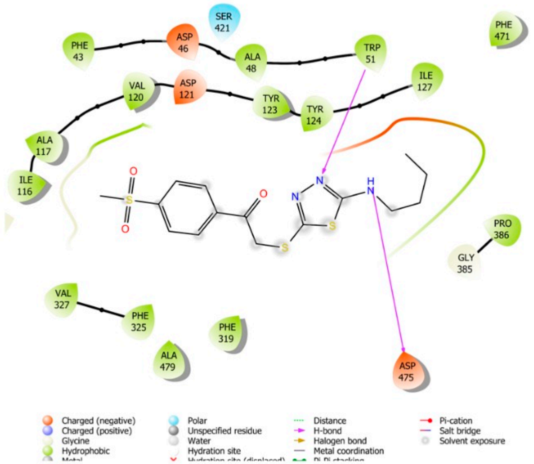

Compound 4f 2D pose with PDB ID:4XNX

Figure S26. Two-dimensional (2D) representation of the interactions of compounds **4h** and **4i** with the active site of the DAT (PDB ID: 4XNX)

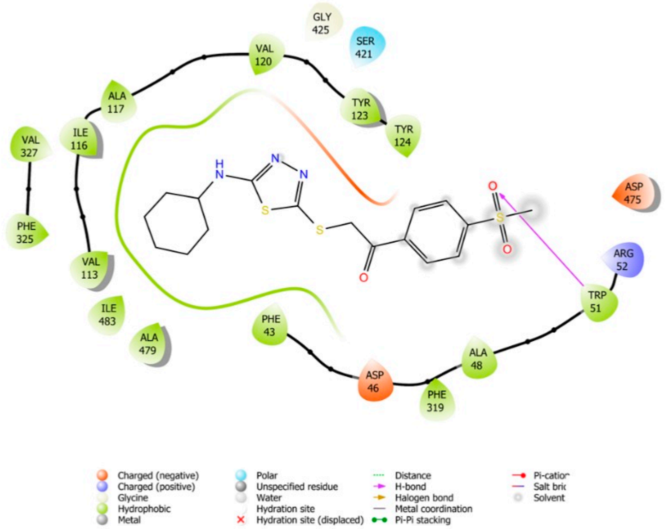

Compound 4h 2D pose with PDB ID:4XNX

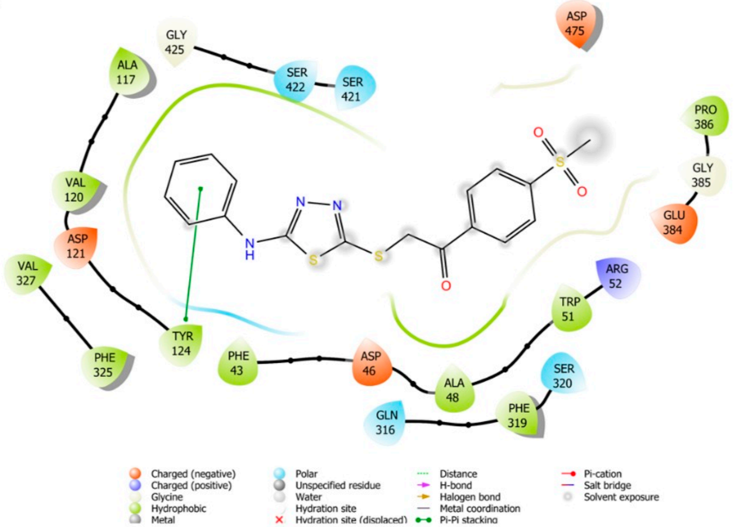

Compound 4i 2D pose with PDB ID:4XNX

Figure S27. Two-dimensional (2D) representation of the interactions of vilazadone with the active site of the SERT (PDB ID: 7LWD)

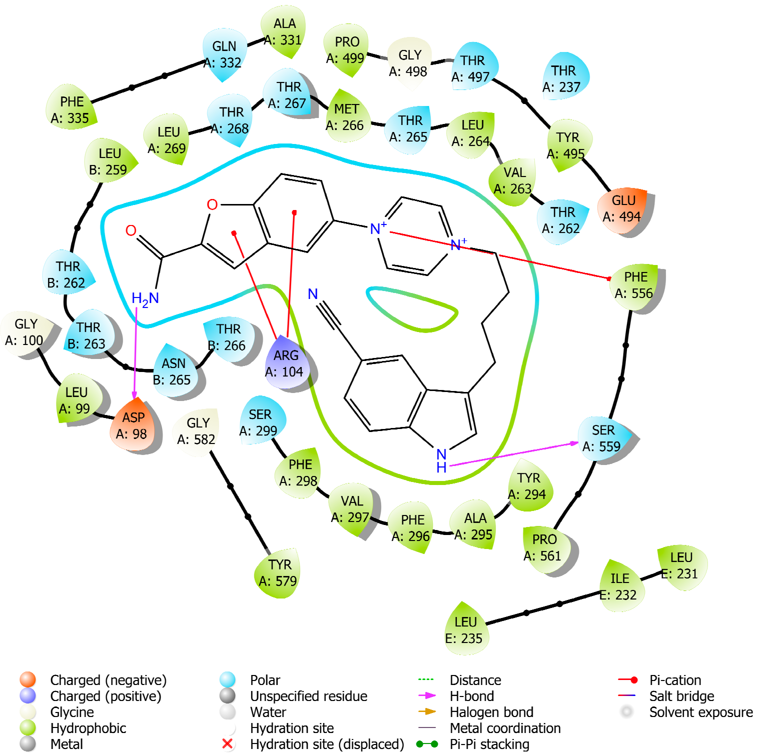

Figure S28. Two-dimensional (2D) representation of the interactions of alprazolam with the active site of the GABA-A receptor (PDB ID: 6HUO)

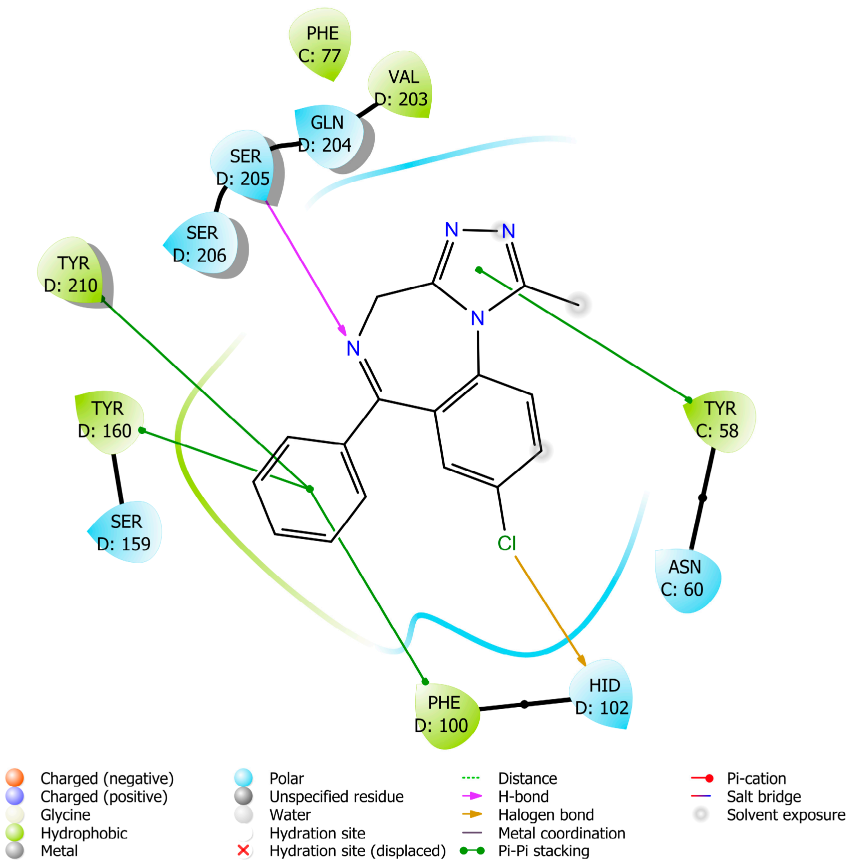

Figure S29. Two-dimensional (2D) representation of the interactions of aripiprazole with the active site of the 5HT1A receptor (PDB ID: 7E2Z)

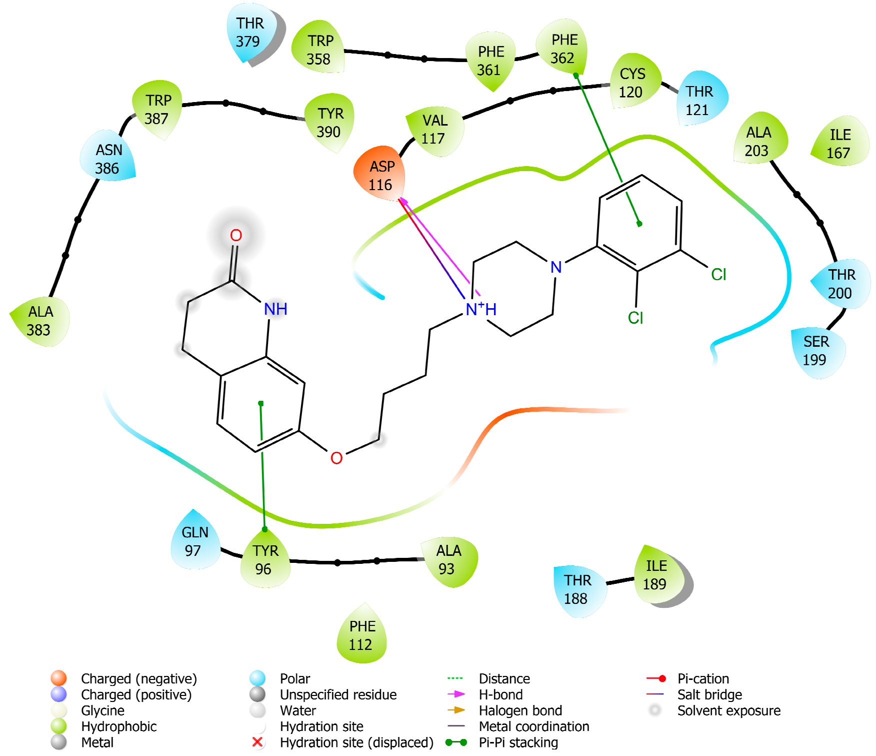

Supplement: Supplementary file 1 [file pharmaceuticals-19-00797-s001.zip › pharmaceuticals-4252148-supplementary.pdf]
